# Design, Synthesis, and Biological Activity Studies of Aldisine Derivatives Containing Acylhydrazone Moiety

**DOI:** 10.3390/ijms26178308

**Published:** 2025-08-27

**Authors:** Wentao Xu, Kangkang Yang, Mingxing Li, Longqi Li, Fuqiao Xing, Jiayi Li, Yuxiu Liu, Jingjing Zhang, Qingmin Wang, Hongjian Song

**Affiliations:** 1 State Key Laboratory of Elemento-Organic Chemistry, Frontiers Science Center for New Organic Matter, College of Chemistry, Nankai University, Tianjin 300071, China; xwt391@126.com (W.X.); liuyuxiu@nankai.edu.cn (Y.L.); chemzhangjingjing@163.com (J.Z.); 2 College of Basic Science, Tianjin Agricultural University, Tianjin 300384, China

**Keywords:** aldisine, acylhydrazone, anti-TMV, fungicidal, insecticidal

## Abstract

Marine natural products have gained increasing interest in drug research and development because of their unique structures, diverse biological activities, and novel mechanisms of action. Using the antiviral alkaloid aldisine as the lead compound and utilizing the hydrogen bond effects common in drug design, novel derivatives containing an acylhydrazone moiety were designed and synthesized. The structures of these derivatives were systematically analyzed using variable-temperature ^1^H-NMR. Antiviral activity tests showed that most derivatives were active against tobacco mosaic virus (TMV), with some compounds outperforming the commercial antiviral drug ribavirin. Notably, 3-methylphenyl- and 3-pyridyl-substituted acylhydrazones **5-6** and **5-12** displayed activity comparable to ningnanmycin, one of the most effective commercial antiviral agents. Molecular docking results indicated that incorporating the acylhydrazone moiety enhances hydrogen bonding between the molecules and target proteins. Additionally, we evaluated the fungicidal and larvicidal activities of these derivatives. Most exhibited significant larvicidal effects against *Mythimna separata* and *Plutella xylostella*, along with broad-spectrum fungicidal activity. Four related compounds (**5-11**, **5-12**, **5-13**, and **5-17**) exhibited high fungicidal activities, and another four compounds (**2-4**, **5-6**, **5-13**, and **5-17**) exhibited high larvicidal activities.

## 1. Introduction

In the global agricultural production system, plant viral diseases have emerged as one of the primary biological threats to crop health and food security [1]. According to statistics from the Food and Agriculture Organization of the United Nations, annual crop yield losses caused by plant viral diseases worldwide exceed USD 60 billion, severely impeding the sustainable development of agricultural economies [2,3,4,5]. Compared with fungal and bacterial pathogens, plant viruses are characterized by rapid dissemination, subtle symptom manifestation, and challenging control strategies [6]. Currently, commercially available anti-plant virus agents are limited in variety, with poor field control efficacy and weak therapeutic effects. Thus, there is an urgent need to develop new and highly effective anti-plant virus drugs [7,8,9,10].

Natural products have gradually emerged as a crucial resource pool for the creation of new pesticides, owing to their structural diversity, environmental compatibility, and unique mechanisms of action [11,12,13]. Derived from plants, animals, and microorganisms, natural products such as alkaloids, terpenoids, and polypeptides exhibit significant biological activities by inhibiting the growth and development of pests, interfering with the metabolism of pathogens, and more. Additionally, these substances possess characteristics such as low toxicity and easy degradability, which align well with the growing demand for environmentally friendly pest control technologies in modern agriculture [14,15].

The vast marine ecosystem harbors a wealth of natural products, which serve as potential model molecules for new drug development [16,17]. Over the past decade, with more than 1000 new structures discovered annually, this period has emerged as a golden era for marine natural product exploration, culminating in the establishment of a highly valuable yet extremely large chemical database. Among these, compounds featuring ultra-novel skeletons and/or exceptional bioactivities stand as paradigms of the most promising candidates [18]. In our preliminary work, we found that multiple marine natural products exhibit anti-plant virus activity, with aldisine alkaloids in particular showing relatively strong antiviral activity [19].

Hydrazone compounds are a class of compounds with broad-spectrum biological activities. As the core structure of hydrazone compounds, the hydrazone moiety (-CONHN=CH-) is often incorporated into the skeletons of drug molecules to serve as an active group [20,21,22]. Owing to its multiple hydrogen-bonding sites, it can enhance hydrogen-bond interactions between small molecules and target proteins, thereby improving the biological activity of the compounds [23]. In addition, during the derivatization of β-tetrahydrocarboline by our research group, it was found that introducing the hydrazone structure into the carboline ring significantly enhances the anti-TMV (tobacco mosaic virus) activity of the corresponding derivatives [24,25]. Our research group also found that introducing the hydrazone structure into oxindole spirocyclic compounds can also considerably improve the anti-TMV activity of such derivatives [26,27].

**Figure 1:**
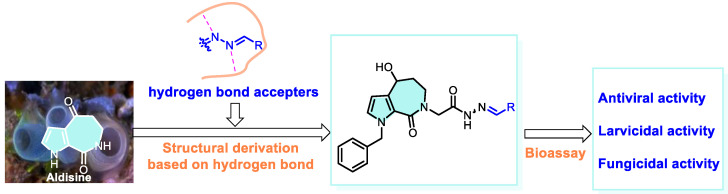
Design of the target compounds.

To enhance hydrogen–bond–mediated interactions between the target molecule and protein, a novel series of derivatives incorporating an acylhydrazone pharmacophore were rationally designed and synthesized (Figure 1). These compounds were subsequently subjected to systematic evaluation of their anti-tobacco mosaic virus (anti-TMV) activity, representing the first reported investigation of this molecular series against TMV. Furthermore, to explore broader agrochemical utility, the synthesized aldisine derivatives were additionally assessed for larvicidal and fungicidal properties through standardized bioassays.

## 2. Results and Discussion

### 2.1. Synthesis

**Scheme 1:**
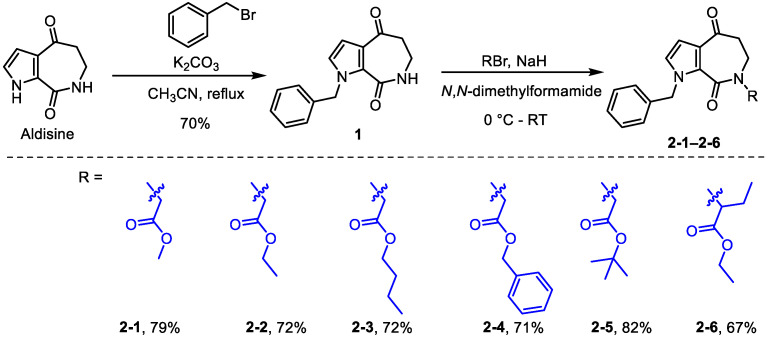
Synthesis of aldisine derivatives **2-1** to **2-6**.

Under alkaline conditions, aldisine underwent a regioselective nucleophilic substitution reaction with benzyl bromide, forming derivative **1**. Subsequently, using sodium hydride as the base, derivative **1** reacted with α-bromoesters in a nucleophilic substitution reaction to produce the alkoxycarbonyl methyl-substituted derivatives from **2-1** to **2-6** (Scheme 1).

**Scheme 2:**
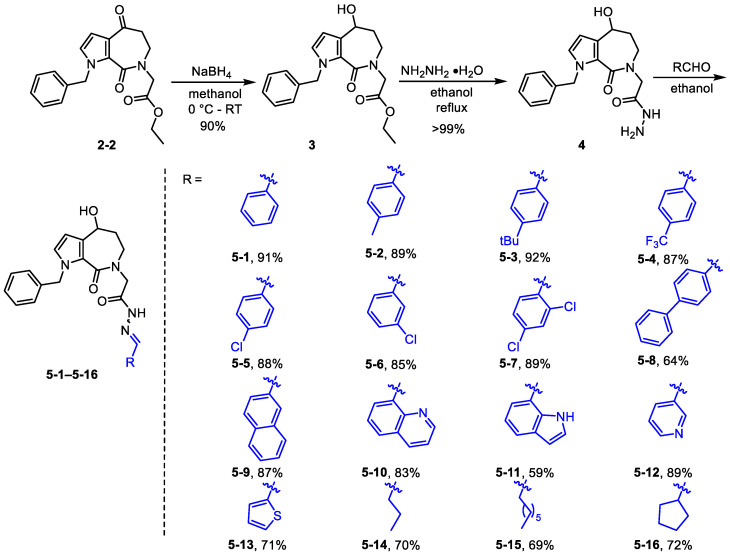
Synthesis of aldisine derivatives from **5-1** to **5-16**.

Starting with aldisine derivative **2-2**, alcohol derivative **3** was produced through reduction with sodium borohydride. The ester group in alcohol **3** underwent amide formation with hydrazine to produce the acylhydrazide derivative **4**. Next, derivative **4** underwent a condensation reaction with aromatic or aliphatic aldehydes to create hydrazone derivatives from **5-1** to **5-16** (Scheme 2).

**Scheme 3:**
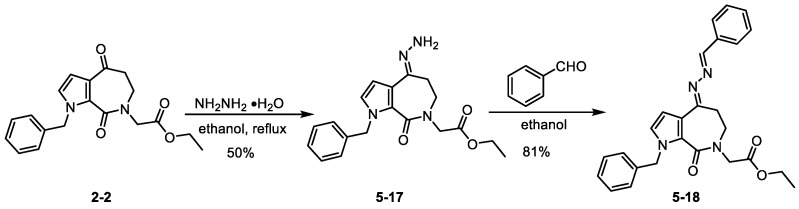
Synthesis of aldisine derivatives from **5-17** to **5-18**.

Different types of hydrazone derivative **5-17** were synthesized through a condensation reaction of derivative **2-2** with hydrazine hydrate under heating and reflux conditions. Subsequently, this hydrazone underwent a condensation reaction with benzaldehyde to yield azine derivative **5-18** (Scheme 3).

**Scheme 4:**
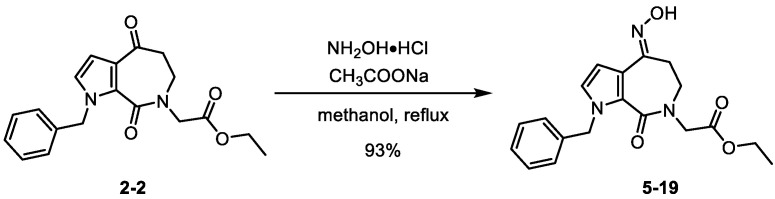
Synthesis of aldisine derivative **5-19**.

Oxime derivative **5-19** was synthesized via a condensation reaction of derivative **2-2** with hydroxylamine hydrochloride under heating and reflux conditions (Scheme 4).

During the synthesis process, it was found that when compound **2-2** was used as the starting compound to react with hydrazine hydrate, the expected ester hydrazine formation product was not obtained, and the resulting product was the hydrazone compound **5-17**. This experimental phenomenon suggests that the reactivity of the ketone carbonyl group is significantly higher than that of the ester carbonyl group, leading it to react preferentially with hydrazine hydrate.

To further verify the above conclusion, the carbonyl group in compound **2-2** was reduced to obtain compound **3**, and then compound **3** was subjected to a hydrazine formation reaction, which successfully obtained the ester hydrazine formation product **4** quantitatively. The experimental results further confirm that the reactivity of the ketone carbonyl group is higher than that of the ester group. That is, after the ketone carbonyl group was reduced, the ester carbonyl group could smoothly undergo a hydrazine formation reaction with hydrazine hydrate.

### 2.2. Configuration

**Figure 2:**
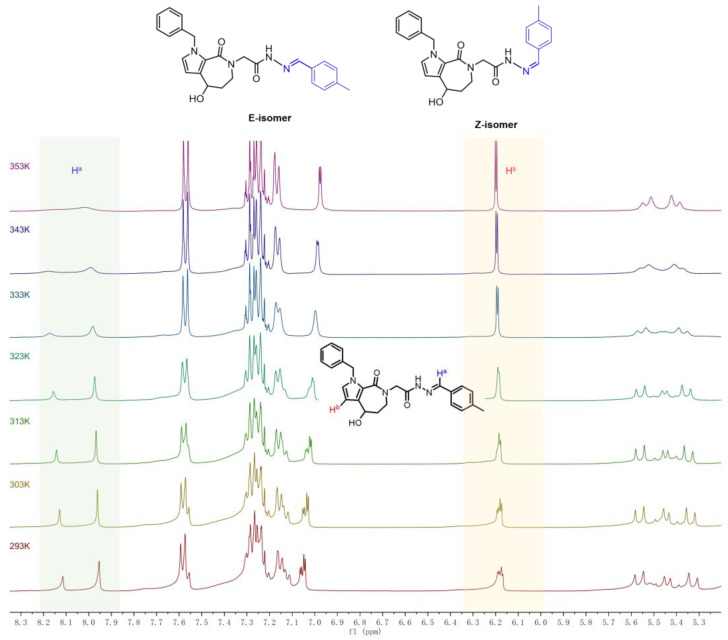
Variable temperature NMR of **5-2**.

At room temperature, the proton NMR and carbon NMR spectra of all synthesized acylhydrazone compounds (**5-1**–**5-16**) exhibited two sets of peaks. We propose that the possible reasons for this phenomenon are the *E*-*Z* isomerism of the acylhydrazone chain, specifically the mixture of (4*RS*, *E*)- and (4*RS*, *Z*)-configurations. It is generally believed that the *E*-isomer is the product of thermodynamic control. Kinetic control is governed by activation energy (favoring faster formation), while thermodynamic control is governed by product stability (favoring the lower-energy isomer at equilibrium). Variable-temperature NMR experiments (293–353 K) were conducted to clarify the mechanism (Figure 2). For compound **5-2**, the proton signal of H^b^ (pyrrole 3-position hydrogen) in the ^1^H-NMR spectrum gradually transformed from a quartet (two sets of peaks) to a doublet (one set of peaks) with increasing temperature (Figure 2). Meanwhile, the proton signal of H^a^ (aldimine hydrogen) gradually changed from a doublet (two sets of peaks) to a singlet (one set of peaks) as the temperature rose. These results confirmed that the *E*-*Z* isomer mixture of **5-2** converted to a single *E*-isomer during heating, indicating that the *E*-*Z* isomerism of the acylhydrazone chain is responsible for the appearance of two sets of peaks in the NMR spectra at room temperature.

### 2.3. Anti-TMV Activities

Using ribavirin and ningnanmycin as controls, we first studied the synthesized derivatives’ anti-tobacco mosaic virus (TMV) activity (Table 1). Bioassay results showed that most of these derivatives showed anti-TMV activity. Derivatives from **2-2**, **2-6**, **3**, **4**, **5**, **5-3**, **5-6**, and **5-11** to **5-13**, and **5-15** to **5-19** showed higher anti-TMV activity than that of the commercial antiviral drug ribavirin (inhibition rate for inactivation, curative, and protection activities in vivo: 38 ± 2, 40 ± 3, and 41 ± 3% at 500 mg/L); in particular, the anti-TMV activities of derivatives **5-6** and **5-12** were comparable to that of the most effective commercial antiviral drug, ningnanmycin (57 ± 4, 56 ± 2, and 59 ± 1% at 500 mg/L).

For ester derivatives **2**, the structure of the ester group has a significant impact on their activity. Studies have shown that ethyl ester (**2-2**) and α-ethyl ethyl ester (**2-6**) exhibit the best activity, while altering the chain length of the alkoxy group in the ester moiety or introducing substituents will have an adverse effect on the activity. Reducing the ketone carbonyl group in ester derivative **2-2** to a hydroxyl group (**3**) is beneficial for enhancing its activity; further converting the ester group into a hydrazide derivative **4** via hydrazinolysis results in the activity remaining essentially unchanged.

For acyhydrazone derivatives, the substituent R has a significant impact on their activity. When R is a phenyl group (**5-1** to **5-16**), the type, position, and number of substituents on the benzene ring all exert a certain influence on the activity: among them, the para-substituted derivative with a tert-butyl group (**5-3**) exhibits the best activity, while the meta-substituted derivative (**5-6**) shows better activity than the para-substituted one (**5-5**). When R is another aromatic ring (**5-9**) or heteroaromatic ring (**5-10** to **5-13**), the pyridine ring derivative **5-12** displays the optimal activity, which is comparable to the antiviral activity of ningnanmycin. When R is an aliphatic substituent, most compounds (**5-14** to **5-16**) show good biological activity, and their activity is equivalent to that of ribavirin.

Based on derivative **2-2**, derivatization of its ketone carbonyl group was performed, and the resulting hydrazone (**5-17**), bis-hydrazone (**5-18**), and oxime (**5-19**) derivatives exhibited activity comparable to that of derivative **2-2**. These results indicate that such derivatization modification of the ketone carbonyl group at this site has little effect on the activity.

### 2.4. Phytotoxicity

The results of the phytotoxicity assay demonstrated that these target compounds exhibited no phytotoxic effects on Nicotiana benthamiana at a concentration of 500 mg/L.

### 2.5. Molecular Docking Research

**Figure 3:**
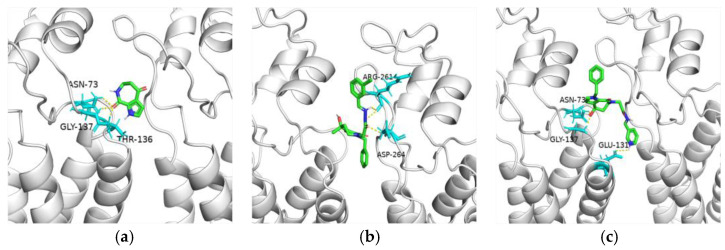
Molecular docking results of aldisine (**a**), compound **5-6** (**b**), and **5-12** (**c**) with TMV CP. In the figure, silver gray represents the capsid protein, light blue represents amino acid residues, and the remaining colors represent small molecules.

Previous studies have confirmed that aldisine can target TMV capsid proteins [27]. Based on this research finding, the present study further explores the possible interactions between the highly active compounds **5-6** and **5-12** and the capsid protein (PDB code: 1EI7), which were molecularly docked using Auto Dock 4.2 (Figure 3). The experimental results indicate that there are significant differences in the ways different compounds form hydrogen bond interactions with the capsid protein. Specifically, in their interactions with the capsid protein, the highly active compounds **5-6** and **5-12** can form hydrogen bonds with more types of amino acid residues through their respective specific structural sites. Among them, **5-6** interacts with ARG-261 and ASP-263 via its acylhydrazone moiety, while **5-12** binds to ASN-73, GLY-137, and GLU-131 through its ketone carbonyl group and the nitrogen atom on the pyridine ring, respectively. In contrast, aldisine can only form hydrogen bonds with the amino acid residues of the capsid protein, relying on its ketone carbonyl group, showing a relatively single mechanism of hydrogen bond interaction. This difference suggests that **5-6** and **5-12** may enhance their binding ability to the capsid protein through more diverse hydrogen bond interaction modes, providing a potential structural basis for their high activity characteristics.

### 2.6. Insecticidal Activities

Next, we examined whether the alkaloid derivatives had larvicidal activity. Overall, most derivatives showed insecticidal activity against larvae of lepidopteran pests such as *M. separata* and *P. xylostella* (Table 2), and most were more active than aldisine. Regarding larvicidal activity against *M. separata*, acylhydrazone derivatives generally demonstrated better effects than derivatives of other structural types, with compounds **5-3** and **5-8** showing 30% insecticidal activity at a concentration of 100 mg/kg. For *P. xylostella*, derivatization of the alkaloid improved activity. Derivatives **2-4**, **5-6**, **5-13**, and **5-17** showed over 90% larvicidal activity at 600 mg/L. Both acylhydrazone **5-13** and hydrazone **5-17** exhibited 30% insecticidal activity against *P. xylostella* at 100 mg/kg.

### 2.7. Fungicidal Activity

Finally, we examined the anti-phytopathogenic activity of these derivatives. The fungicidal effects of these compounds against 14 different phytopathogenic fungi were assessed using the mycelial growth method (Table 3). Most derivatives demonstrated fungicidal activity at 50 mg/L against the 14 plant pathogens. These derivatives displayed selectivity, as most had high fungicidal effects against *P. piricola*. Ester derivatives **2-2** and **2-6**, acylhydrazone derivatives **5-7**, **5-9**, **5-11**, **5-13**, and **5-16**, along with hydrazone derivatives **5-17** and **5-18**, showed more than 50% bactericidal activity against over five plant pathogenic fungi at 50 mg/kg. Some compounds exhibited specific and excellent fungicidal activity against certain fungi, such as ester derivative **2-2**, which showed an 88 ± 1% inhibition rate against *R. solani*; acylhydrazone derivatives **5-11** and **5-12**, which exhibited 90 ± 1% and 85 ± 2% inhibition rates against *B. cinerea*, respectively; and acylhydrazone derivative **5-13** and hydrazone derivative **5-17**, which demonstrated 81 ± 1% and 86 ± 2% inhibition against *S. sclerotiorum*.

## 3. Materials and Methods

### 3.1. Instruments

^1^H and ^13^C nuclear magnetic resonance spectra were recorded using a Bruker Avance 400 Ultrashield NMR spectrometer (Bruker Corporation, Billerica, MS, USA). Chemical shifts (*δ*) are reported in parts per million (ppm) and are measured downfield from internal tetramethylsilane. High-resolution mass spectrometry (HRMS) data were obtained on an FTICR-MS instrument equipped with a q-TOF mass analyzer (Ionspec 7.0 T). Melting points were determined using an X-4 microscope melting point apparatus and are uncorrected. Conversion was monitored by thin layer chromatography (TLC). Flash column chromatography was performed on silica gel (200–300 mesh).

### 3.2. General Synthesis

Ribavirin (Topscience Co., Ltd., Shanghai, China), aldisine (Synthesized by our laboratory), ningnanmycin (Alta Scientific Co., Ltd., Tianjin, China), chlorothalonil (Bailing Agrochemical Co., Ltd., Jiangyin City, China), and other reagents were purchased from commercial sources and were used as received. All anhydrous solvents were dried and purified according to standard techniques just before use. The synthetic routes of target compounds **2**, **3**, **4**, and **5** are given in Scheme 1, Scheme 2, Scheme 3 and Scheme 4.

### 3.3. Biological Assay

The synthesized compounds’ anti-TMV, larvicidal, and fungicidal activities were tested using reported methods [28,29,30], which were described in detail in the Supporting Information. Each bioassay was repeated three times. The results are presented as means ± standard errors.

### 3.4. Phytotoxicity Assay

The target compounds were accurately weighed and dissolved in dimethylsulfoxide (DMSO), followed by dilution to 500 mg/L with 3% Tween 80 solution. The resulting target compound solutions were uniformly sprayed onto *Nicotiana benthamiana* plants, with 3% Tween 80 aqueous solution containing 0.5% DMSO serving as the blank control. The treated plants were grown in a phytotron, and their height and growth status were measured at 0, 3, 5, 7, and 10 days post-treatment. Each experiment was performed in triplicate.

## 4. Conclusions

A novel series of aldisine derivatives incorporating acylhydrazone moieties were successfully synthesized and characterized. Systematic bioactivity evaluations revealed that all compounds exhibited significant inhibitory effects against tobacco mosaic virus (TMV), with compounds **5-6** and **5-12** demonstrating particularly potent in vivo antiviral activity. Molecular docking simulations identified stable hydrogen bond interactions between these lead compounds (**5-6**/**5-12**) and key residues of TMV coat protein. The derivatives also displayed notable insecticidal activity against *M. separata* and *P. xylostella* larvae, along with broad-spectrum fungicidal effects against 14 phytopathogenic fungi.

## Figures and Tables

**Table 1:** In vivo anti-TMV activities of aldisine and its derivatives.

Compd	Inhibition Rate (%)/500 mg/L		
	Inactivation Effect	Curative Effect	Protection Effect
Aldisine	42 ± 1	42 ± 2	36 ± 3
**2-1**	21 ± 4	25 ± 4	30 ± 2
**2-2**	43 ± 1	44 ± 2	46 ± 3
**2-3**	39 ± 3	41 ± 1	43 ± 1
**2-4**	35 ± 4	29 ± 1	32 ± 1
**2-5**	40 ± 1	36 ± 3	31 ± 2
**2-6**	46 ± 4	41 ± 2	49 ± 5
**3**	48 ± 5	50 ± 2	44 ± 4
**4**	45 ± 2	41 ± 3	39 ± 4
**5-1**	40 ± 4	41 ± 1	34 ± 3
**5-2**	29 ± 3	25 ± 2	35 ± 1
**5-3**	43 ± 5	46 ± 4	47 ± 2
**5-4**	34 ± 4	33 ± 1	28 ± 4
**5-5**	20 ± 5	30 ± 1	25 ± 2
**5-6**	51 ± 1	48 ± 2	53 ± 3
**5-7**	23 ± 4	30 ± 1	25 ± 2
**5-8**	35 ± 2	38 ± 1	42 ± 3
**5-9**	32 ± 4	35 ± 4	33 ± 1
**5-10**	37 ± 2	40 ± 1	35 ± 2
**5-11**	46 ± 5	36 ± 5	42 ± 3
**5-12**	45 ± 1	50 ± 3	51 ± 4
**5-13**	52 ± 1	42 ± 1	49 ± 3
**5-14**	42 ± 2	37 ± 5	35 ± 4
**5-15**	44 ± 2	47 ± 4	44 ± 4
**5-16**	49 ± 4	39 ± 2	40 ± 5
**5-17**	45 ± 3	38 ± 4	42 ± 1
**5-18**	48 ± 1	46 ± 1	43 ± 4
**5-19**	48 ± 3	49 ± 5	42 ± 1
ningnanmycin	57 ± 4	56 ± 2	59 ± 1
ribavirin	38 ± 2	40 ± 3	41 ± 3

**Table 2:** Larvicidal activities of aldisine and its derivatives against *Mythimna separate* and *Plutella xylostella*.

Compd	*Mythimna separata*			*Plutella xylostella*		
	600	200	100	600	200	100
aldisine	20 ± 0			20 ± 0		
**2-1**	53 ± 6	20 ± 0		43 ± 6		
**2-2**	50 ± 0			53 ± 6	20 ± 0	
**2-3**	43 ± 6			56 ± 6	30 ± 0	
**2-4**	50 ± 0			93 ± 6	53 ± 6	20 ± 0
**2-5**	30 ± 0			30 ± 10		
**2-6**	50 ± 0			56 ± 6	23 ± 6	
**3**	30 ± 0			73 ± 6	40 ± 0	
**4**	100	36 ± 6		80 ± 0	43 ± 6	
**5-1**	70 ± 0	30 ± 0		66 ± 6	30 ± 0	
**5-2**	100	50 ± 0		76 ± 6	20 ± 0	
**5-3**	100	66 ± 6	30 ± 0	56 ± 6	30 ± 0	
**5-4**	50 ± 0			70 ± 0	33 ± 6	
**5-5**	23 ± 6			53 ± 6	30 ± 0	
**5-6**	100	50 ± 0		93 ± 6	63 ± 6	20 ± 0
**5-7**	36 ± 6			46 ± 6		
**5-8**	100	60 ± 0	30 ± 0	43 ± 6		
**5-9**	30 ± 0			66 ± 6	20 ± 0	
**5-10**	70 ± 0	30 ± 0		60 ± 0	10 ± 0	
**5-11**	100	30 ± 0		56 ± 6	30 ± 0	
**5-12**	50 ± 0			56 ± 6	23 ± 6	
**5-13**	63 ± 6	10 ± 0		90 ± 0	56 ± 6	30 ± 0
**5-14**	100	50 ± 0		60 ± 0	20 ± 0	
**5-15**	66 ± 6	30 ± 0		70 ± 10	30 ± 0	
**5-16**	70 ± 0	20 ± 0		66 ± 6	30 ± 0	
**5-17**	50 ± 0			90 ± 0	56 ± 6	30 ± 0
**5-18**	100	46 ± 6		90 ± 0	50 ± 0	
**5-19**	40 ± 0			66 ± 6	30 ± 0	

**Table 3:** Activities of aldisine and its derivatives against fourteen kinds of fungi.

Compd	Inhibition Rate (% at 50 mg/L)													
	*A.S.*	*F.G.*	*P.G.*	*P.C.*	*S.S*	*B.C.*	*R.S.*	*F.C.*	*C.H.*	*P.P.*	*R.C.*	*B.M.*	*W.A.*	*F.M.*
aldisine	37 ± 1	32 ± 1	10 ± 1	23 ± 1	25 ± 1	19 ± 1	20 ± 2	19 ± 1	25 ± 1	40 ± 2	37 ± 1	18 ± 1	18 ± 1	31 ± 2
**2-1**	27 ± 2	20 ± 2	25 ± 2	19 ± 1	32 ± 2	14 ± 1	25 ± 1	24 ± 2	32 ± 1	69 ± 2	25 ± 1	26 ± 2	31 ± 1	47 ± 2
**2-2**	50 ± 1	57 ± 2	44 ± 1	34 ± 2	32 ± 2	38 ± 1	88 ± 1	37 ± 1	36 ± 3	50 ± 1	53 ± 1	40 ± 0	43 ± 1	70 ± 2
**2-3**	44 ± 2	20 ± 1	37 ± 2	13 ± 1	42 ± 2	14 ± 1	24 ± 1	24 ± 1	32 ± 2	67 ± 2	68 ± 2	25 ± 1	33 ± 2	42 ± 2
**2-4**	44 ± 2	27 ± 2	27 ± 1	17 ± 1	32 ± 2	15 ± 1	28 ± 1	32 ± 2	57 ± 2	67 ± 2	65 ± 2	25 ± 2	44 ± 2	44 ± 2
**2-5**	39 ± 1	31 ± 2	27 ± 1	19 ± 2	32 ± 2	15 ± 1	30 ± 1	30 ± 2	70 ± 2	47 ± 1	71 ± 2	39 ± 2	45 ± 2	55 ± 2
**2-6**	39 ± 2	22 ± 1	37 ± 2	18 ± 1	21 ± 1	25 ± 1	26 ± 1	30 ± 1	76 ± 2	54 ± 1	76 ± 1	51 ± 1	51 ± 1	62 ± 1
**3**	32 ± 2	18 ± 1	23 ± 2	18 ± 1	31 ± 1	25 ± 2	21 ± 1	24 ± 2	41 ± 2	53 ± 1	35 ± 1	25 ± 1	30 ± 1	47 ± 1
**4**	61 ± 2	34 ± 1	42 ± 2	25 ± 1	45 ± 2	40 ± 2	34 ± 2	34 ± 2	34 ± 1	75 ± 2	19 ± 1	39 ± 2	30 ± 1	36 ± 1
**5-1**	53 ± 2	34 ± 2	37 ± 2	39 ± 2	27 ± 1	32 ± 1	25 ± 1	27 ± 2	45 ± 1	75 ± 2	29 ± 2	35 ± 2	32 ± 1	46 ± 2
**5-2**	32 ± 1	29 ± 2	37 ± 1	28 ± 1	79 ± 1	34 ± 1	19 ± 1	67 ± 2	13 ± 1	56 ± 2	37 ± 1	61 ± 2	32 ± 1	45 ± 1
**5-3**	41 ± 2	35 ± 1	23 ± 2	39 ± 1	39 ± 2	34 ± 1	47 ± 2	31 ± 1	45 ± 1	52 ± 1	35 ± 1	38 ± 2	39 ± 2	57 ± 2
**5-4**	52 ± 2	43 ± 1	48 ± 2	45 ± 2	25 ± 2	45 ± 2	32 ± 1	48 ± 2	44 ± 2	55 ± 1	43 ± 1	45 ± 1	43 ± 1	57 ± 2
**5-5**	40 ± 1	43 ± 2	35 ± 1	18 ± 1	37 ± 1	51 ± 1	18 ± 1	39 ± 1	55 ± 2	57 ± 2	41 ± 1	33 ± 2	44 ± 1	61 ± 2
**5-6**	50 ± 1	32 ± 1	41 ± 1	45 ± 1	30 ± 2	26 ± 1	18 ± 1	35 ± 1	34 ± 1	30 ± 2	43 ± 1	35 ± 1	39 ± 1	53 ± 2
**5-7**	50 ± 2	46 ± 2	45 ± 2	34 ± 2	39 ± 2	61 ± 2	49 ± 2	35 ± 2	54 ± 2	51 ± 2	46 ± 1	55 ± 2	47 ± 1	64 ± 2
**5-8**	30 ± 1	32 ± 2	35 ± 1	15 ± 1	20 ± 1	27 ± 1	29 ± 1	34 ± 2	41 ± 2	40 ± 1	31 ± 2	35 ± 1	34 ± 1	50 ± 2
**5-9**	35 ± 2	24 ± 1	23 ± 1	27 ± 1	34 ± 1	53 ± 2	32 ± 1	34 ± 1	51 ± 1	54 ± 1	51 ± 1	33 ± 1	34 ± 2	54 ± 1
**5-10**	70 ± 1	30 ± 1	15 ± 1	23 ± 1	22 ± 2	27 ± 1	32 ± 1	45 ± 1	45 ± 1	75 ± 2	53 ± 1	35 ± 1	32 ± 1	50 ± 2
**5-11**	60 ± 2	65 ± 2	41 ± 2	45 ± 2	22 ± 1	90 ± 1	59 ± 1	34 ± 1	55 ± 1	75 ± 1	52 ± 1	45 ± 2	44 ± 2	61 ± 2
**5-12**	60 ± 2	30 ± 1	24 ± 1	23 ± 1	25 ± 1	85 ± 2	45 ± 1	34 ± 1	51 ± 1	40 ± 1	29 ± 1	35 ± 1	39 ± 1	39 ± 1
**5-13**	55 ± 2	52 ± 1	54 ± 1	43 ± 2	81 ± 1	43 ± 2	60 ± 2	34 ± 1	41 ± 2	43 ± 2	31 ± 1	40 ± 2	34 ± 1	53 ± 2
**5-14**	40 ± 2	24 ± 1	23 ± 1	34 ± 1	15 ± 1	24 ± 1	24 ± 1	29 ± 1	27 ± 1	43 ± 2	26 ± 1	43 ± 1	27 ± 1	47 ± 2
**5-15**	30 ± 2	15 ± 1	23 ± 2	15 ± 1	30 ± 1	34 ± 2	15 ± 1	24 ± 2	31 ± 1	43 ± 2	24 ± 2	30 ± 1	17 ± 1	39 ± 2
**5-16**	50 ± 2	35 ± 1	45 ± 1	47 ± 1	67 ± 1	53 ± 1	52 ± 1	39 ± 1	13 ± 1	66 ± 2	35 ± 1	43 ± 2	39 ± 1	54 ± 2
**5-17**	57 ± 2	47 ± 2	81 ± 2	24 ± 2	86 ± 2	34 ± 1	72 ± 2	27 ± 1	27 ± 1	62 ± 2	35 ± 2	20 ± 1	14 ± 1	34 ± 2
**5-18**	69 ± 2	52 ± 1	72 ± 2	45 ± 2	64 ± 2	44 ± 1	75 ± 2	37 ± 1	52 ± 2	62 ± 2	45 ± 2	27 ± 2	37 ± 1	45 ± 2
**5-19**	33 ± 1	10 ± 1	27 ± 1	13 ± 1	50 ± 1	18 ± 1	25 ± 1	22 ± 2	27 ± 1	73 ± 1	49 ± 2	22 ± 1	28 ± 2	42 ± 1
chlorothalonil	38 ± 1	98 ± 1	77 ± 1	8 ± 1	100	35 ± 1	100	97 ± 1	30 ± 2	98 ± 1	99 ± 1	95 ± 2	95 ± 2	92 ± 2

*A.S.*, *Alternaria solani*; *F.G.*, *Fusarium graminearum*; *P.G.*, *Pyricularia grisea*; *P.C.*, *Phytophthora capsica*; *S.S.*, *Sclerotinia sclerotiorum*; *B.C.*, *Botrytis cinereapers*; *R.S.*, *Rhizoctonia solani*; *F.C.*, *Fusarium oxysporium* f. sp. *Cucumeris*; *C.H.*, *Cercospora arachidicola Hori*; *P.P.*, *Physalospora piricola*; *R.C.*, *Rhizoctonia cereadis Vander Hoeven*; *B.M.*, *Bipolaris maydis*; *W.A.*, *Watermelon-anthracnose*; *F.M.*, *Fusarium moniliforme*.

## Data Availability

Data have been stored in the library repository of Nankai University, Tianjin, China.

## Supplementary Materials

The following supporting information can be downloaded at: https://www.mdpi.com/article/10.3390/ijms26178308/s1.
