# Supplementary material for: Design, Synthesis, and Biological Activity Studies of Aldisine Derivatives Containing Acylhydrazone Moiety
Source: Int J Mol Sci. 2025 Aug 27;26(17):8308. doi: 10.3390/ijms26178308 (PMC12427789; doi:10.3390/ijms26178308)
Supplement: Supplementary file 1 [file ijms-26-08308-s001.zip › ijms-3823875-supplementary.pdf]

Design, Synthesis, and Biological Activity Studies of Aldisine Derivatives Containing Acylhydrazone Moiety

Wentao Xu,[1] Kangkang Yang,[1] Mingxing Li,[1] Longqi Li,[1] Fuqiao Xing,[1] Jiayi Li,[1] Yuxiu Liu,[1] Jingjing Zhang,[1,2*] Qingmin Wang[1*] and Hongjian Song[1*]

[1]State Key Laboratory of Elemento-Organic Chemistry, Frontiers Science Center for New Organic Matter, College of Chemistry, Nankai University, Tianjin 300071, China

[2] College of Basic Science, Tianjin Agricultural University, Tianjin 300384, China

Table of contents

1. The operating steps and physical data in detail of intermediates **1**, and target compounds **2,3,4,5**……S2-S14

2. Copies of NMR spectra……S15-S41

3. Detailed bioassay methods……S42-S45

4. Calculation procedures for molecular docking research……S45

1-benzyl-6,7-dihydropyrrolo[2,3-*c*]azepine-4,8(1*H*,5*H*)-dione (1)

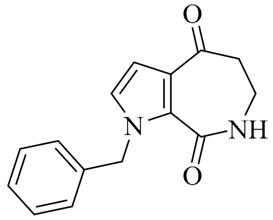

A mixture of aldisine (493 mg, 3.00 mmol), benzyl bromide (0.77 g, 4.50 mmol), and potassium carbonate (1.95 g, 6 mmol) in anhydrous acetonitrile (24 mL) was heated at reflux for 12 hours. When TLC indicated that the reaction was complete, the reaction mixture was cooled to room temperature, was concentrated in vacuo, ethyl acetate was added to dissolve the crude product, then washed by saturated salt water, dried with anhydrous magnesium sulfate, concentrated in vacuo, and then purified by chromatography on a column of silica gel (petroleum ether: ethyl acetate = 1:2) to offer **1** as a white solid, 533 mg, 70% yield, melting point:150–152 °C。^{1}H NMR (400 MHz, $CDCl_3$) δ 7.30 (dt, J = 11.9, 6.8 Hz, 3H), 7.15 (d, J = 7.2 Hz, 2H), 6.86 (d, J = 2.9 Hz, 1H), 6.76 (d, J = 2.9 Hz, 1H), 6.27 (s, 1H), 5.63 (s, 2H), 3.50 (q, J = 5.9 Hz, 2H), 2.85 – 2.80 (m, 2H). ^{13}C NMR (100 MHz, $CDCl_3$) δ 194.8, 163.5, 137.6, 128.8, 128.2, 127.8, 127.2, 127.0, 125.6, 109.8, 52.6, 44.1, 37.6. HRMS (ESI): calcd for $C_{15}H_{14}N_2O_2$ $[M+H]^+$ 255.1128, found 255.1127.

2-(1-benzyl-4,8-dioxo-4,5,6,8-tetrahydropyrrolo[2,3-*c*]azepin-7(1*H*)-yl)methyl acetate (2-1)

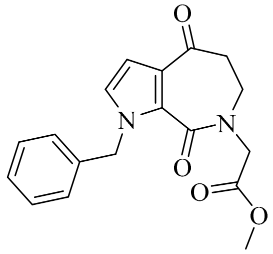

To a solution of **1** (1.02 g, 4.00 mmol) in anhydrous *N*, *N*-dimethylformamide (40 mL) was add sodium hydride (192 mg, 8 mmol, 60% of sodium hydride (in mineral oil)) under ice bath condition. After reaction for 1.5 hours, methyl bromoacetate (1.26 g, 8.00 mmol) was added, the reaction mixture warmed to room temperature and reacted for 12 hours. A small amount of ice water was added to quench the excess sodium hydride, then water was add and extracted with ethyl acetate (50 mL × 4), the combined organic phase was dried over anhydrous sodium sulfate, evaporated under reduced pressure, and then purified by silica column chromatography (eluent: petroleum ether : ethyl acetate =1:1) to give **2-1** as a white solid, 1.03 g, 79% yield, melting point: 96–98 °C.^{1}H NMR (400 MHz, $CDCl_3$) δ 7.31 (dt, J = 13.0, 6.8 Hz, 3H), 7.17 (d, J = 7.3 Hz, 2H), 6.85 (d, J = 2.8 Hz, 1H), 6.71 (d, J = 2.8 Hz, 1H), 5.57 (s, 2H), 4.35 (s, 2H), 3.74 (s, 5H), 3.02 – 2.88 (m, 2H). ^{13}C NMR (100 MHz, $CDCl_3$) δ 195.0, 169.6, 161.8, 137.5, 128.7, 128.0, 127.7, 127.3, 126.5, 126.2, 109.4, 52.6, 52.37, 50.1, 46.3, 42.7. HRMS (ESI): calcd for $C_{18}H_{18}N_2O_4$ $[M+H]^+$ 327.1339, found 327.1339.

2-(1-benzyl-4,8-dioxo-4,5,6,8-tetrahydropyrrolo[2,3-*c*]azepin-7(1*H*)-yl)ethyl acetate (2-2)

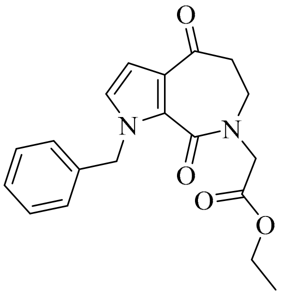

White solid, 72% yield, melting point: 73–74 °C。 ^{1}H NMR (400 MHz, $CDCl_3$) δ 7.33 (q, J = 6.8, 6.1 Hz, 3H), 7.18 (d, J = 7.3 Hz, 2H), 6.85 (d, J = 2.8 Hz, 1H), 6.72 (d, J = 2.5 Hz, 1H), 5.58 (s, 2H), 4.34 (s, 2H), 4.22 (q, J = 7.2 Hz, 2H), 3.75 (t, J = 5.6 Hz, 2H), 2.96 (t, J = 5.2 Hz, 2H), 1.28 (t, J = 7.2 Hz, 3H). ^{13}C NMR (100 MHz, $CDCl_3$) δ 194.5, 168.6, 161.2, 137.1, 128.2, 127.4, 127.2, 126.8, 125.8, 125.7, 108.7, 60.9, 51.9, 49.6, 45.7, 42.2, 13.6. HRMS (ESI): calcd for $C_{18}H_{18}N_2O_4$ $[M+H]^+$ 327.1339, found 327.1338.

2-(1-benzyl-4,8-dioxo-4,5,6,8-tetrahydropyrrolo[2,3-*c*]azepin-7(1*H*)-yl)butyl acetate (2-3)

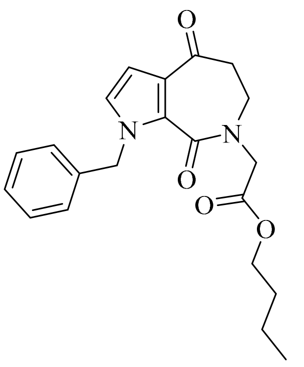

Yellow oil, 72% yield.^{1}H NMR (400 MHz, $CDCl_3$) δ 7.35 – 7.27 (m, 3H), 7.17 (d, J = 7.4 Hz, 2H), 6.84 (t, J = 2.5 Hz, 1H), 6.71 (t, J = 2.4 Hz, 1H), 5.59 (s, 2H), 4.28 – 4.22 (m, 2H), 3.74 (s, 2H), 2.96 (t, J = 4.8 Hz, 2H), 1.48 (s, 9H). ^{13}C NMR (100 MHz, $CDCl_3$) δ 195.1, 168.2, 161.7, 137.6, 128.7, 127.8, 127.7, 127.3, 126.4, 126.4, 109.3, 82.2, 52.6, 51.0, 46.3, 42.8, 28.1, 28.0, 27.9. HRMS (ESI): calcd for $C_{21}H_{24}N_2O_4$ $[M+Na]^+$ 391.1628, found 391.1629.

2-(1-benzyl-4,8-dioxo-4,5,6,8-tetrahydropyrrolo[2,3-*c*]azepin-7(1*H*)-yl)benzyl acetate (2-4)

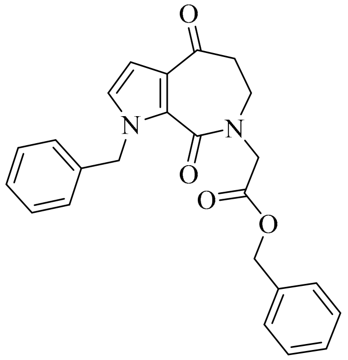

Yellow oil, 71% yield. ^{1}H NMR (400 MHz, $CDCl_3$) δ 7.34 (s, 4H), 7.28 (dt, J = 11.9, 6.7 Hz, 4H), 7.17 – 7.12 (m, 2H), 6.82 (d, J = 2.9 Hz, 1H), 6.69 (d, J = 2.9 Hz, 1H), 5.54 (s, 2H), 5.16 (s, 2H), 4.36 (s, 2H), 3.75 – 3.65 (m, 2H), 2.93 – 2.86 (m, 2H). ^{13}C NMR (100 MHz, $CDCl_3$) δ 194.9, 169.0, 161.8, 137.5, 135.2, 128.8, 128.7, 128.5, 128.4, 128.0, 127.8, 127.4, 126.5, 126.2, 109.4, 67.2, 52.6, 50.2, 46.3, 42.7. HRMS (ESI): calcd for $C_{24}H_{22}N_2O_4$ $[M+H]^+$ 403.1652, found 403.1652.

2-(1-benzyl-4,8-dioxo-4,5,6,8-tetrahydropyrrolo[2,3-*c*]azepin-7(1*H*)-yl)*tert*-butyl

acetate (2-5)

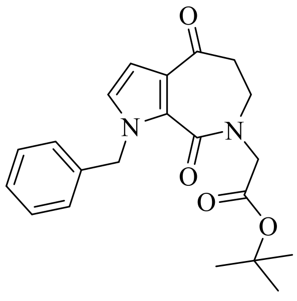

Yellow solid, 82% yield, melting point: 85–87 °C。 ^{1}H NMR (400 MHz, $CDCl_3$) δ 7.33 – 7.29 (m, 2H), 7.27 – 7.24 (m, 1H), 7.18 – 7.13 (m, 2H), 6.82 (d, J = 2.9 Hz, 1H), 6.69 (d, J = 2.9 Hz, 1H), 5.57 (s, 2H), 4.23 (s, 2H), 3.72 (t, J = 5.5 Hz, 2H), 2.97 – 2.90 (m, 2H), 1.45 (s, 9H). ^{13}C NMR (101 MHz, $CDCl_3$) δ 195.1, 168.2, 161.7, 137.6, 128.7, 127.8, 127.7, 127.4, 126.4, 126.4, 109.3, 82.2, 52.6, 51.0, 46.3, 42.8, 28.1. HRMS (ESI): calcd for $C_{21}H_{24}N_2O_4$ [M+Na]$^+$ 391.1628, found 391.1630.

2-(1-benzyl-4,8-dioxo-4,5,6,8-tetrahydropyrrolo[2,3-*c*]azepin-7(1*H*)-yl)ethyl butanoate (2-6)

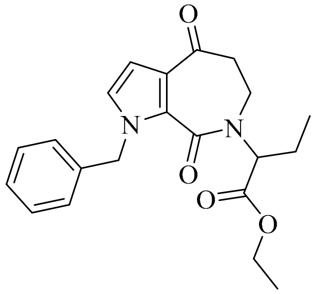

Yellow oil, 67% yield. ^{1}H NMR (400 MHz, $CDCl_3$) δ 7.31 – 7.28 (m, 1H), 7.27 (s, 1H), 7.26 – 7.21 (m, 1H), 7.11 (d, J = 6.8 Hz, 2H), 6.89 (d, J = 2.9 Hz, 1H), 6.71 (d, J = 2.9 Hz, 1H), 5.64 (d, J = 15.1 Hz, 1H), 5.52 (d, J = 15.1 Hz, 1H), 5.18 (dd, J = 10.6, 5.3 Hz, 1H), 4.15 (q, J = 7.1 Hz, 2H), 3.58 (dt, J = 23.7, 12.0 Hz, 2H), 3.00 (dd, J = 18.7, 8.3 Hz, 1H), 2.80 (dd, J = 18.8, 10.0 Hz, 1H), 2.12 – 1.99 (m, 1H), 1.71 (dq, J = 7.4, 4.3 Hz, 1H), 1.23 (t, J = 7.1 Hz, 3H), 0.77 (t, J = 7.4 Hz, 3H). ^{13}C NMR (100 MHz, $CDCl_3$) δ 195.3, 171.2, 162.0, 137.7, 128.6, 128.2, 127.7, 127.1, 126.7, 126.4, 108.9, 61.3, 58.6, 52.9, 43.3, 41.4, 22.6, 14.2, 10.6. HRMS (ESI): calcd for $C_{21}H_{24}N_2O_4$ [M+H]$^+$ 369.1809, found 369.1812.

Ethyl 2-(1-benzyl-4-hydroxy-8-oxo-4,5,6,8-tetrahydropyrrolo[2,3-*c*]azepin-7(1*H*)-yl)acetate (3)

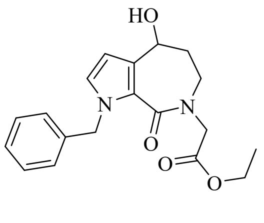

To a solution of **2-2** (3.40 g, 10 mmol) in anhydrous methanol (50 mL) was added sodium borohydride (567 mg, 15 mmol, 60% of sodium hydride (in mineral oil)) under ice bath conditions. After 30 minutes of reaction, the ice bath was removed and the reaction continued for 12 hours. When TLC indicated that the reaction was complete, a small amount of ice water was added to quench the excess sodium borohydride, then water was add and extracted with ethyl acetate, wash with brine, dry with anhydrous magnesium sulfate, evaporated under reduced pressure, and then purified by silica column chromatography (eluent:

petroleum ether : acetate =1:1) to obtain a white solid 3.10 g, 90% yield, melting point: 98–100 °C。 ^{1}H NMR (400 MHz, $CDCl_3$) δ 7.29 (dd, J = 8.1, 6.2 Hz, 2H), 7.26 – 7.20 (m, 1H), 7.20 – 7.05 (m, 2H), 6.79 (d, J = 2.7 Hz, 1H), 6.21 (d, J = 2.7 Hz, 1H), 5.60 – 5.41 (m, 2H), 4.96 (dd, J = 6.7, 3.9 Hz, 1H), 4.42 (d, J = 17.4 Hz, 1H), 4.19 (q, J = 7.1 Hz, 2H), 4.06 (d, J = 17.4 Hz, 1H), 3.45 (dd, J = 6.7, 5.0 Hz, 2H), 2.96 (s, 1H), 2.45 – 2.32 (m, 1H), 2.31 – 2.18 (m, 1H), 1.26 (t, J = 7.2 Hz, 3H). ^{13}C NMR (100 MHz, $CDCl_3$) δ 170.3, 164.2, 138.6, 131.8, 128.6, 127.4, 127.1, 126.9, 121.0, 107.8, 66.5, 61.5, 51.9, 50.0, 47.5, 38.0, 14.1. HRMS (ESI): calcd for $C_{19}H_{22}N_2O_4Na$ $[M+Na]^+$ 365.1472, found 365.1473.

2-(1-benzyl-4-hydroxy-8-oxo-4,5,6,8-tetrahydropyrrolo[2,3-c]azepin-7(1H)-yl)acetohydrazide (4)

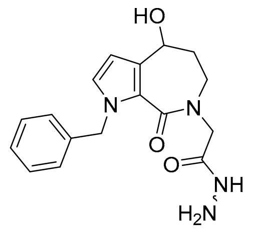

T a solution of **3** (610 mg, 1.78 mmol) in anhydrous ethanol (18 mL) was add hydrazine hydrate (1.75 mL, 35.60 mmol), then the mixture was heated at reflux for 6 hours. When TLC indicated that the reaction was complete, the reaction mixture was cooled to room temperature, concentrated in vacuo to offer a light-yellow solid, 584 mg, quantitative yield, melting point: 127–129 °C. ^{1}H NMR (400 MHz, DMSO-d_6) δ 9.23 (s, 1H), 7.31 – 7.20 (m, 3H), 7.11 (d, J = 7.5 Hz, 2H), 7.02 (d, J = 2.7 Hz, 1H), 6.22 – 6.10 (m, 1H), 5.52 – 5.30 (m, 3H), 4.77 (dd, J = 7.1, 3.3 Hz, 1H), 4.25 (s, 2H), 4.09 (dd, J = 16.0, 3.3 Hz, 1H), 3.95 (d, J = 16.1 Hz, 1H), 3.52 – 3.36 (m, 1H), 3.33 – 3.24 (m, 1H), 2.26 (dtd, J = 14.5, 7.3, 3.8 Hz, 1H), 1.94 (dd, J = 13.1, 7.7 Hz, 1H). ^{13}C NMR (100 MHz, DMSO-d_6) δ 168.2, 163.6, 139.4, 131.6, 128.3, 127.0, 126.9, 126.1, 120.8, 107.7, 65.0, 50.6, 49.4, 46.9, 38.0. HRMS (ESI): calcd for $C_{17}H_{20}N_4O_3Na$ $[M+Na]^+$ 351.1428, found 351.1426.

2-(1-benzyl-4-hydroxy-8-oxo-4,5,6,8-tetrahydropyrrolo[2,3-*c*]azepin-7(1*H*)-yl)-*N'*-benzylideneacetohydrazide (5-1)

To a solution of **4** (328 mg, 1.00 mmol) in anhydrous ethanol (20 mL), then benzaldehyde (159 mg, 1.5 mmol) was added, and reacted at room temperature for 4 hours. When TLC indicated that the reaction was complete, evaporated under reduced pressure, and then purified by silica column chromatography (eluent: ethyl acetate) to give a white solid, 379 mg, 91% yield, melting point: 103–104 °C. ^{1}H NMR (400 MHz, $CDCl_3$) δ 11.04 and 10.18 (s, 1H), 8.02 and 7.79 (s, 1H), 7.72 – 7.65 (m, 1H), 7.62 – 7.56 (m, 1H), 7.39 – 7.27 (m, 4H), 7.24 – 7.18 (m, 1H), 7.17 – 6.93 (m, 2H), 6.80 and

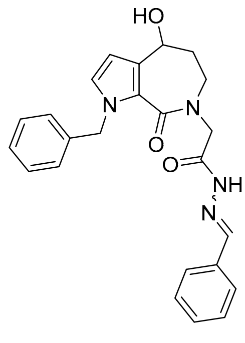

6.77 (d, J = 2.6 Hz, 1H), 6.20 and 6.16 (d, J = 2.7 Hz, 1H), 5.68 – 5.54 (m, 1H), 5.37 – 5.24 (m, 1H), 5.20 – 4.94 (m, 1H), 4.94 – 4.58 (m, 1H), 4.27 – 4.07 (m) and 3.81 – 3.67 (m, 1H), 3.67 – 3.56 (m, 1H), 3.33 – 3.21 (m, 1H), 2.55 – 2.10 (m, 2H). ^{13}C NMR (100 MHz, DMSO-d_6) δ 171.4 and 166.7, 165.1 and 164.8, 147.8 and 144.4, 140.7 and 40.5, 135.4 and 135.3, 133.2 and 133.1, 131.2 and 131.0, 129.9, 129.5 and 129.4, 128.2, 128.2 and 128.2, 128.1 and 127.9, 127.6 and 127.5, 121.9 and 121.5, 109.0 and 108.8, 66.3 and 66.2, 56.1, 51.9 and 50.8, 48.5 and 47.6, 39.2. HRMS (ESI): calcd for $C_{24}H_{24}N_4O_3Na$ [M+Na]$^+$ 439.1741, found 439.1744.

2-(1-benzyl-4-hydroxy-8-oxo-4,5,6,8-tetrahydropyrrolo[2,3-*c*]azepin-7(1*H*)-yl)-*N′*-(4-methylbenzylidene)acetohydrazide (5-2)

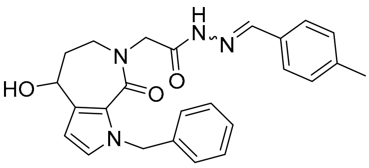

White solid, 89% yield, melting point: 200–202 °C。^{1}H NMR (400 MHz, DMSO-d_6) δ 11.54 and 11.43 (s, 1H), 8.12 and 7.95 (s, 1H), 7.63 – 7.54 (m, 2H), 7.31 – 7.20 (m, 5H), 7.17 – 7.10 (m, 2H), 7.06 and 7.04 (d, J = 2.7 Hz, 2H), 6.19 and 6.17 (d, J = 2.6 Hz, 1H), 5.59 – 5.30 and 5.18 – 5.13 (m, 1H), 4.93 – 4.74 (m, 1H), 4.72 – 4.49 and 4.26 – 3.08 (m, 2H), 3.55 – 3.34 and 3.33 – 3.25 (m, 2H), 2.45 – 2.36 and 2.31 – 2.24 (m) and 2.07 – 1.83 (m, 2H), 2.33 (s, 3H). ^{13}C NMR (100 MHz, DMSO-d_6) δ 170.6 and 165.9, 164.4 and 164.1, 147.2 and 143.7, 140.3 and 139.8, 140.0, 132.5 and 132.4, 132.0 and 131.9, 129.8, 128.83 and 128.78, 127.54 and 127.51, 127.4 and 127.2, 126.8, 121.2 and 120.8, 108.3 and 108.1, 65.6 and 65.5, 51.1, 50.1, 47.8 and 47.0, 38.5, 21.5. HRMS (ESI): calcd for $C_{25}H_{25}N_4O_3$ [M-H]$^-$ 429.1932, found 429.1923.

2-(1-benzyl-4-hydroxy-8-oxo-4,5,6,8-tetrahydropyrrolo[2,3-*c*]azepin-7(1*H*)-yl)-*N′*-(4-(tert-butyl)benzylidene)acetohydrazide (5-3)

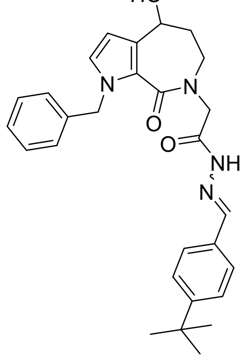

White solid, 92% yield, melting point: 109–111 °C。^{1}H NMR (400 MHz, $CDCl_3$) δ 11.04 (s) and 10.31 (s, 1H), 8.00 and 7.81 (s, 1H), 7.60 and 7.53 (d, J = 8.1 Hz, 2H), 7.40 – 7.30 (m, 2H), 7.26 – 7.17 (m, 2H), 7.10 and 7.06 (d, J = 7.3 Hz, 2H), 6.67 – 6.84 (m, 1H), 6.19 and 6.14 (s, 1H), 5.62 and 5.59 (d, J = 5.7 Hz, 1H), 5.35 – 5.21 and 4.25 – 4.15 (m, 2H), 5.10 – 4.74 (m, 2H), 3.87 – 3.50 (m, 2H), 3.38 – 3.10 (m, 1H), 2.44 – 2.08 (m,

2H), 1.31 and 1.28 (s, 9H). ^{13}C NMR (100 MHz, DMSO-d_6) δ 170.6 and 165.9, 164.4 and 164.1, 153.3 and 153.0, 147.1 and 143.7, 140.0 and 139.8, 132.5 and 132.4, 132.0 and 131.9, 128.8 and 128.8, 127.6 and 127.5, 127.4 and 127.3, 127.1, 126.8 and 126.8, 126.1, 121.2 and 120.8, 108.3 and 108.1, 65.6 and 65.5, 51.1, 50.1, 47.8 and 47.0, 38.5, 35.1 and 35.0, 31.4. HRMS (ESI): calcd for $C_{28}H_{32}N_4O_3Na$ [M+Na]$^+$ 495.2367, found 495.2365.

2-(1-benzyl-4-hydroxy-8-oxo-4,5,6,8-tetrahydropyrrolo[2,3-*c*]azepin-7(1*H*)-yl)-*N'*-(4-(trifluoromethyl)benzylidene)acetohydrazide (5-4)

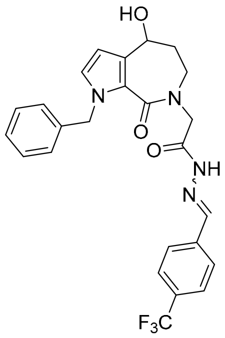

White solid, 87% yield, melting point: 160–161 °C。^{1}H NMR (400 MHz, DMSO-d_6) δ 11.80 and 11.69 (s, 1H), 8.23 and 8.06 (s, 1H), 7.96 – 7.75 (m, 4H), 7.32 – 7.25 (m, 2H), 7.24 – 7.19 (m, 1H), 7.18 – 7.10 (m, 2H), 7.06 and 7.05 (d, J = 2.7 Hz, 1H), 6.19 and 6.18 (d, J = 2.6 Hz, 1H), 5.63 – 5.28 and 5.16 – 5.12 (m, 3H), 4.87 – 4.77 (m, 1H), 4.73 – 4.49 (m) and 4.30 – 4.12 (m, 2H), 3.48 – 3.25 (m, 2H), 2.42 – 2.26 and 2.07 – 1.81 (m, 2H). ^{13}C NMR (100 MHz, DMSO-d_6) δ 171.0 and 166.3, 164.3 and 164.1, 145.3 and 142.0, 140.0 and 139.8, 138.7 and 138.5, 132.5 and 132.4, 130.1 and, 129.7, 128.9 and 128.8, 128.0 and 127.8, 127.5 and 127.4, 126.9 and 126.8, 126.1 and 126.0, 125.9 and 123.2, 121.1 and 120.7, 108.3 and 108.1, 65.6 and 65.5, 51.2 and 51.1, 51.0 and 50.1, 47.7 and 46.9, 38.4. HRMS (ESI): calcd for $C_{25}H_{23}F_3N_4O_3Na$ [M+Na]$^+$ 507.1614, found 507.1613.

2-(1-benzyl-4-hydroxy-8-oxo-4,5,6,8-tetrahydropyrrolo[2,3-*c*]azepin-7(1*H*)-yl)-*N'*-(4-chlorobenzylidene)acetohydrazide (5-5)

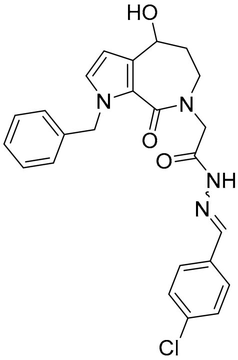

White solid, 88% yield, melting point: 208–210 °C。^{1}H NMR (400 MHz, DMSO-d_6) δ 11.67 and 11.56 (s, 1H), 8.15 and 7.98 (s, 1H), 7.75 – 7.66 (m, 2H), 7.54 – 7.46 (m, 2H), 7.32 – 7.26 (m, 2H), 7.25 – 7.19 (m, 1H), 7.17 – 7.09 (m, 2H), 7.06 (d, J = 2.6 Hz) and 7.04 (d, J = 2.6 Hz, 1H), 6.19 (d, J = 2.6 Hz) and 6.17 (d, J = 2.6 Hz, 1H). 5.60 – 5.28 and 5.16 – 5.11 (m, 3H), 4.87 – 4.75 (m, 1H), 4.70 – 4.54 and 4.25 – 4.10 (m, 2H), 3.54 – 3.35 and 3.33 – 3.25 (m, 2H), 2.42 – 2.25 and 1.85 – 1.81 (m, 1H). ^{13}C NMR (100 MHz, DMSO-d_6) δ 170.8 and 166.1, 164.4 and 164.1, 145.8 and 142.4, 140.0 and 139.8, 134.9 and 134.7, 133.6 and 133.5, 132.5 and 132.4, 129.4 and 129.3, 129.1 and 128.9, 128.83 and 128.78, 127.50 and127.48, 127.4, 126.9 and 126.8, 121.1 and 120.8, 108.3 and 108.1, 65.6 and

65.5, 51.1 and 50.1, 47.8, 46.9, 38.4. HRMS (ESI): calcd for $C_{24}H_{23}ClN_4O_3Na$ [M+Na]$^+$ 473.1351, found 473.1355.

2-(1-benzyl-4-hydroxy-8-oxo-4,5,6,8-tetrahydropyrrolo[2,3-*c*]azepin-7(1*H*)-yl)-*N'*-(3-chlorobenzylidene)acetohydrazide (5-6)

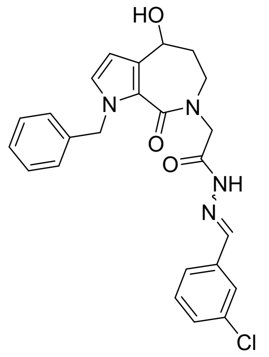

White solid, 85% yield, melting point: 103–105 °C。^{1}H NMR (400 MHz, $CDCl_3$) δ 11.12 and 10.35 (s, 1H), 7.96 and 7.74 (s, 1H), 7.67 and 7.60 (t, J = 1.5 Hz, 1H), 7.51 and 7.41 (d, J = 7.5 Hz, 1H), 7.35 – 7.27 (m, 3H), 7.25 – 7.19 (m, 2H), 7.15 – 6.96 (m, 2H), 6.80 and 6.79 (d, J = 2.6 Hz, 1H), 6.21 and 6.15 (d, J = 2.6 Hz, 1H), 5.64 and 5.60 (d, J = 2.7 Hz, 1H), 5.40 – 5.20 and 5.12 – 4.86 (m, 3H), 4.85 – 4.61 and 4.21 – 4.06 (m, 1H), 3.65 – 3.57 and 3.34 – 3.16 (m, 2H), 2.53 – 2.14 (m, 2H). ^{13}C NMR (100 MHz, $CDCl_3$) δ 172.3 and 166.7, 165.4 and 165.0, 147.3 and 144.6, 138.5 and 138.2, 135.7 and 135.5, 134.6 and 134.5, 132.8 and 131.6, 130.1 and 130.0, 129.8 and 129.7, 128.7 and 128.6, 127.6 and 127.4, 127.3 and 127.0, 126.9 and 126.64, 126.58 and 126.4, 126.00 and 125.97, 121.8 and 121.4, 108.2 and 108.1, 66.6 and 66.3, 51.7 and 51.6, 51.5 and 50.2, 49.2, 39.0 and 38.0. HRMS (ESI): calcd for $C_{24}H_{23}ClN_4O_3Na$ [M+Na]$^+$ 473.1351, found 473.1352.

2-(1-benzyl-4-hydroxy-8-oxo-4,5,6,8-tetrahydropyrrolo[2,3-*c*]azepin-7(1*H*)-yl)-*N'*-(2,4-dichlorobenzylidene)acetohydrazide (5-7)

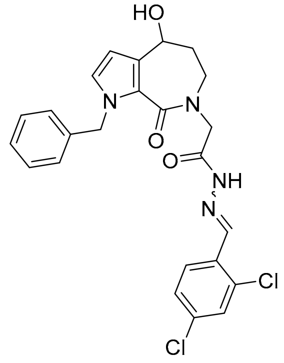

Yellow solid, 89% yield, melting point: 191–192 °C。^{1}H NMR (400 MHz, DMSO-d_6) δ 11.87 and 11.75 (s, 1H), 8.53 and 8.33 (s, 1H), 8.05 – 7.98 and 7.96 – 7.92 (m, 1H), 7.78 – 7.69 (m, 1H), 7.56 – 7.46 (m, 1H), 7.35 – 7.26 (m, 2H), 7.25 – 7.20 (m, 1H), 7.19 – 7.10 (m, 2H), 7.09 – 7.01 (m, 1H), 6.25 – 6.12 (m, 1H), 5.64 – 5.47 (m, 1H), 5.44 – 5.29 and 5.20 – 5.11 (m, 2H), 4.87 – 4.76 (m, 1H), 4.75 – 4.53 and 4.23 – 4.17 (m, 2H), 3.56 – 3.44 and 3.35 – 3.25 (m, 1H), 2.41 – 2.26 and 2.01 – 1.83 (m, 1H). ^{13}C NMR (100 MHz, DMSO-d_6) δ 170.4 and 165.8, 163.8 and 163.6, 141.4 and 138.1, 139.5 and 139.4, 135.0 and 134.8, 133.7 and 133.5, 132.1 and 132.0, 130.6 and 130.4, 129.3, 128.32 and 128.28, 128.0 and 127.9, 127.0 and 126.9, 126.5 and 126.4, 120.4 and 120.2, 107.8 and 107.6, 65.1, 50.6 and 50.5, 49.60, 47.0 and 46.40, 37.9 and 37.9. HRMS (ESI): calcd for $C_{24}H_{22}Cl_2N_4O_3Na$ [M+Na]$^+$ 507.0961, found 507.0962.

N'-([1,1'-biphenyl]-4-ylmethylene)-2-(1-benzyl-4-hydroxy-8-oxo-4,5,6,8-tetra-hydropyrrolo[2,3-*c*]azepin-7(1*H*)-yl)acetohydrazide (5-8)

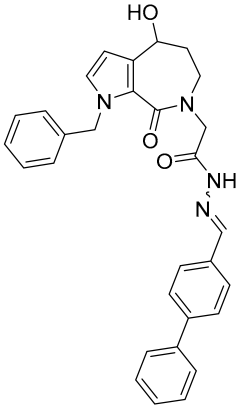

White solid, 64% yield, melting point: 176–178 ℃。^{1}H NMR (400 MHz, DMSO-d_6) δ 11.65 and 11.54 (s, 1H), 8.20 and 8.04 (s, 1H), 7.84 – 7.66 (m, 6H), 7.54 – 7.44 (m, 2H), 7.43 – 7.36 (m, 1H), 7.32 – 7.26 (m, 2H), 7.25 – 7.20 (m, 1H), 7.18 – 7.09 (m, 2H), 7.06 and 7.05 (d, J = 2.6 Hz, 1H), 6.19 and 6.18 (d, J = 2.6 Hz, 2H), 5.63 – 5.27 and 5.19 – 5.12 (m, 3H), 4.89 – 4.77 (m, 1H), 4.75 – 4.55 and 4.29 – 4.11 (m, 2H), 3.56 – 3.35 and 3.33 – 3.26 (m, 1H), 2.45 – 2.27 and 2.05 – 1.84 (m, 2H). ^{13}C NMR (100 MHz, DMSO-d_6) δ 170.2 and 165.6, 163.9 and163.6, 146.2 and 142.8, 141.5 and 141.3, 139.6 and 139.3, 133.3 and 133.21, 132.1 and 131.9, 129.0, 128.4, 128.3, 127.8, 127.6, 127.4, 127.1, 127.0, 126.9, 126.6 and 126.3, 120.7and 120.4, 107.8 and 107.6, 65.13 and 65.07, 50.7, 49.6, 47.3 and 46.5, 38.0. HRMS (ESI): calcd for $C_{30}H_{28}N_4O_3Na$ $[M+Na]^+$ 515.2054, found 515.2056.

2-(1-benzyl-4-hydroxy-8-oxo-4,5,6,8-tetrahydropyrrolo[2,3-*c*]azepin-7(1*H*)-yl)-N'-(naphthalen-2-ylmethylene)acetohydrazide (5-9)

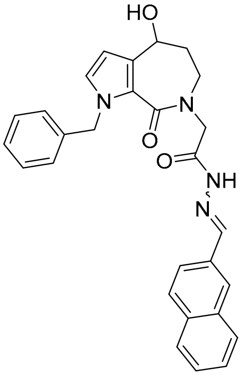

White solid, 87% yield, melting point: 212–213 ℃。^{1}H NMR (400 MHz, DMSO-d_6) δ 11.71 and 11.59 (s, 1H), 8.35 – 8.28 and 8.18 – 8.08 (m, 2H), 8.03 – 7.89 (m, 4H), 7.60 – 7.53 (m, 2H), 7.34 – 7.26 (m, 2H), 7.25 – 7.20 (m, 1H), 7.18 – 7.10 (m, 2H), 7.07 and 7.05 (d, J = 2.6 Hz, 1H), 6.20 and 6.18 (d, J = 2.6 Hz, 1H), 5.61 – 5.30 and 5.19 – 5.14 (m, 3H), 4.89 – 4.78 (m, 1H), 4.77 – 4.59 and 4.30 – 4.13 (m, 2H), 3.55 – 3.21 (m, 2H), 2.45 – 2.28 and 2.07 – 1.82 (m, 2H). ^{13}C NMR (100 MHz, DMSO-d_6) δ 170.3 and 165.6, 163.9 and 163.6, 146.6 and 145.4, 143.3 and 141.9, 139.6 and 139.4, 133.7 and 133.6, 132.8, 132.1, 131.96 and 131.92, 131.8, 128.8, 128.6, 128.4 and 128.3, 127.7, 127.0, 126.9, 126.7, 126.4, 122.6 and 122.4, 120.7 and 120.3, 107.8 and 107.6, 65.1, 50.6, 49.7, 47.3 and 46.5, 38.0. HRMS (ESI): calcd for $C_{28}H_{26}N_4O_3Na$ $[M+Na]^+$ 489.1897, found 489.1899.

2-(1-benzyl-4-hydroxy-8-oxo-4,5,6,8-tetrahydropyrrolo[2,3-*c*]azepin-7(1*H*)-yl)-N'-(quinolin-8-ylmethylene)acetohydrazide (5-10)

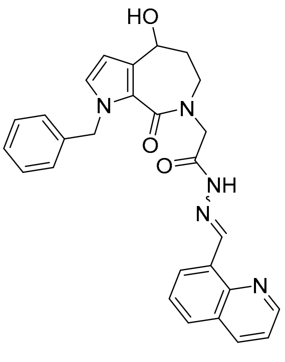

Yellow solid, 83% yield, melting point: 191–192 ℃。^{1}H NMR (400 MHz, DMSO-d_6) δ 11.81 and 11.72 (s, 1H), 9.49 and 9.29 (s, 1H), 9.05 – 8.95 (m, 1H), 8.46 and 8.44 (d, J = 2.0 Hz, 1H), 8.37 and 8.31 (dd, J = 7.4, 1.4 Hz, 1H), 8.12 – 8.05 (m, 1H), 7.72 and 7.68 (d, J = 7.8 Hz, 1H), 7.64 and 7.62 (d, J = 4.1 Hz, 1H). 7.33 – 7.10 (m, 5H), 7.09 – 7.03 (m, 1H), 6.23 – 6.15 (m, 1H), 5.63 – 5.48 (m, 1H), 5.43 – 5.29 and 5.20 – 5.13 (m, 2H), 4.88 – 4.62 and 4.22 (m, 3H), 3.56 – 3.35 and 3.33 – 3.25 (m, 2H), 2.47 – 2.29 and 1.98 – 1.82 (m, 2H). ^{13}C NMR (100 MHz, DMSO-d_6) δ 170.8 and 166.1, 164.20, and 164.15, 150.8, 145.7 and 144.1, 140.8, 140.0 and 139.9, 137.1, 132.5 and 132.4, 131.6 and 131.5, 130.4 and 130.2, 128.81 and 128.79, 128.5, 127.53 and 127.49, 127.4, 126.99 and 126.95, 126.8, 126.0 and 125.8, 122.3, 121.0 and 120.8, 108.3 and 108.1, 65.61 and 65.58, 51.1 and 50.9, 50.24, 47.4 and 47.0, 38.5 and 38.4. HRMS (ESI): calcd for $C_{27}H_{26}N_5O_3$ [M+H]$^+$ 490.1850, found 490.1847.

***N′*-((1*H*-indol-7-yl)methylene)-2-(1-benzyl-4-hydroxy-8-oxo-4,5,6,8-tetrahydro-pyrrolo[2,3-*c*]azepin-7(1*H*)-yl)acetohydrazide (5-11)**

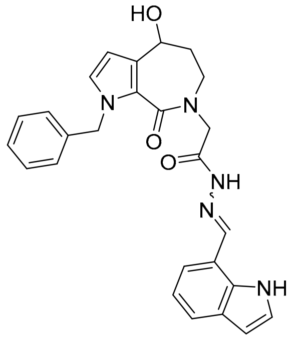

Yellow solid, 59% yield, melting point: 111–113 ℃。^{1}H NMR (400 MHz, $CDCl_3$) δ 11.12 and 10.56 (s, 1H), 8.19 and 7.98 (s, 1H), 7.75 – 7.66 (m, 1H), 7.29 – 7.27 and 7.25 – 7.19 (m, 2H), 7.15 – 7.02 (m, 4H), 6.81 and 6.78 (d, J = 2.7 Hz, 1H), 6.63 – 6.45 (m, 1H), 6.21 and 6.16 (d, J = 2.6 Hz, 1H), 5.71 – 5.56 and 5.42 – 5.28 (m, 1H), 5.19 – 4.99 and 4.93 – 4.88 (s, 2H), 3.73 – 3.55 and 3.29 – 3.14 (m, 3H), 2.57 – 2.44 and 2.41 – 2.28 and 2.22 – 2.10 (m, 2H). ^{13}C NMR (100 MHz, $CDCl_3$) δ 171.0 and 166.4, 165.5 and 164.8, 145.0 and 147.7, 138.6 and 138.2, 132.53 and 132.50, 132.4, 131.6, 128.7 and 128.6, 128.53 and 128.47, 127.7 and 127.4, 126.9 and 126.6, 125.8, 125.0, 123.6, 121.8 and 121.3, 119.4 and 119.0, 117.2 and 116.7, 108.2, 102.5 and 102.0, 66.5 and 66.3, 51.7, 51.5, 49.3 and 48.4, 38.6 and 38.0. HRMS (ESI): calcd for $C_{26}H_{25}N_5O_3Na$ [M+Na]$^+$ 478.1850, found 478.1853.

2-(1-benzyl-4-hydroxy-8-oxo-4,5,6,8-tetrahydropyrrolo[2,3-c]azepin-7(1H)-yl)-*N′*-(pyridin-3-ylmethylene)acetohydrazide (5-12)

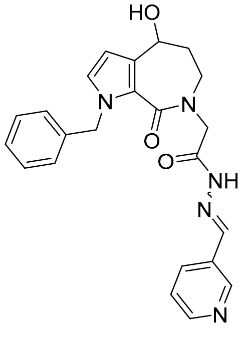

White solid, 89% yield, melting point: 108–110 °C。 ^{1}H NMR (400 MHz, $CDCl_3$) δ 11.56 and 10.42 (s, 1H), 8.76 – 8.70 and 8.38 (m, 1H), 8.60 – 8.54 (m, 1H), 8.22 and 7.94 (dt, J = 7.9, 1.9 Hz, 1H), 8.09 and 7.82 (s, 1H), 7.33 – 7.25 and 7.27 – 7.18 (m, 3H), 7.14 – 7.03 (m, 2H), 6.82 – 6.77 (m, 1H), 6.21 and 6.16 (d, J = 2.6 Hz, 1H), 5.63 and 5.59 (s, 1H), 5.34 – 5.22 and 5.17 – 5.08 and 4.91 (m, 3H), 4.25 – 4.14 and 3.62 – 3.54 (m, 1H), 3.80 – 3.64 (m, 1H), 3.35 – 3.21 (m, 1H), 2.61 – 2.49 and 2.44 – 2.31 and 2.23 – 2.15 (m, 2H). ^{13}C NMR (100 MHz, DMSO-d_6) δ 170.4 and 165.7, 163.8 and 163.6, 150.6 and 150.4, 148.6 and 148.4, 143.9, 140.4, 139.6 and 139.4, 133.34 and 133.26, 132.0 and 131.9, 130.1 and 130.0, 128.33 and 128.29, 127.0 and 126.9, 126.42 and 126.35, 124.0 and 123.9, 120.6 and 120.3, 107.8 and 107.6, 65.1 and 65.0, 50.6, 49.6, 47.2 and 46.4, 38.0. HRMS (ESI): calcd for $C_{23}H_{23}N_5O_3Na$ $[M+Na]^+$ 440.1693, found 440.1695.

2-(1-benzyl-4-hydroxy-8-oxo-4,5,6,8-tetrahydropyrrolo[2,3-*c*]azepin-7(1*H*)-yl)-*N′*-(thiophen-2-ylmethylene)acetohydrazide (5-13)

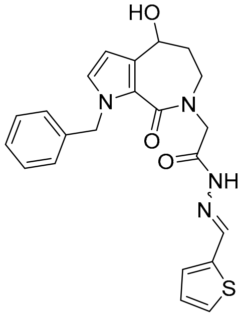

White solid, 71% yield, melting point: 106–108 °C. ^{1}H NMR (400 MHz, $CDCl_3$) δ 11.02 and 10.34 (s, 1H), 8.23 and 7.98 (s, 1H), 7.36 – 7.27 (m, 2H), 7.24 – 7.15 (m, 2H), 7.13 – 6.94 (m, 3H), 6.82 – 6.73 (m, 1H), 6.23 –6.11 (m, 1H), 5.63 and 5.59 (d, J = 5.6 Hz, 1H), 5.33 – 5.19 and 4.22 – 4.07 (m, 2H) 5.11 – 4.70 (m, 2H), 3.76 – 3.39 (m, 2H), 3.33 – 3.16 (m, 1H), 2.52 – 2.23 and 2.19 – 2.06 (m, 1H). ^{13}C NMR (100 MHz, $CDCl_3$) δ 171.9 and 166.6, 165.4 and 165.0, 143.9 and 140.8, 138.6 and 138.4, 132.7 and 131.9, 130.9, 128.7 and 128.63, 128.57, 128.1, 127.5 and 127.4, 127.1 and 127.0, 126.95 and 126.89, 126.4 and 126.3, 121.8 and 121.5, 108.2, 66.3, 51.6, 51.2 and 50.0, 49.1and 49.0, 38.9 and 38.2. HRMS (ESI): calcd for $C_{22}H_{22}N_4O_3SNa$ $[M+Na]^+$ 445.1305 found 445.1308.

2-(1-benzyl-4-hydroxy-8-oxo-4,5,6,8-tetrahydropyrrolo[2,3-*c*]azepin-7(1*H*)-yl)-*N′*-butylideneacetohydrazide (5-14)

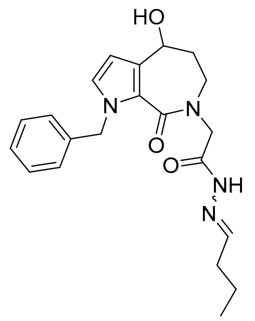

Yellow oil, 70% yield. ^{1}H NMR (400 MHz, $CDCl_3$) δ 10.67 and 9.51 (s, 1H), 7.37 – 7.28 (m, 2H), 7.26 – 7.21 (m, 1H), 7.18 – 7.04 (m, 2H), 6.79 and 6.75 (d, J = 2.6 Hz, 1H), 6.16 and 6.13 (d, J = 2.6 Hz, 1H), 5.63 and 5.59 (d, J = 5.6 Hz, 1H), 5.41 – 5.22 (m, 1H), 5.20 – 4.82 (m, 2H), 4.20 – 4.02 and 3.72 – 3.43 (m, 2H), 3.23 – 3.17 and 3.00 (m, 1H), 2.49 – 2.00 (m, 4H), 1.66 – 1.38 (m, 2H), 1.00 – 0.42 (m, 3H). ^{13}C NMR (100 MHz, $CDCl_3$) δ 171.6 and 166.3, 165.3 and 164.8, 153.4 and 150.1, 138.6 and 138.3, 132.5 and 131.8, 128.6 and 128.5, 127.5 and 127.3, 127.1 and 126.8, 126.4 and 126.0, 121.7 and 121.5, 108.10 and 108.06, 66.24 and 66.18, 51.5 and 51.0, 49.9, 49.0 and 48.90, 38.8 and 38.0, 34.4 and 34.2, 19.9 and 19.4, 13.8 and 13.74. HRMS (ESI): calcd for $C_{21}H_{26}N_4O_3Na$ $[M+Na]^+$ 405.1897, found 405.1898.

2-(1-benzyl-4-hydroxy-8-oxo-4,5,6,8-tetrahydropyrrolo[2,3-*c*]azepin-7(1*H*)-yl)-*N'*-octylideneacetohydrazide (5-15)

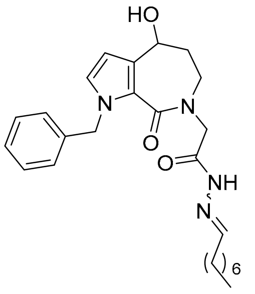

Yellow oil, 69% yield. ^{1}H NMR (400 MHz, $CDCl_3$) δ 10.66 and 9.57 (s, 1H), 7.37 – 7.28 and 7.25 – 7.14 (m, 3H), 7.11 and 7.07 (d, J = 7.3 Hz, 2H), 6.79 and 6.75 (d, J = 2.7 Hz, 1H), 6.16 and 6.14 (d, J = 2.7 Hz, 1H), 5.70 – 5.55 (m, 1H), 5.36 – 5.25 (m, 1H), 5.18 – 4.77 (m, 2H), 4.00 – 4.00 and 3.30 – 3.17 (m, 1H), 3.70 – 3.47 and 2.90 – 2.86 (m, 2H), 2.51 – 2.04 (m, 4H), 1.56 – 1.36 (m, 2H), 1.35 – 1.20 (m, 8H), 0.95 – 0.71 (m, 3H). ^{13}C NMR (100 MHz, $CDCl_3$) δ 171.6 and 166.3, 165.3 and 164.8, 153.6 and 150.3, 138.6 and 138.3, 132.6 and 131.8, 128.6 and 128.5, 127.5 and 127.4, 127.1 and 126.9, 126.4 and 126.0, 121.7 and 121.5, 108.1, 66.3 and 66.2, 51.5, 51.0 and 49.9, 49.0, 38.8 and 38.0, 32.6 and 32.3, 31.7, 29.3 and 29.2, 29.0, 26.5 and 26.0, 22.6, 14.1. HRMS (ESI): calcd for $C_{25}H_{34}N_4O_3Na$ $[M+Na]^+$ 461.2523, found 461.2520.

2-(1-benzyl-4-hydroxy-8-oxo-4,5,6,8-tetrahydropyrrolo[2,3-*c*]azepin-7(1*H*)-yl)-*N'*-(cyclopentylmethylene)acetohydrazide (5-16)

White solid, 72% yield, melting point: 81–83 °C。 ^{1}H NMR (400 MHz, $CDCl_3$) δ

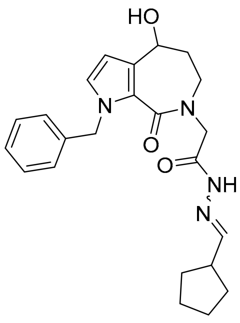

10.61 and 9.38 (s, 1H), 7.34 – 7.27 and 7.25 – 2.19 (m, 3H), 7.11 and 7.08 (d, J = 7.3 Hz, 2H), 6.79 and 6.76 (d, J = 2.7 Hz, 1H), 6.16 and 6.14 (d, J = 2.6 Hz, 1H), 5.63 and 5.59 (d, J = 5.0 Hz, 1H), 5.33 and 5.29 (s, H), 5.17 – 4.99 (m, 1H), 4.96 – 4.83 and 4.60 (m, 1H), 4.14 – 4.02 and 3.69 – 3.43 (m, 2H), 3.23 – 3.16 and 2.94 – 2.86 (s, 1H), 2.84 – 2.58 (m, 1H), 2.49 – 2.11 (m, 2H), 1.93 – 1.73 (m, 2H) 1.70 – 1.37 (m, 6H). ^{13}C NMR (100 MHz, $CDCl_3$) δ 171.5 and 166.0, 165.2 and 164.8, 156.7 and 153.1, 138.6 and 138.2, 132.5 and 131.3, 128.7 and 128.5, 127.6 and 127.4, 127.2 and 126.9, 126.5 and 126.1, 121.9 and 121.6, 108.0 and 107.9, 66.7 and 66.4, 51.7 and 51.6, 51.3 and 49.9, 48.9 and 48.8, 42.6 and 42.3, 38.8 and 37.7, 30.7, 30.3 and 30.2, 25.6 and 25.3. HRMS (ESI): calcd for $C_{23}H_{28}N_4O_3Na$ [M+Na]$^+$ 431.2054, found 431.2054.

2-(1-benzyl-4-hydrazono-8-oxo-4,5,6,8-tetrahydropyrrolo[2,3-*c*]azepin-7(1*H*)-yl)ethyl acetate (5-17)

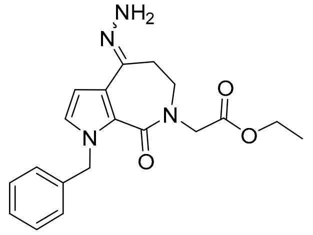

A solution of **2-2** (1.89 g, 5.55 mmol) and hydrazine hydrate (1.34 mL, 27.50 mmol, 80% of hydrazine in water) in anhydrous ethanol (50 mL) was heated at reflux for 6 hours. When TLC indicated that the reaction was complete, evaporated under reduced pressure, and then purified by silica column chromatography (eluent: petroleum ether : ethyl acetate=1:2) to give a yellow solid, 987 mg, yield 50%, melting point:127–128 °C。 ^{1}H NMR (400 MHz, $CDCl_3$) δ 7.31 – 7.23 (m, 3H), 7.13 (d, J = 6.8 Hz, 2H), 6.79 (d, J = 2.8 Hz, 1H), 6.47 (d, J = 2.8 Hz, 1H), 5.49 (s, 2H), 5.21 (s, 2H), 4.25 (s, 2H), 4.17 (q, J = 7.1 Hz, 2H), 3.67 (t, J = 5.3 Hz, 2H), 2.73 (dd, J = 6.4, 4.2 Hz, 2H), 1.24 (t, J = 7.1 Hz, 3H). ^{13}C NMR (100 MHz, $CDCl_3$) δ 169.6, 163.4, 146.3, 138.5, 128.6, 127.7, 127.4, 127.3, 127.2, 122.2, 106.6, 61.3, 52.0, 49.4, 46.5, 30.0, 14.2. HRMS (ESI): calcd for $C_{19}H_{22}N_4O_3$ [M+H]$^+$ 355.1765, found 355.1763.

2-(1-benzyl-4-(((*E*)-benzylidene)hydrazono)-8-oxo-4,5,6,8-tetrahydro-pyrrolo[2,3-*c*]azepin-7(1*H*)-yl)ethyl acetate (5-18)

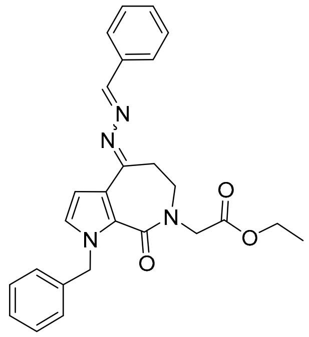

A solution of **2-2** (234 mg, 1 mmol) and benzaldehyde (112 μ L. 1.10 mmol) in anhydrous ethanol (25 mL) were reacted at room temperature for 4 hours. When TLC indicated that the reaction was complete, evaporated under reduced pressure, and then purified by silica column chromatography (eluent: petroleum ether : ethyl acetate = 2 : 1) to give a yellow solid, 343 mg, 81% yield, melting point : 117-118 °C. ^{1}H NMR (400 MHz, $CDCl_3$) δ 8.38 (s, 1H), 7.82 – 7.76 (m, 2H), 7.45 – 7.40 (m, 3H), 7.34 – 7.26 (m, 3H), 7.17 (d, J = 7.3 Hz, 2H), 6.86 (d, J = 2.7 Hz, 1H), 6.76 (d, J = 2.7 Hz, 1H), 5.55 (s, 2H), 4.27 (s, 2H), 4.18 (q, J = 7.1 Hz, 2H), 3.66 (dd, J = 6.9, 4.0 Hz, 2H), 3.30 (dd, J = 6.5, 4.3 Hz, 2H), 1.24 (t, J = 7.1 Hz, 3H). ^{13}C NMR (100 MHz, $CDCl_3$) δ 169.3, 163.1, 162.8, 157.3, 138.2, 134.7, 130.7, 128.7, 128.6, 128.2, 127.7, 127.46, 127.22, 125.85, 124.74, 107.68, 61.30, 52.3, 49.6, 46.8, 32.9, 14.1. HRMS (ESI): calcd for $C_{26}H_{27}N_4O_3$ [M+H]$^+$ 443.2078, found 443.2076.

(*E*)-2-(1-benzyl-4-(hydroxyimino)-8-oxo-4,5,6,8-tetrahydropyrrolo[2,3-*c*]azepin-7(1*H*)-yl)ethyl acetate (5-19)

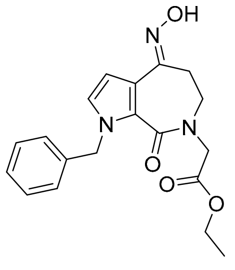

A mixture of **2-2** (255 mg, 0.75 mmol), hydroxylamine hydrochloride (104 mg, 1.50 mmol), and sodium acetate (123 mg, 1.50 mmol) in anhydrous methanol (10 mL), was heated at reflux for 5 hours. When TLC indicated that the reaction was complete, the reaction mixture was cooled to room temperature, concentrated in vacuo, then water was added. The mixture was extracted with ethyl acetate, dried with anhydrous magnesium sulfate, concentrated in vacuo to offer a white solid, 274 mg, 93% yield, melting point: 169–171 °C. ^{1}H NMR (400 MHz, DMSO-d_6) δ 11.03 (s, 1H), 7.29 (dd, J = 8.1, 6.4 Hz, 2H), 7.27 – 7.21 (m, 1H), 7.13 (dd, J = 6.9, 2.0 Hz, 3H), 6.32 (d, J = 2.7 Hz, 1H), 5.48 (s, 2H), 4.24 (s, 2H), 4.09 (q, J = 7.1 Hz, 2H), 3.52 (dd, J = 6.5, 4.4 Hz, 2H), 2.84 (dd, J = 6.6, 4.3 Hz, 2H), 1.16 (t, J = 7.1 Hz, 3H). ^{13}C NMR (100 MHz, DMSO-d_6) δ 169.9, 163.0, 152.7, 139.5, 128.9, 128.3, 127.7, 127.4, 124.4, 123.0, 106.5, 61.0, 51.2, 49.6, 46.6, 30.6, 14.5. HRMS (ESI): calcd for $C_{19}H_{22}N_3O_4$ [M+H]$^+$ 356.1605, found 356.1605.

Copies of NMR spectra (2, 3, 3, and 5)

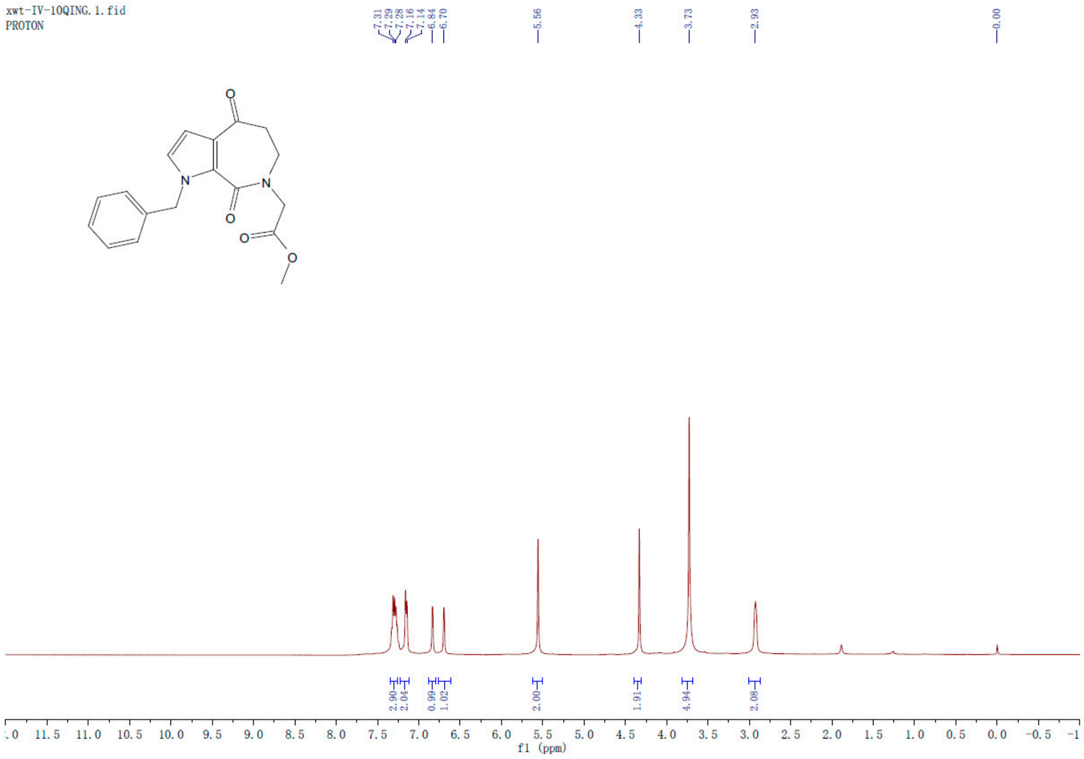

2-1 ^{1}H NMR (400 MHz, $CDCl_3$)

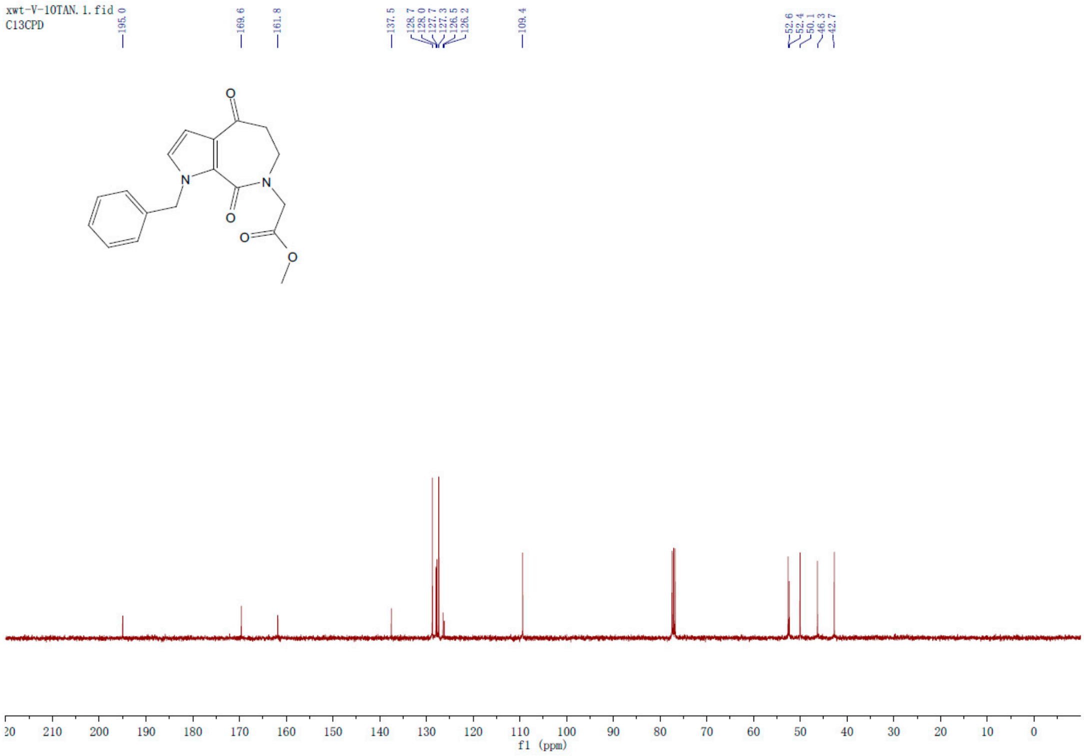

2-1 ^{13}C NMR (100 MHz, $CDCl_3$)

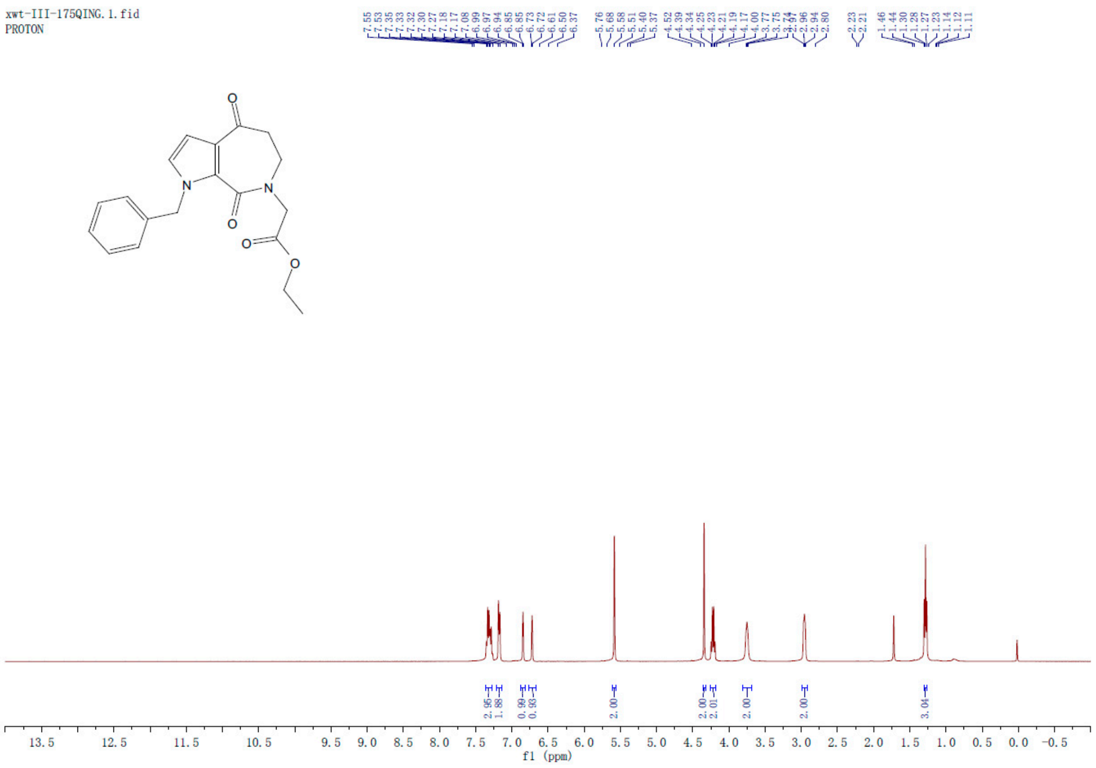

2-2 ^{1}H NMR (400 MHz, $CDCl_3$)

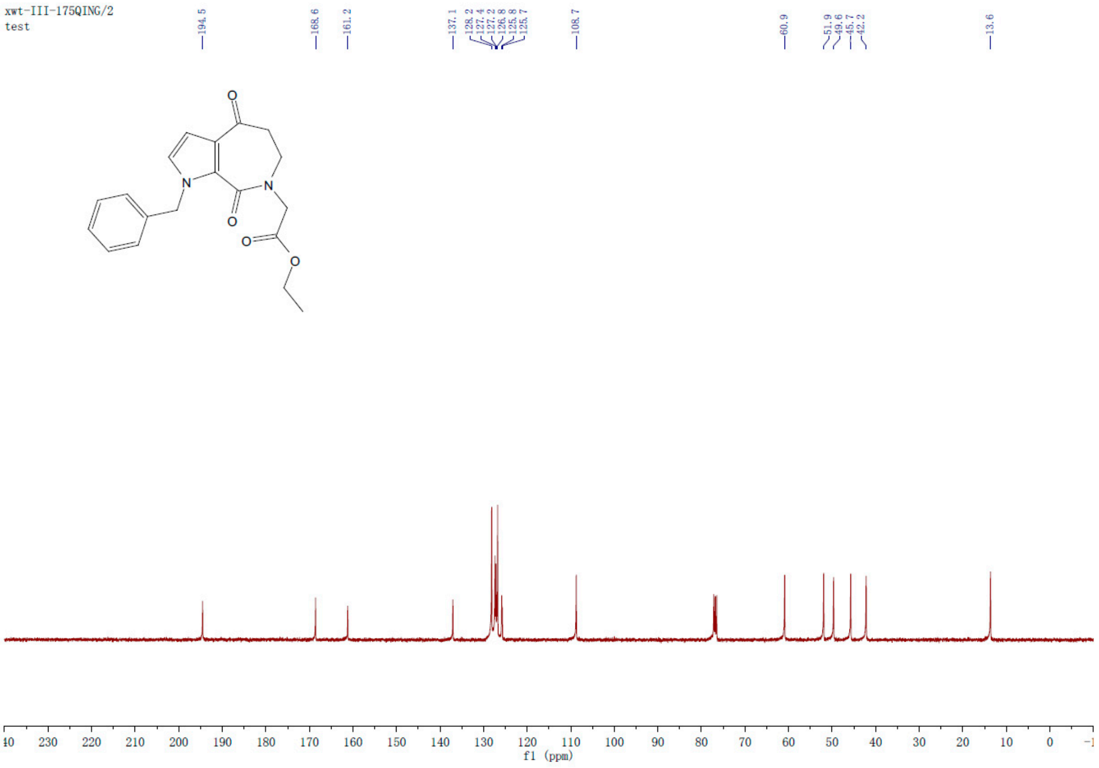

2-2 ^{13}C NMR (100 MHz, $CDCl_3$)

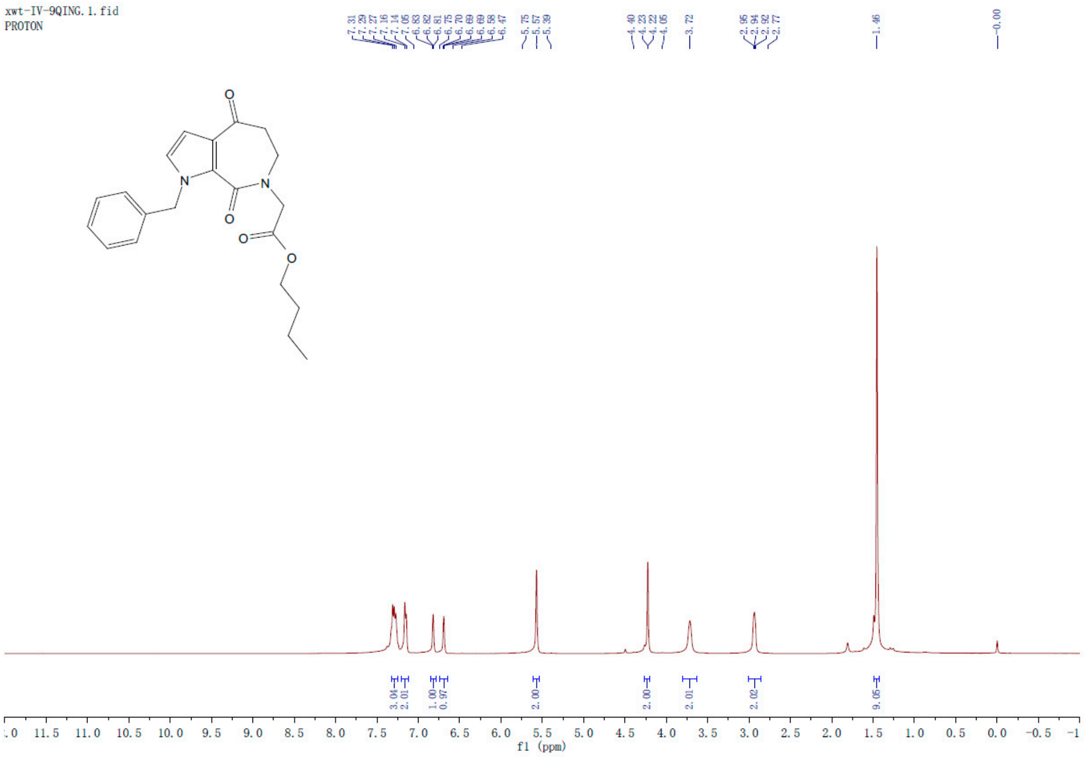

2-3 ^{1}H NMR (400 MHz, $CDCl_3$)

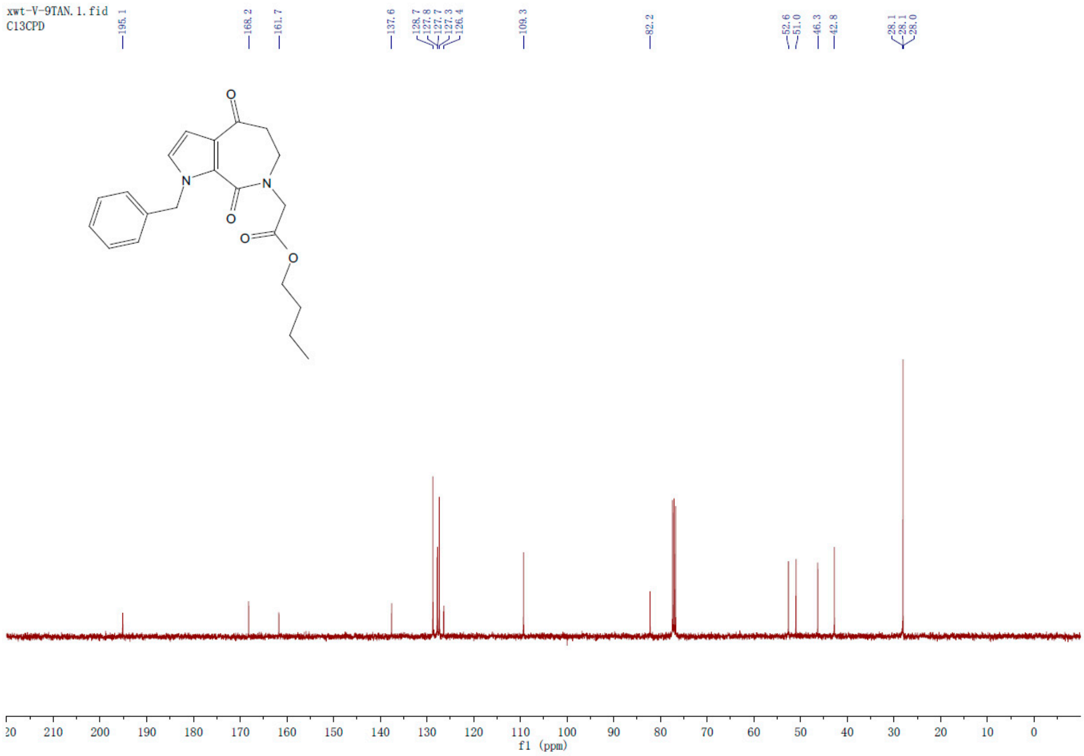

2-3 ^{13}C NMR (100 MHz, $CDCl_3$)

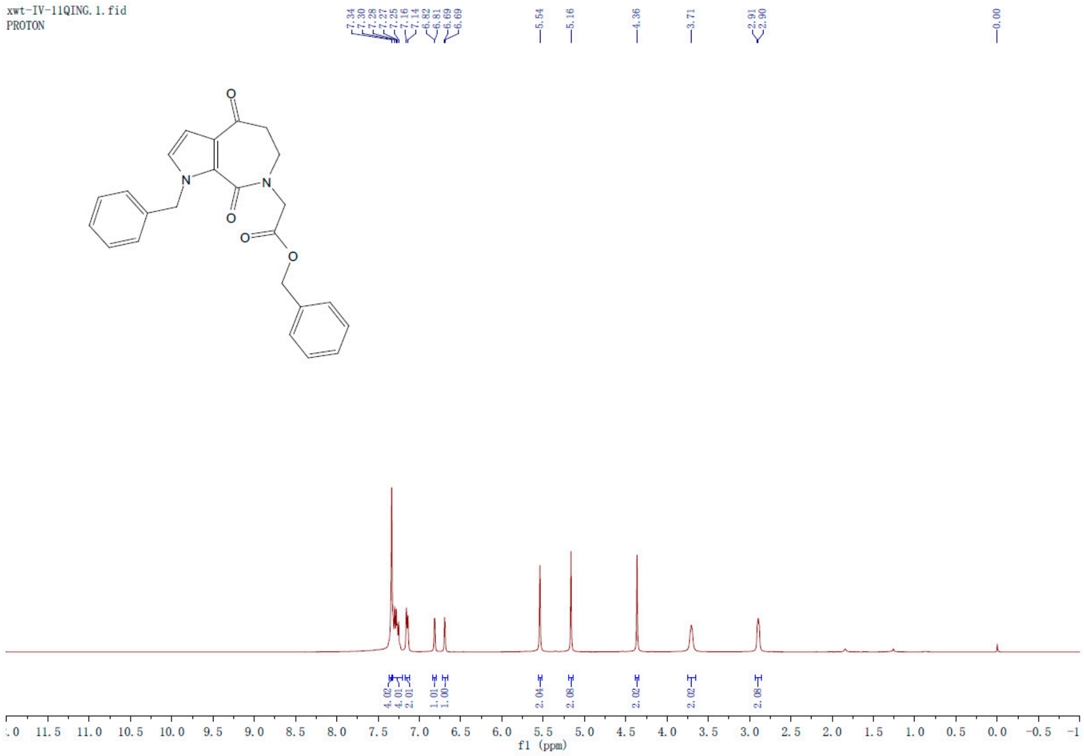

2-4 ^{1}H NMR (400 MHz, $CDCl_3$)

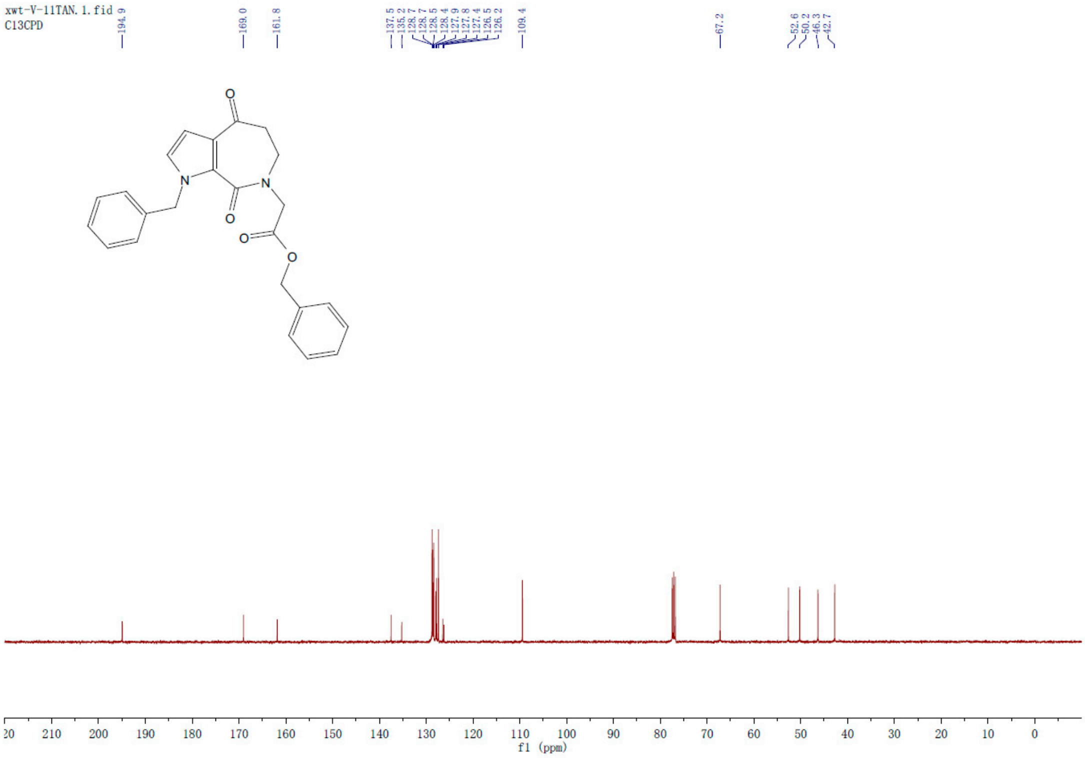

2-4 ^{13}C NMR (100 MHz, $CDCl_3$)

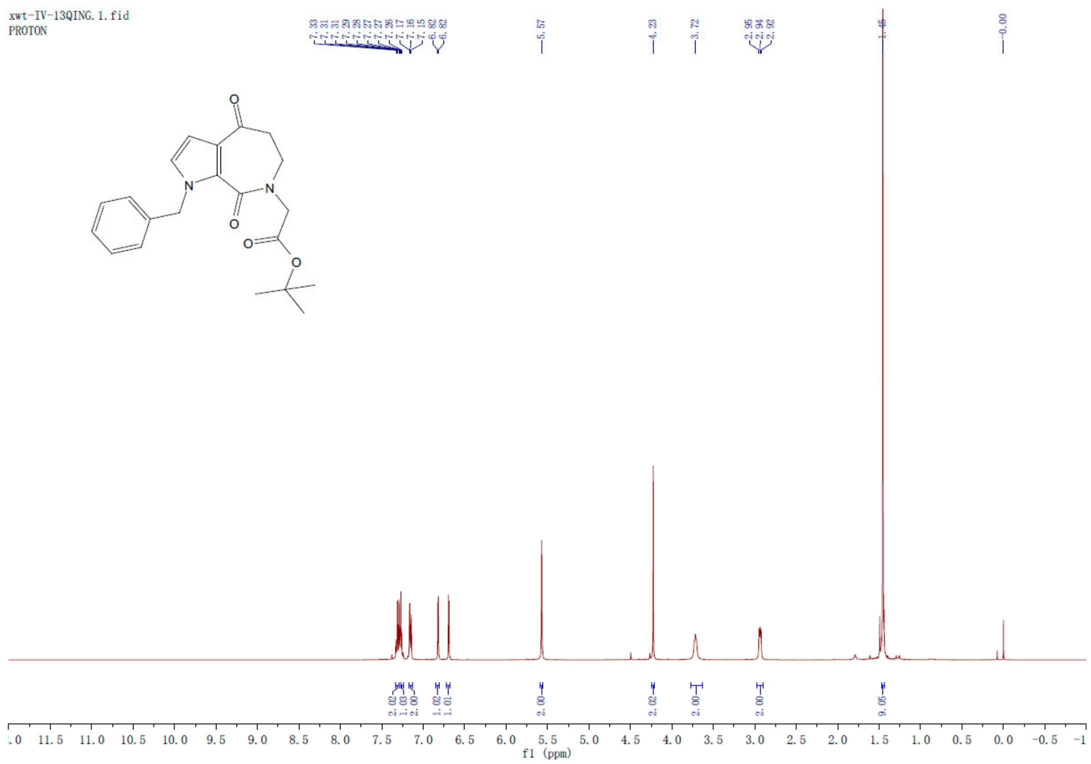

2-5 ^{1}H NMR (400 MHz, $CDCl_3$)

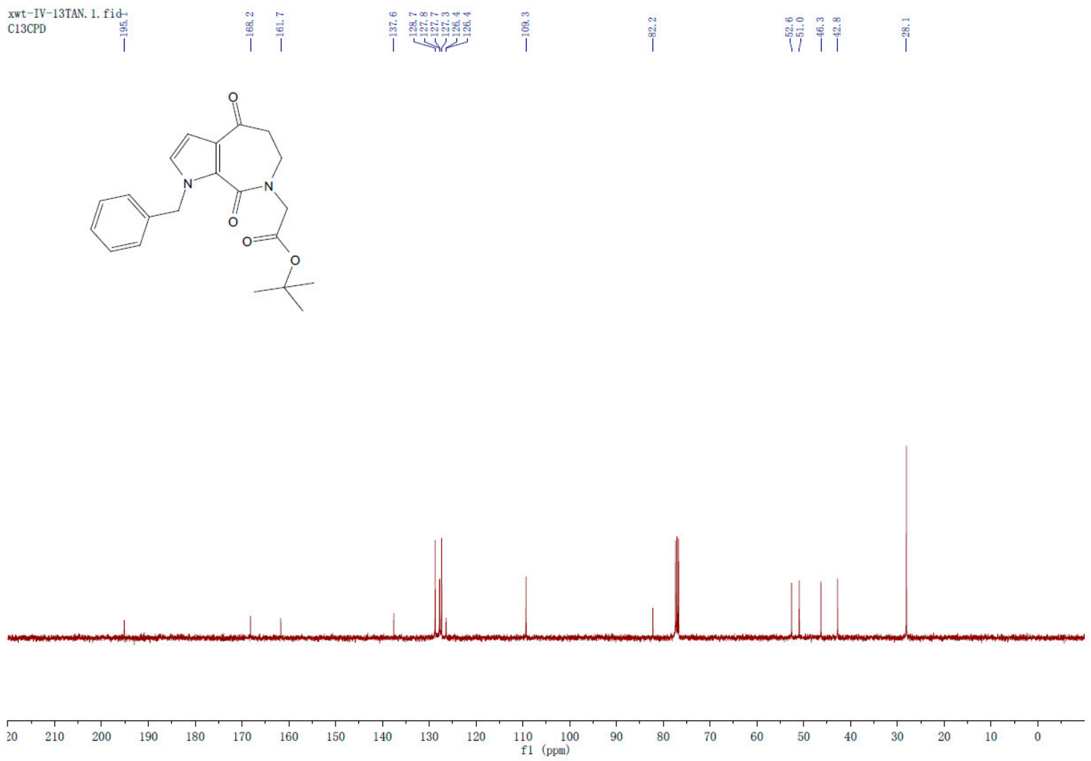

2-5 ^{13}C NMR (100 MHz, $CDCl_3$)

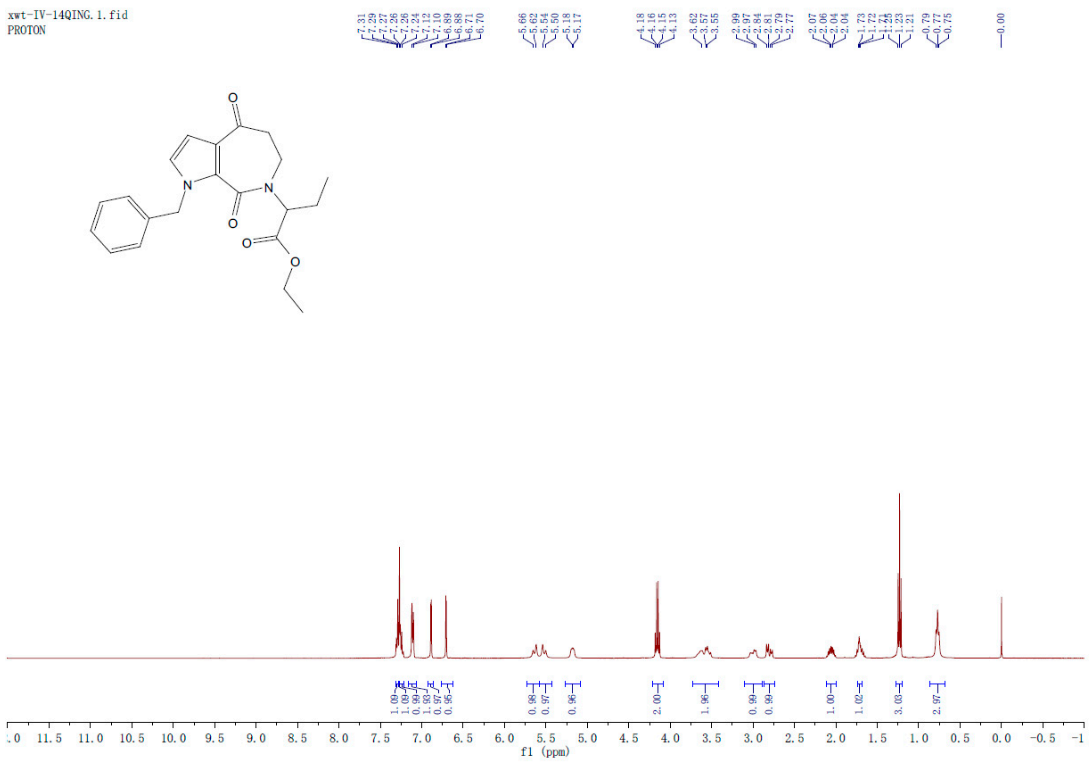

2-6 ^{1}H NMR (400 MHz, $CDCl_3$)

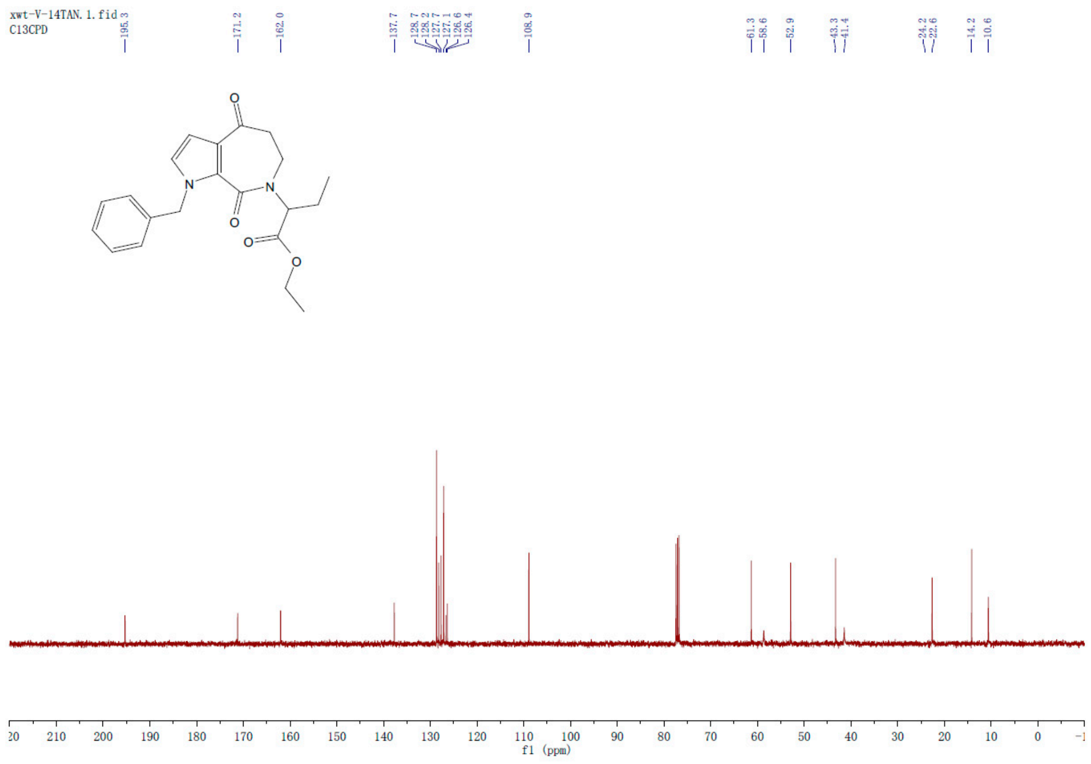

2-6 ^{13}C NMR (100 MHz, $CDCl_3$)

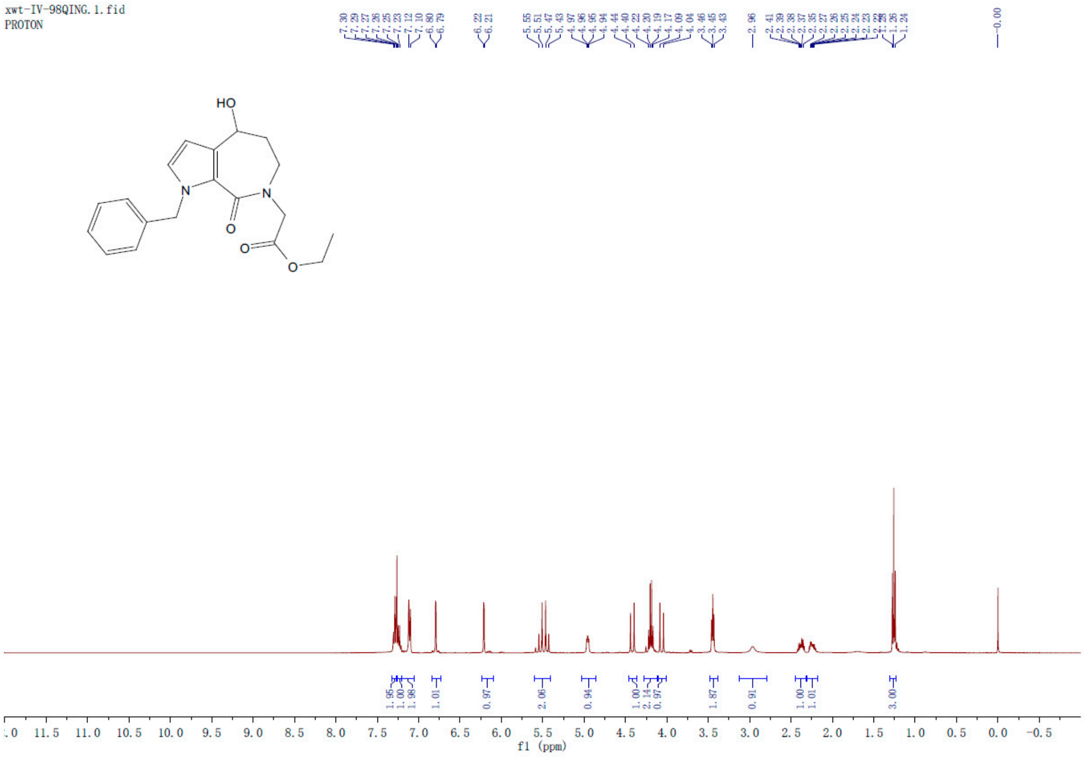

3 ^{1}H NMR (400 MHz, $CDCl_3$)

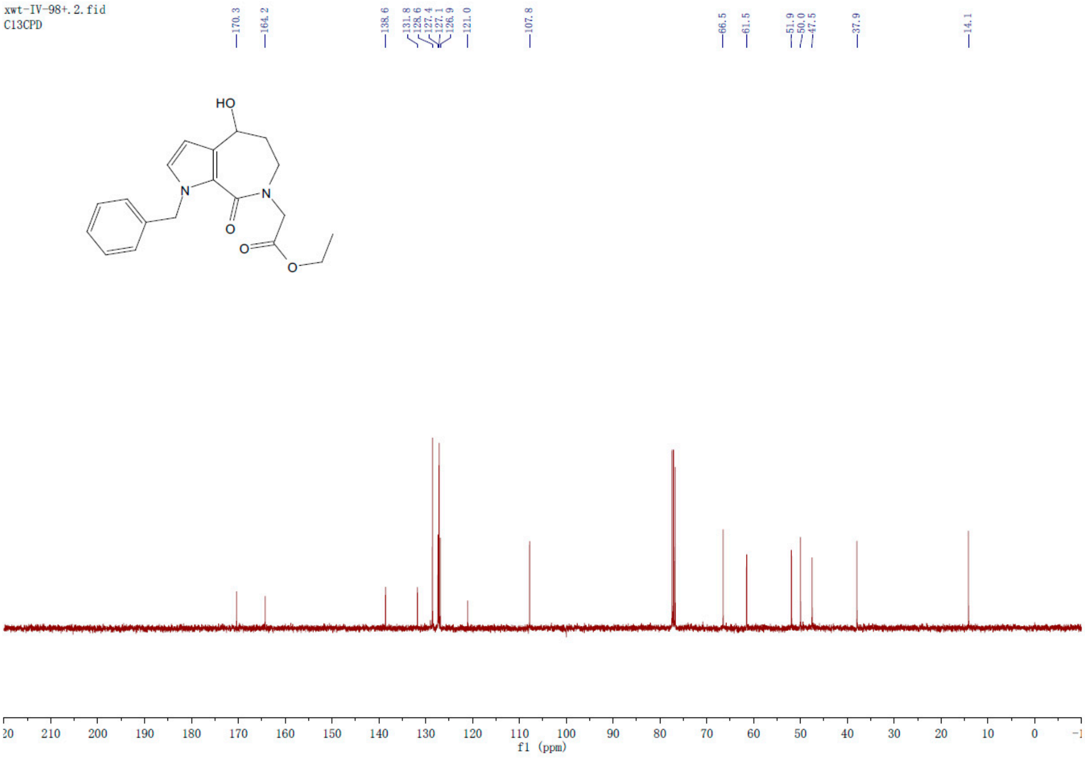

3 ^{13}C NMR (100 MHz, $CDCl_3$)

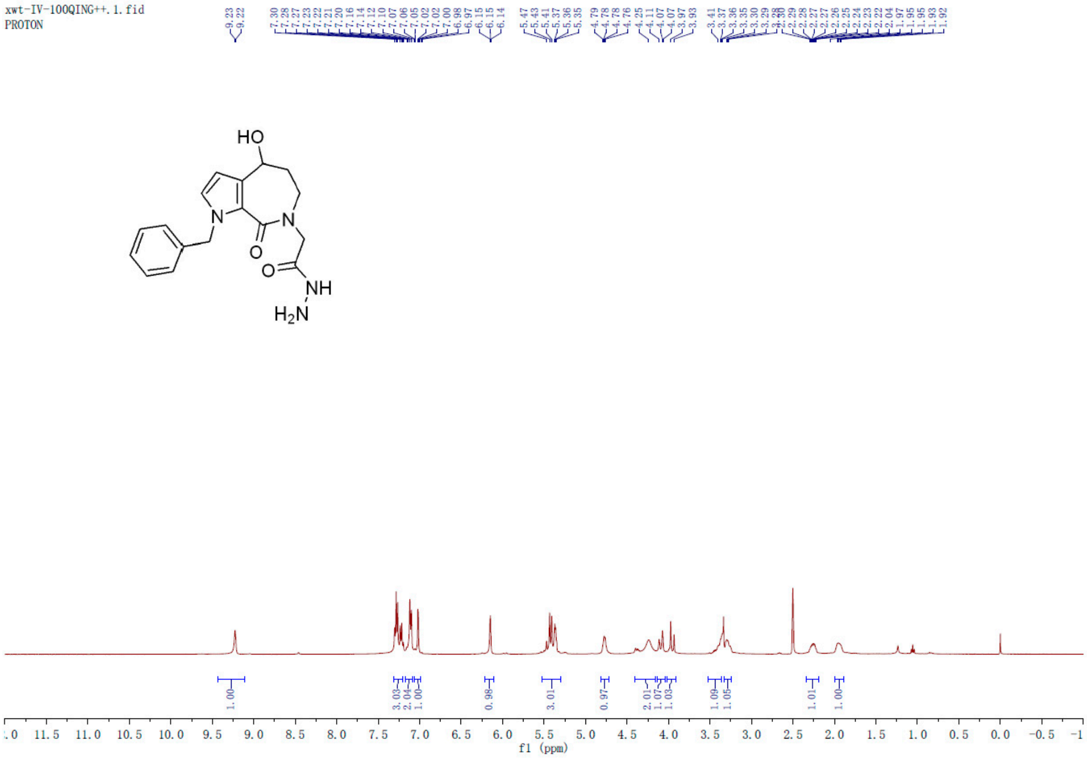

4 ^{1}H NMR (400 MHz, DMSO-d_6)

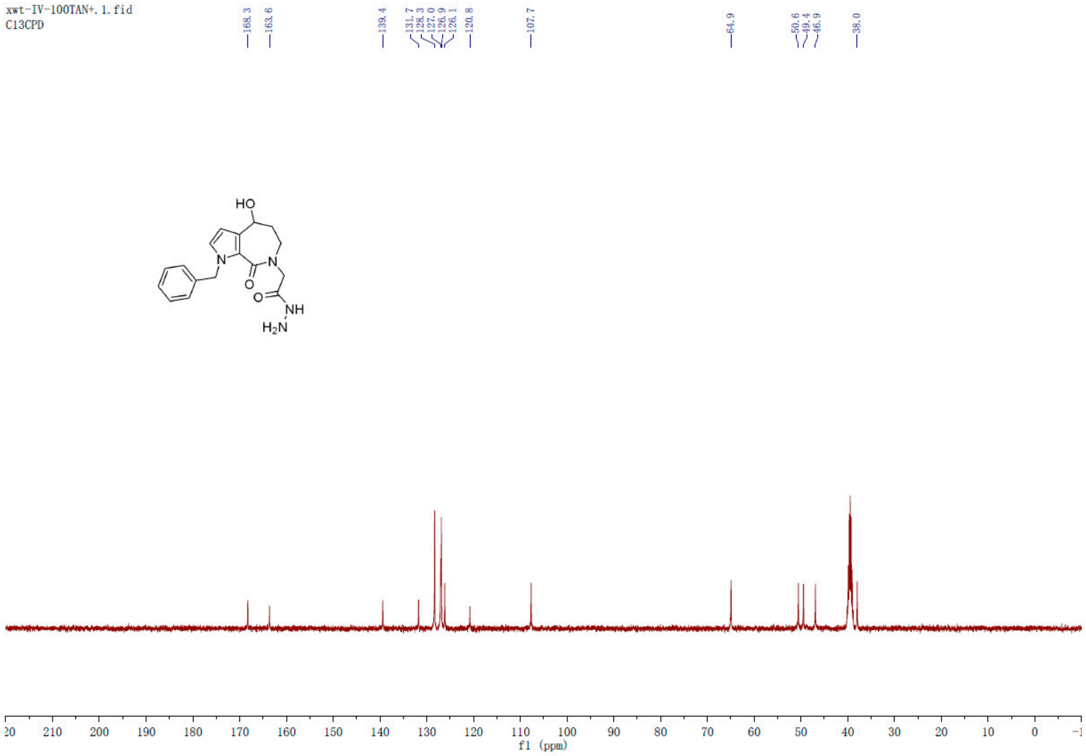

4 ^{13}C NMR (100 MHz, DMSO-d_6)

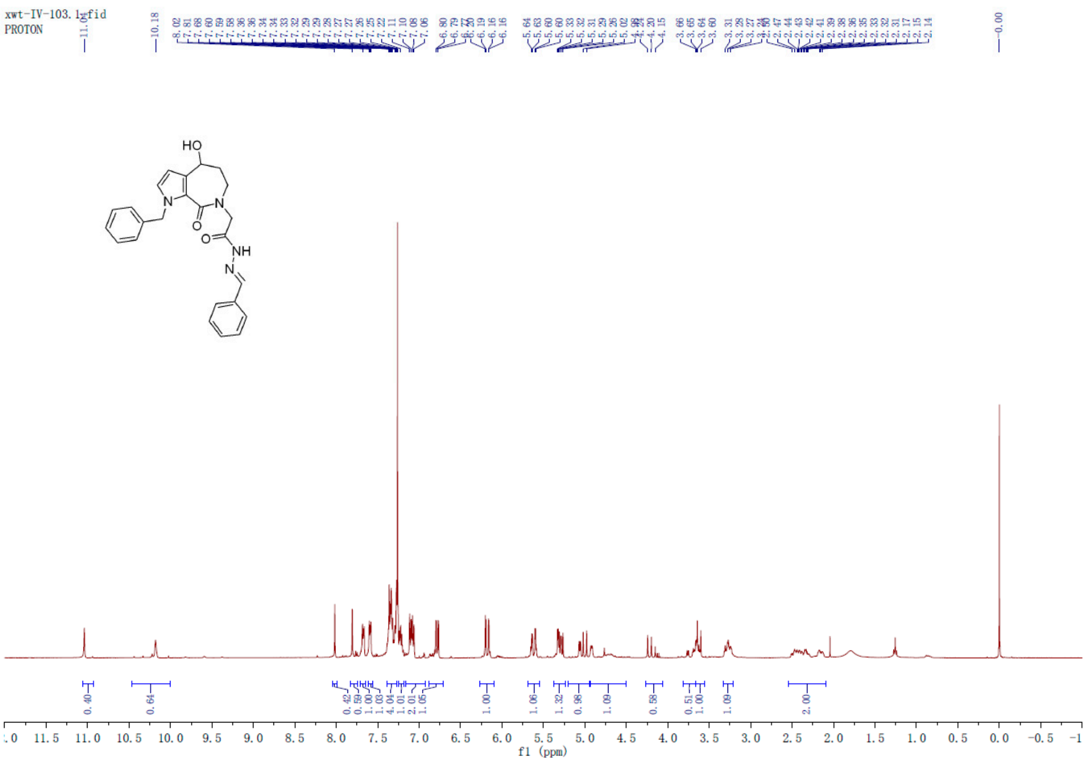

5-1 ^{1}H NMR (400 MHz, $CDCl_3$)

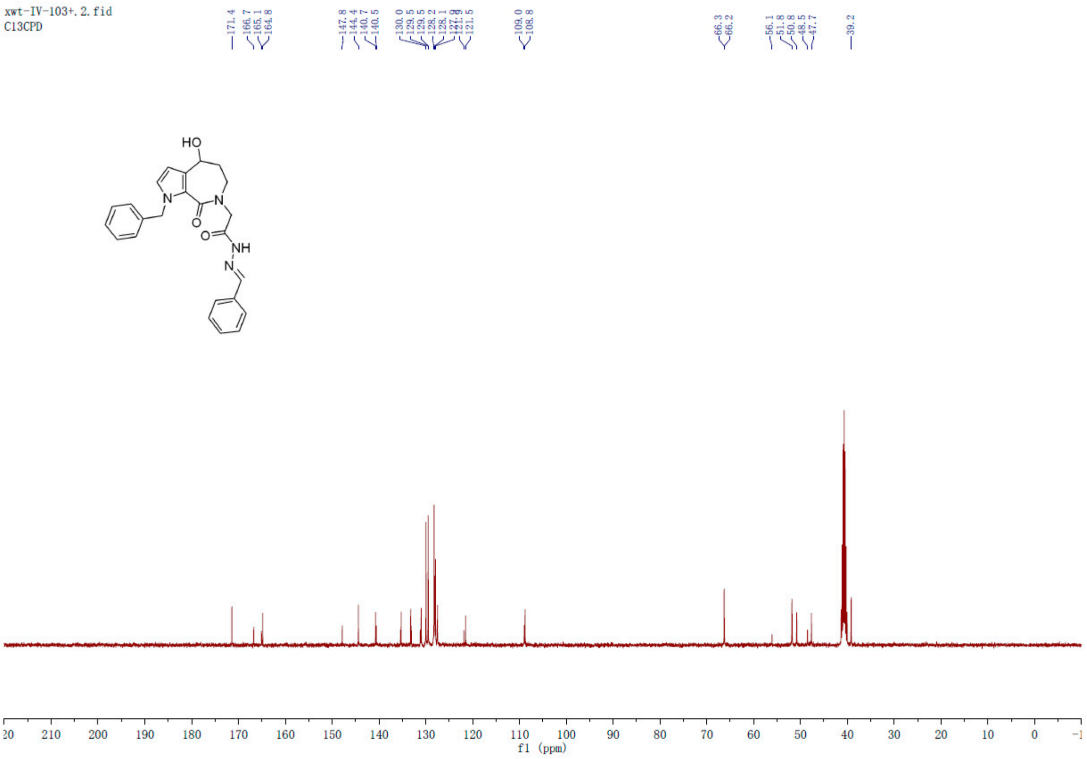

5-1 ^{13}C NMR (100 MHz, $CDCl_3$)

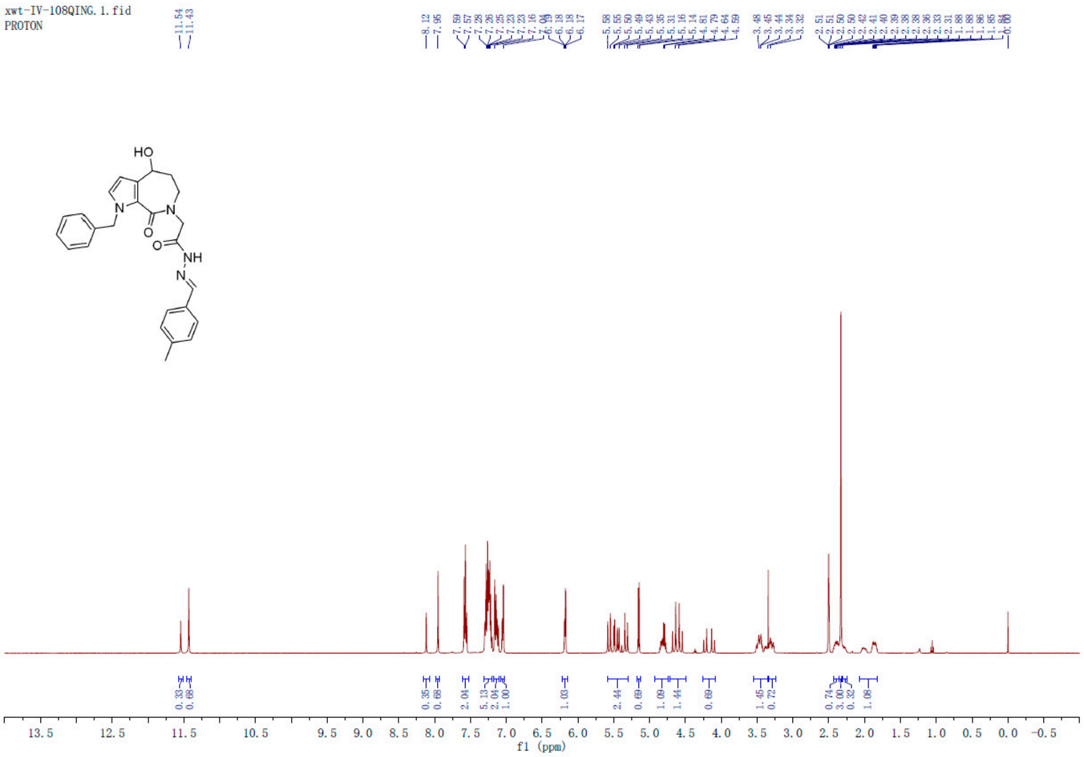

5-2 ^{1}H NMR (400 MHz, DMSO-*d*6)

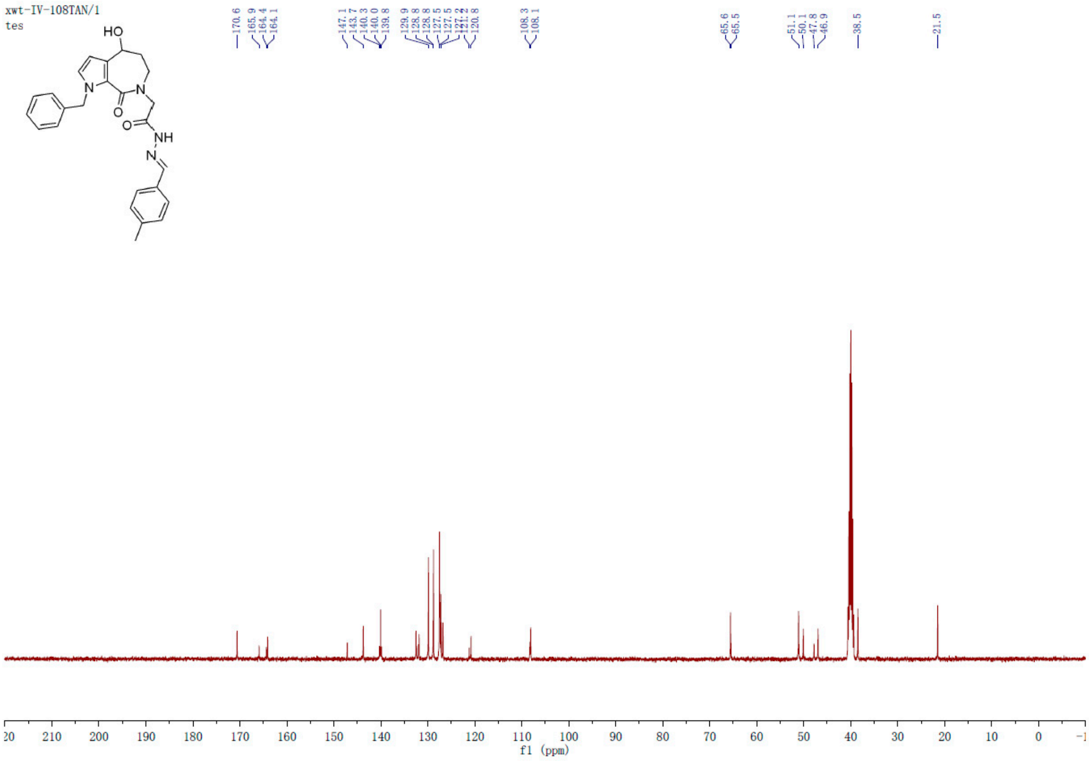

5-2 ^{13}C NMR (100 MHz, DMSO-*d*6)

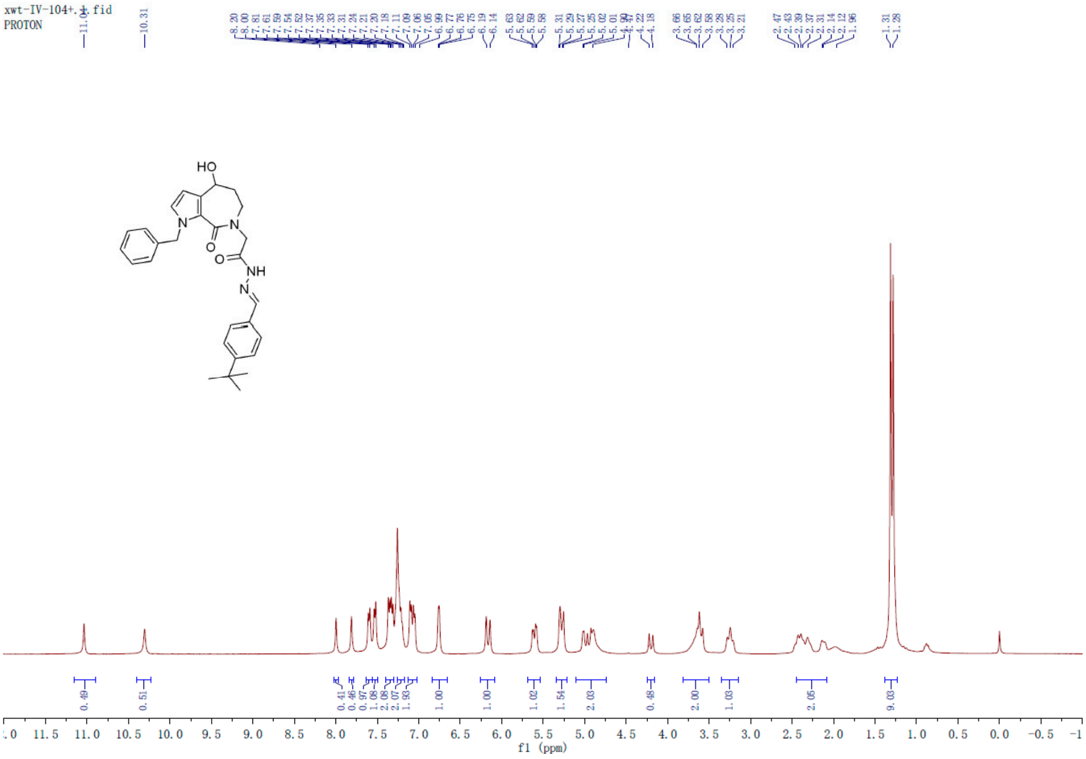

5-3 ^{1}H NMR (400 MHz, $CDCl_3$)

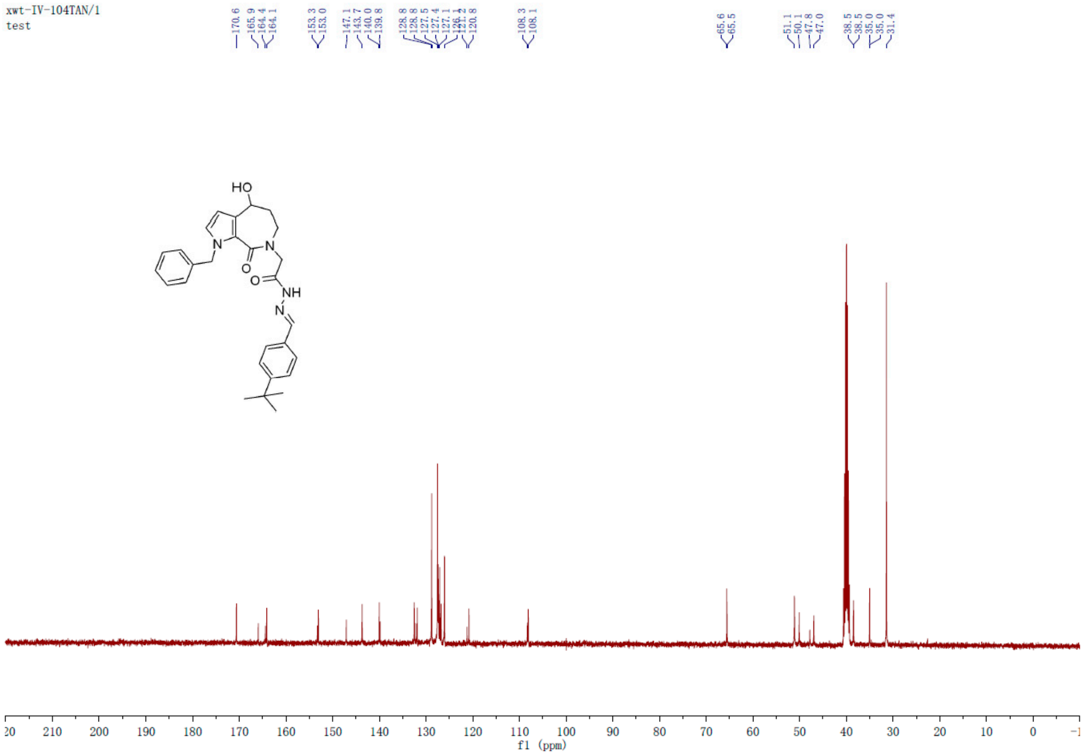

5-3 ^{13}C NMR (100 MHz, $CDCl_3$)

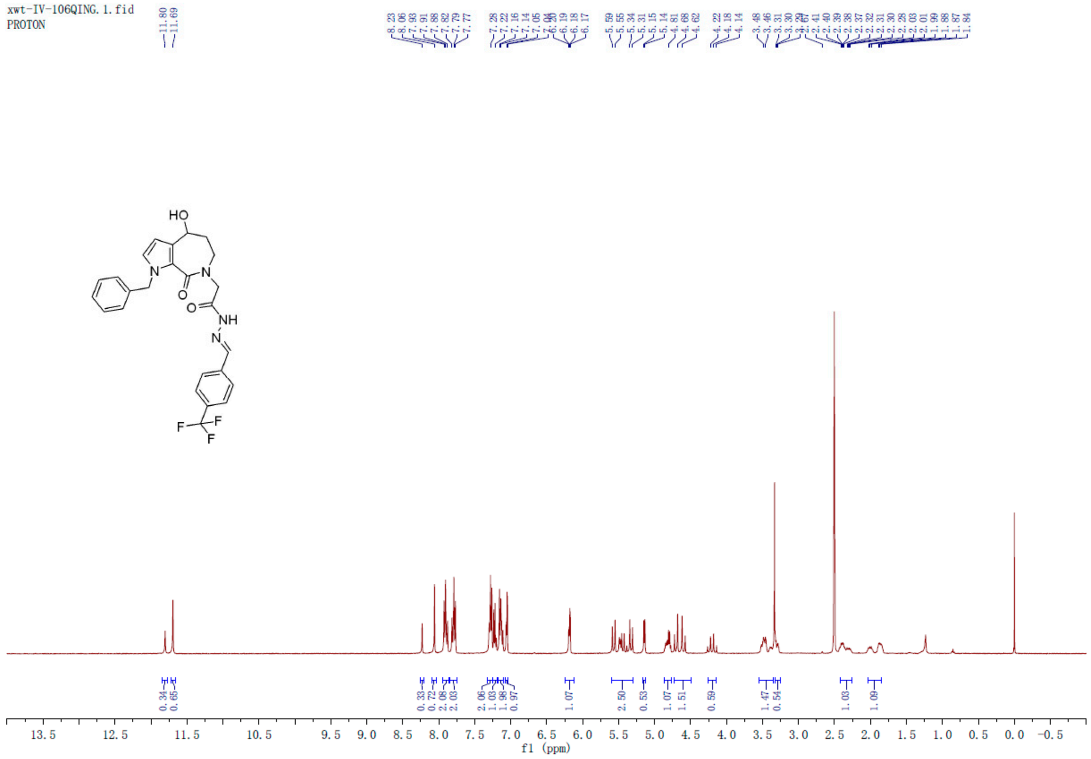

5-4 ^{1}H NMR (400 MHz, DMSO-d_6)

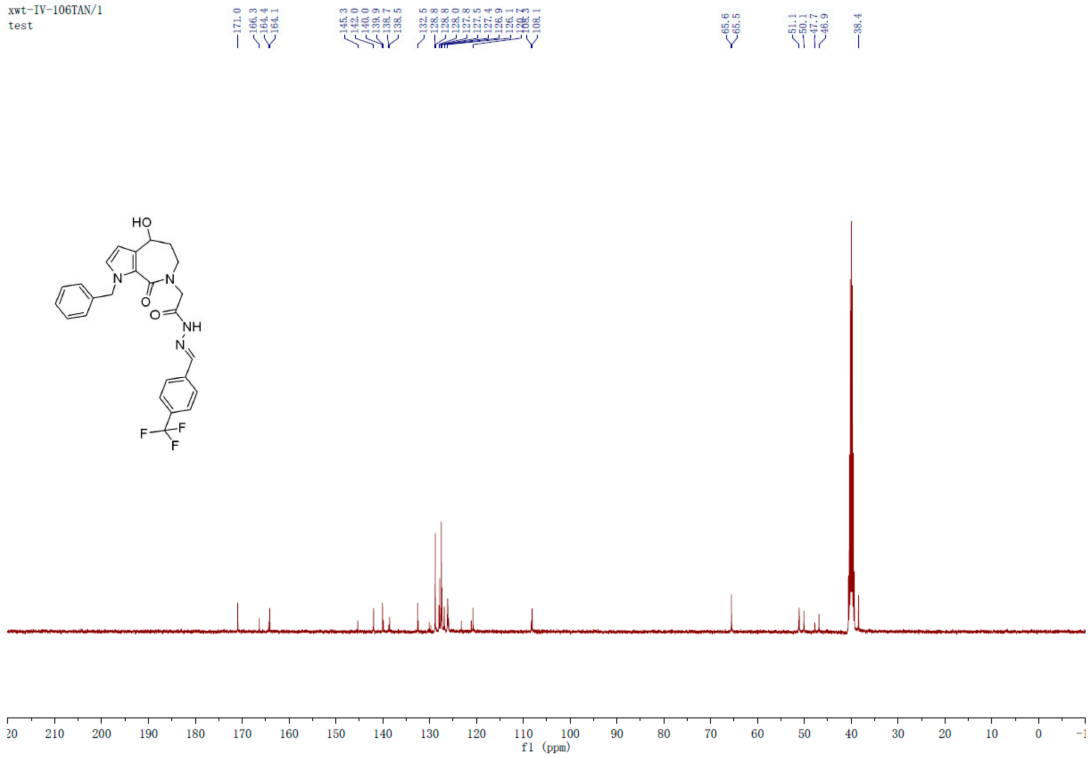

5-4 ^{13}C NMR (100 MHz, DMSO-d_6)

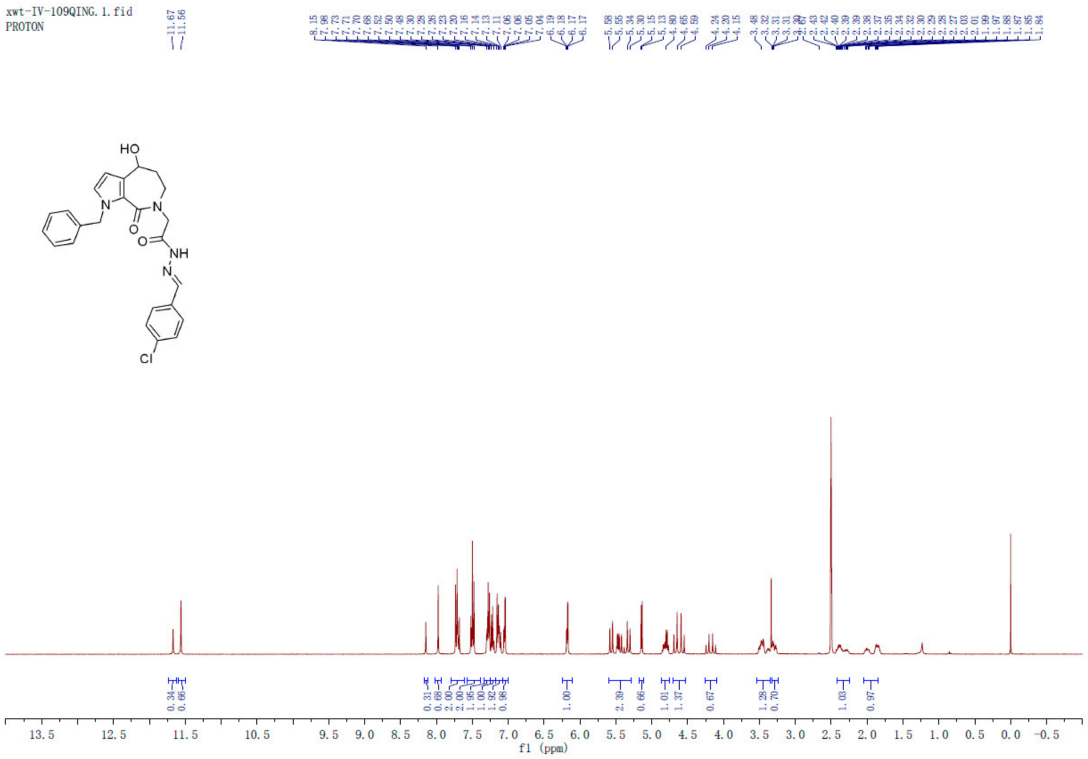

5-5 ^{1}H NMR (400 MHz, DMSO-d_6)

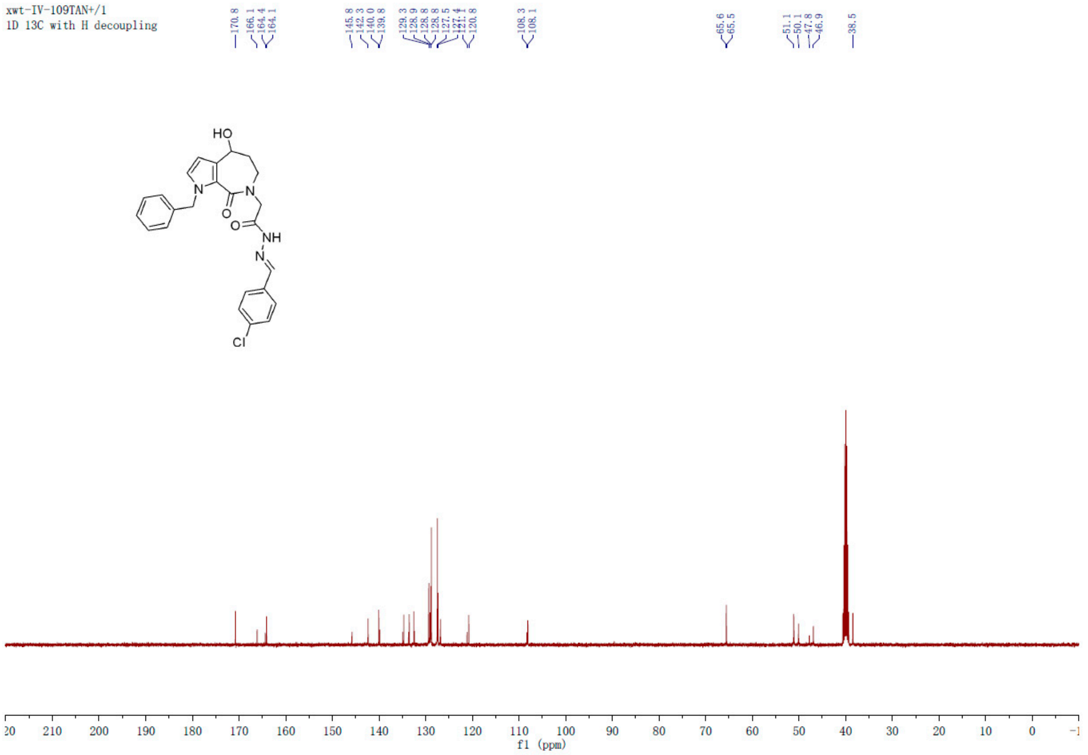

5-5 ^{13}C NMR (100 MHz, DMSO-d_6)

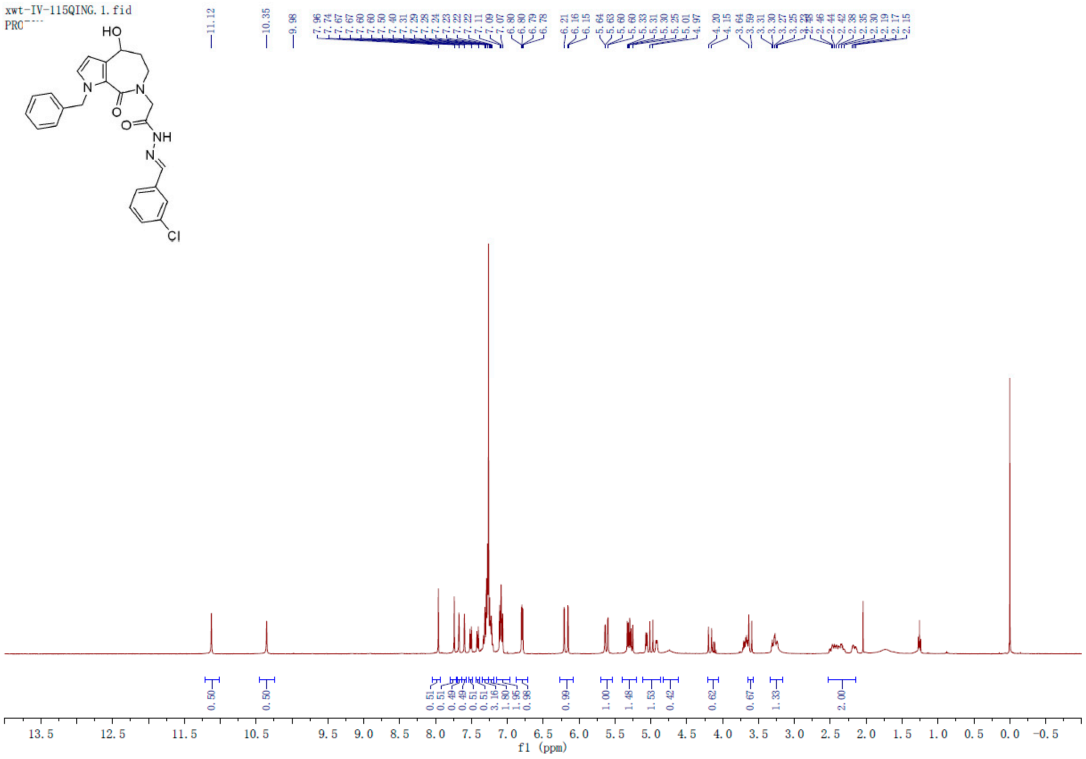

5-6 ^{1}H NMR (400 MHz, $CDCl_3$)

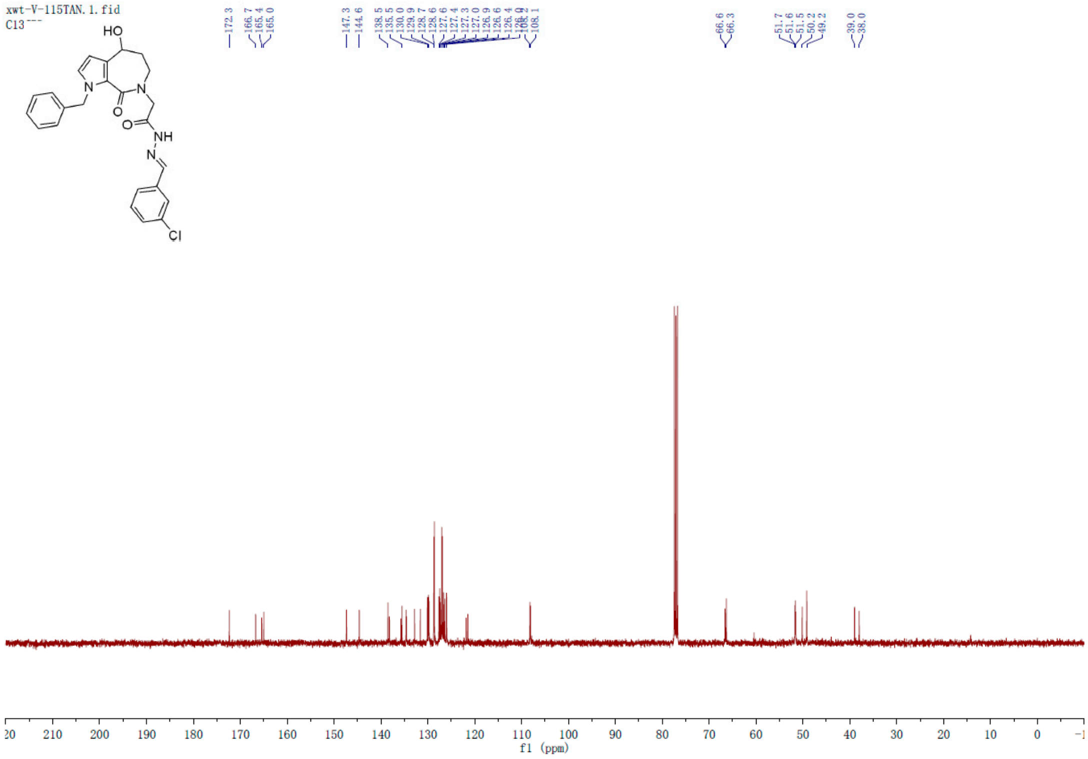

5-6 ^{13}C NMR (100 MHz, $CDCl_3$)

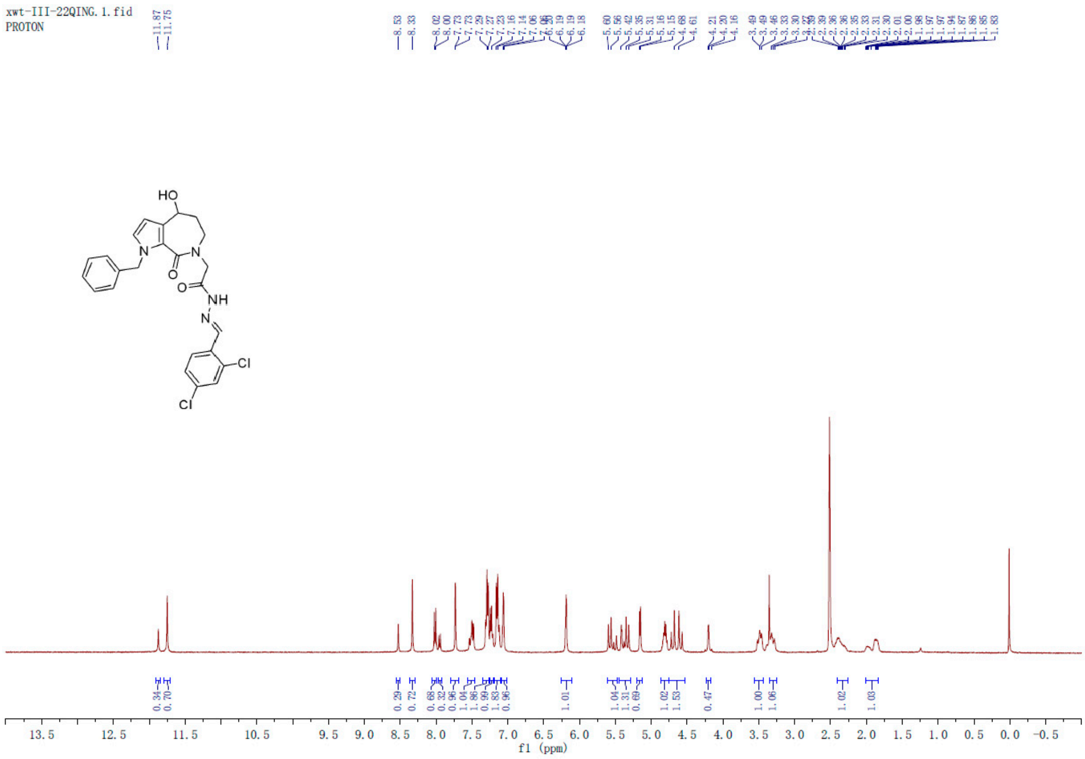

5-7 ^{1}H NMR (400 MHz, DMSO-d_6)

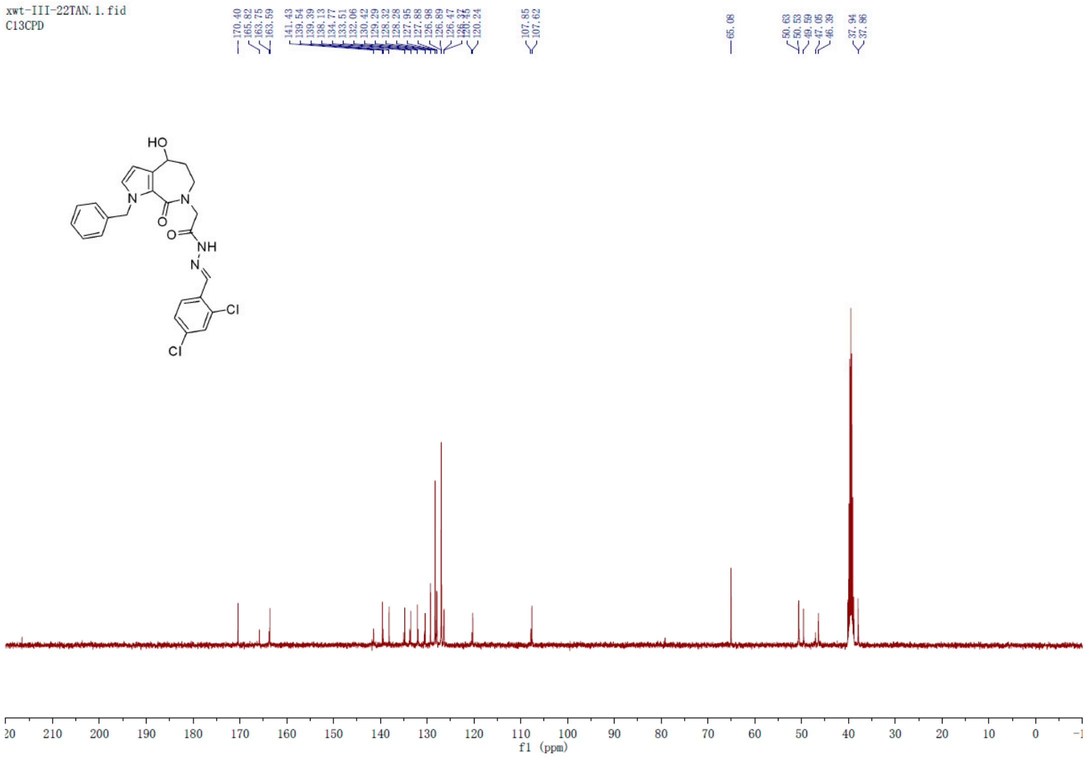

5-7 ^{13}C NMR (100 MHz, DMSO-d_6)

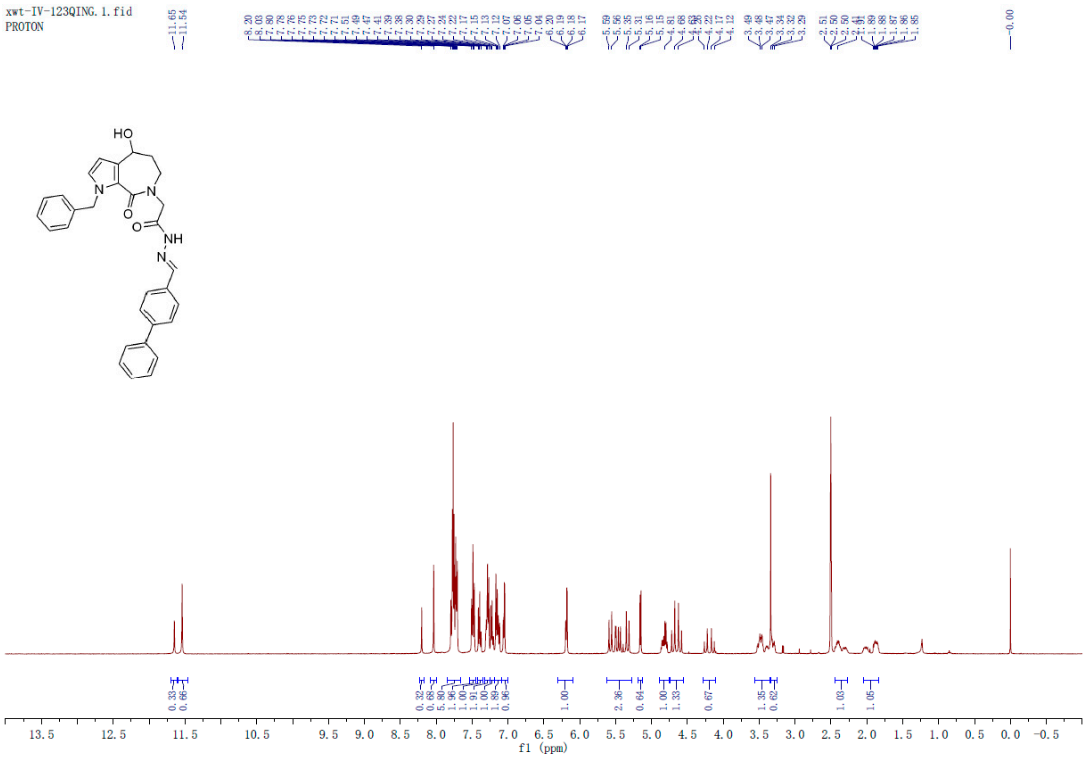

5-8 ^{1}H NMR (400 MHz, DMSO-d_6)

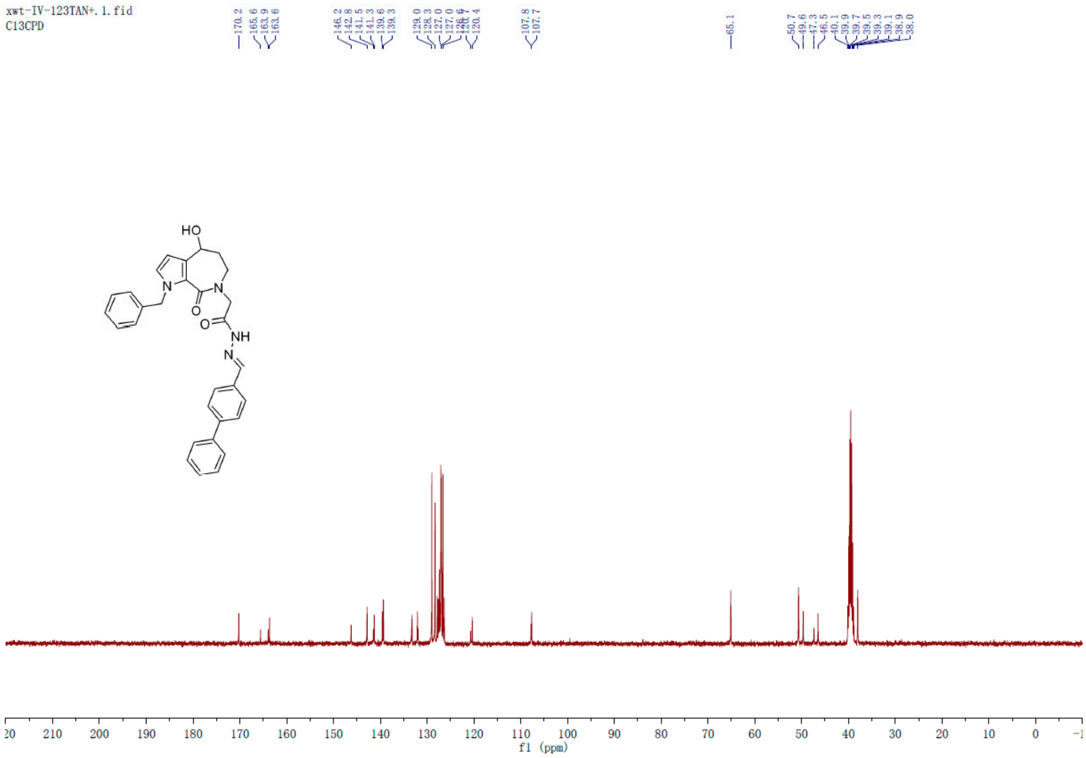

5-8 ^{13}C NMR (100 MHz, DMSO-d_6)

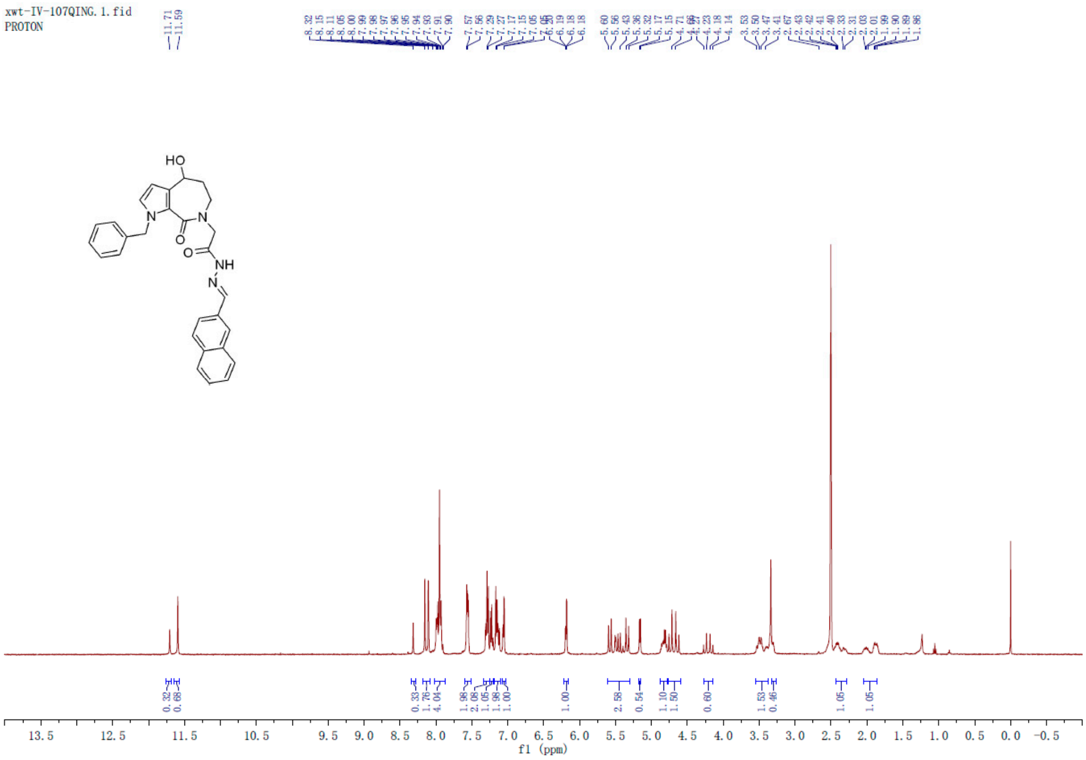

5-9 ^{1}H NMR (400 MHz, DMSO-*d*6)

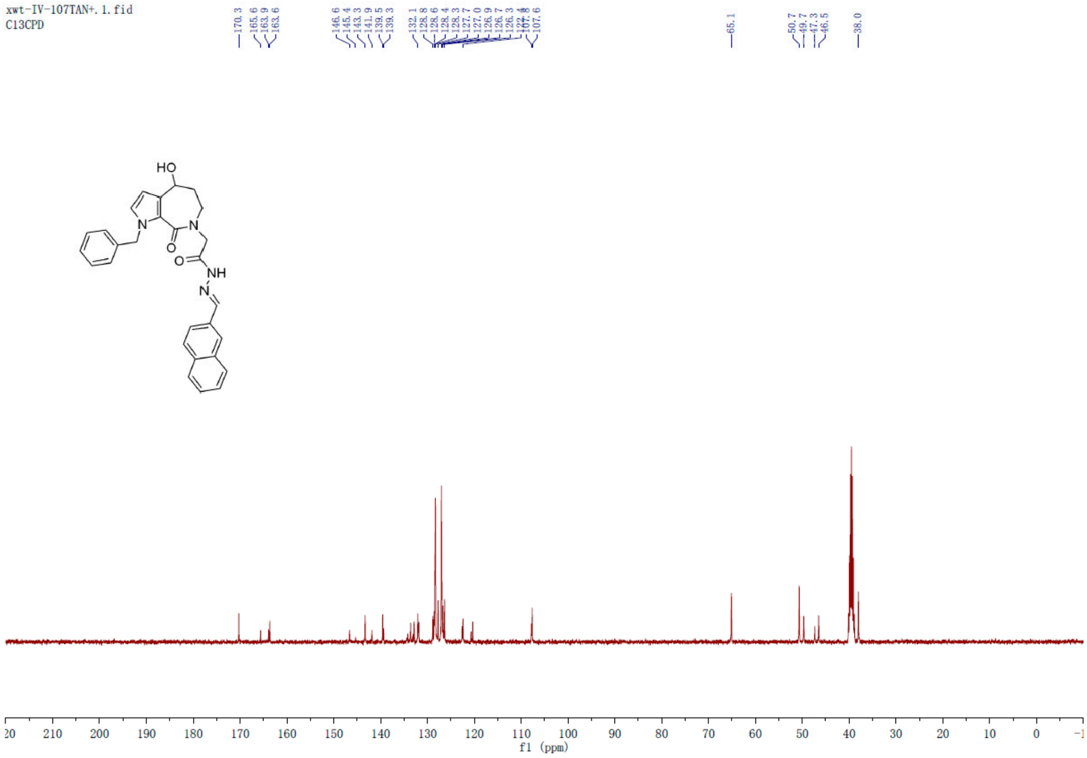

5-9 ^{13}C NMR (100 MHz, DMSO-*d*6)

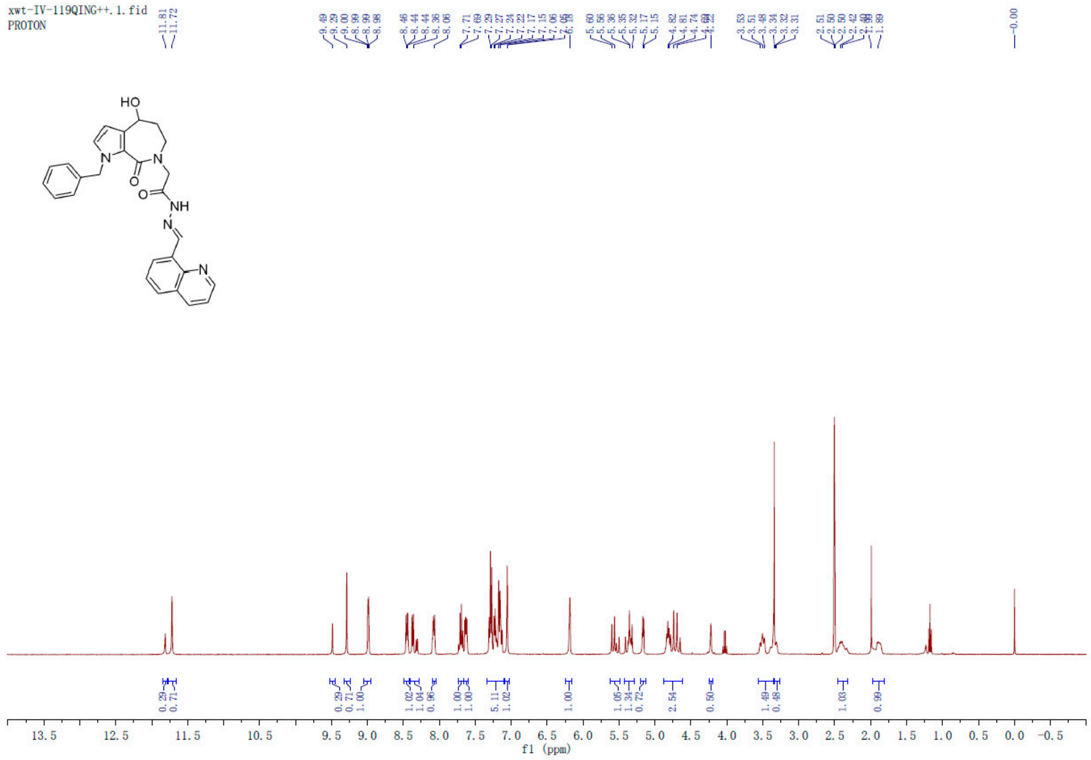

5-10 ^{1}H NMR (400 MHz, DMSO-d_6)

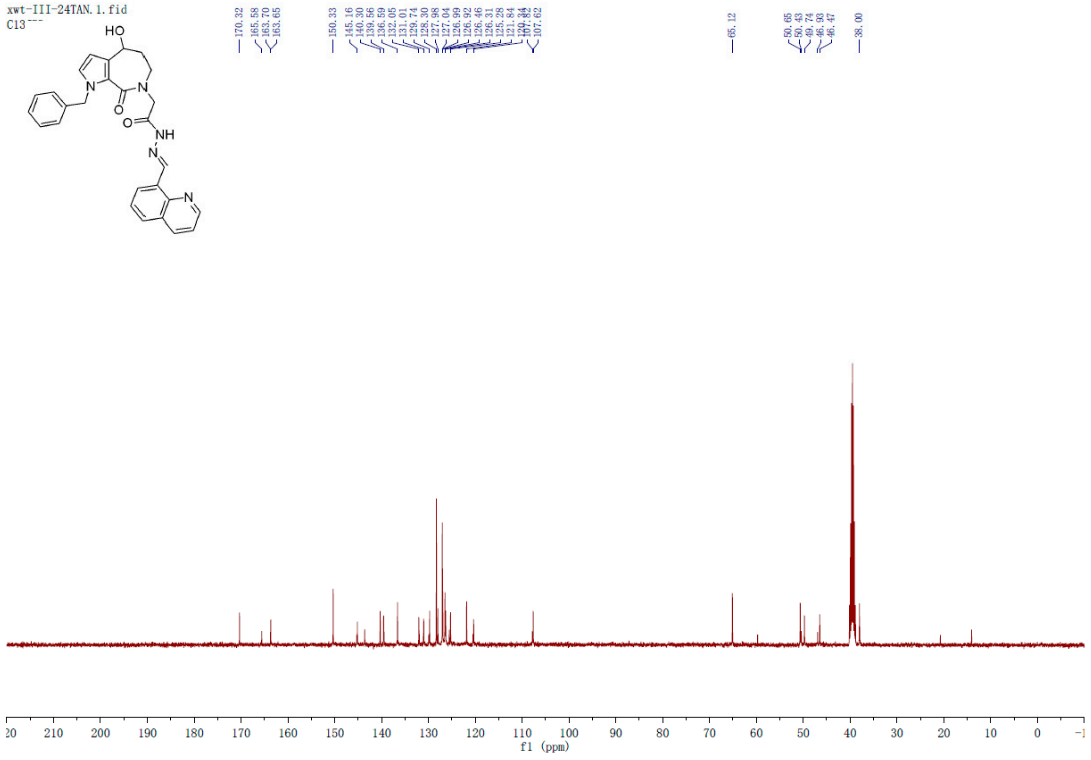

5-10 ^{13}C NMR (100 MHz, DMSO-d_6)

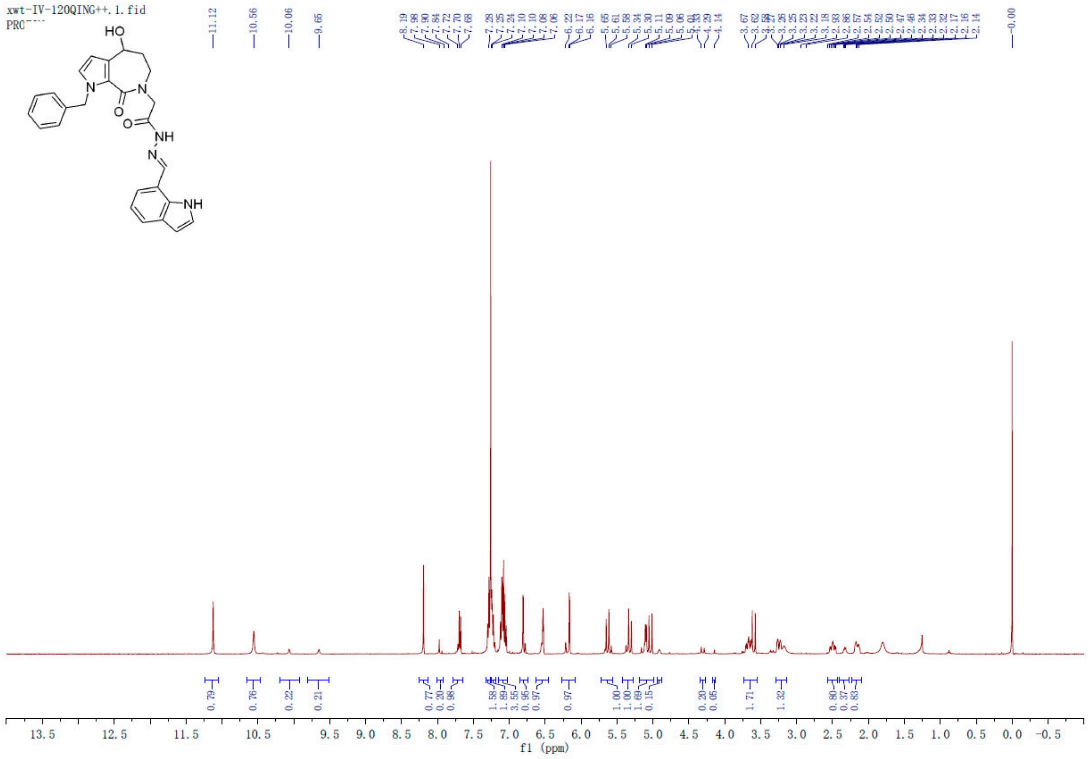

5-11 ^{1}H NMR (400 MHz, $CDCl_3$)

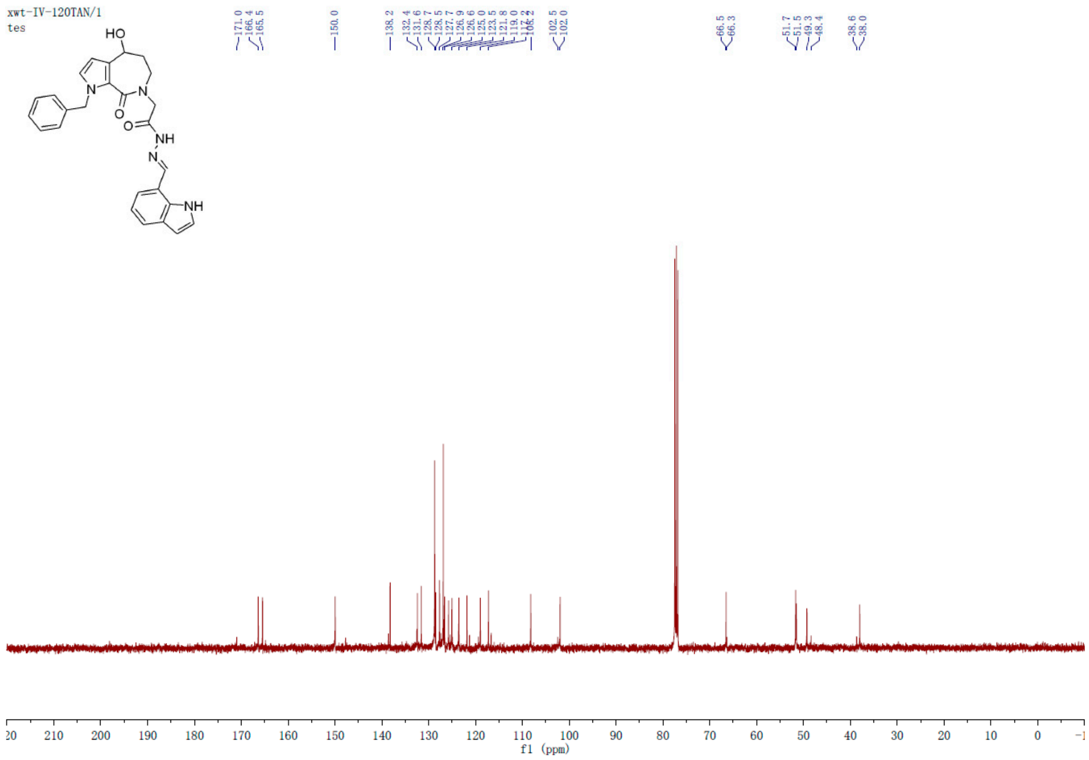

5-11 ^{13}C NMR (100 MHz, $CDCl_3$)

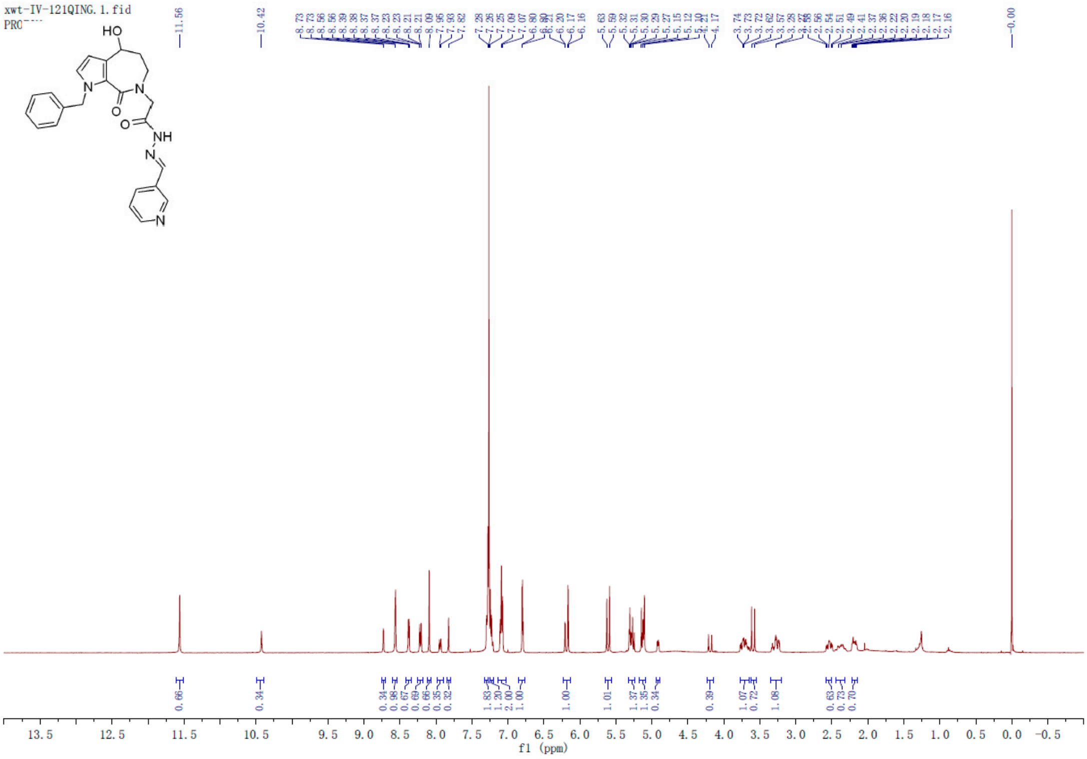

5-12 ^{1}H NMR (400 MHz, CDCl$_3$)

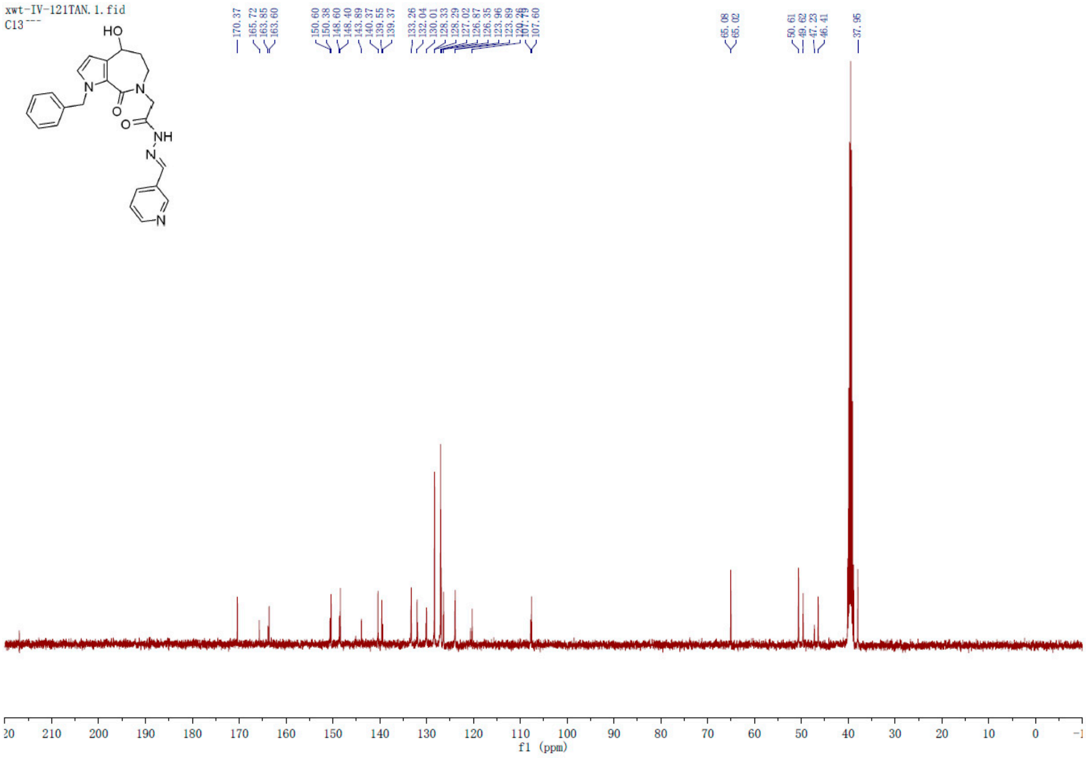

5-12 ^{13}C NMR (100 MHz, CDCl$_3$)

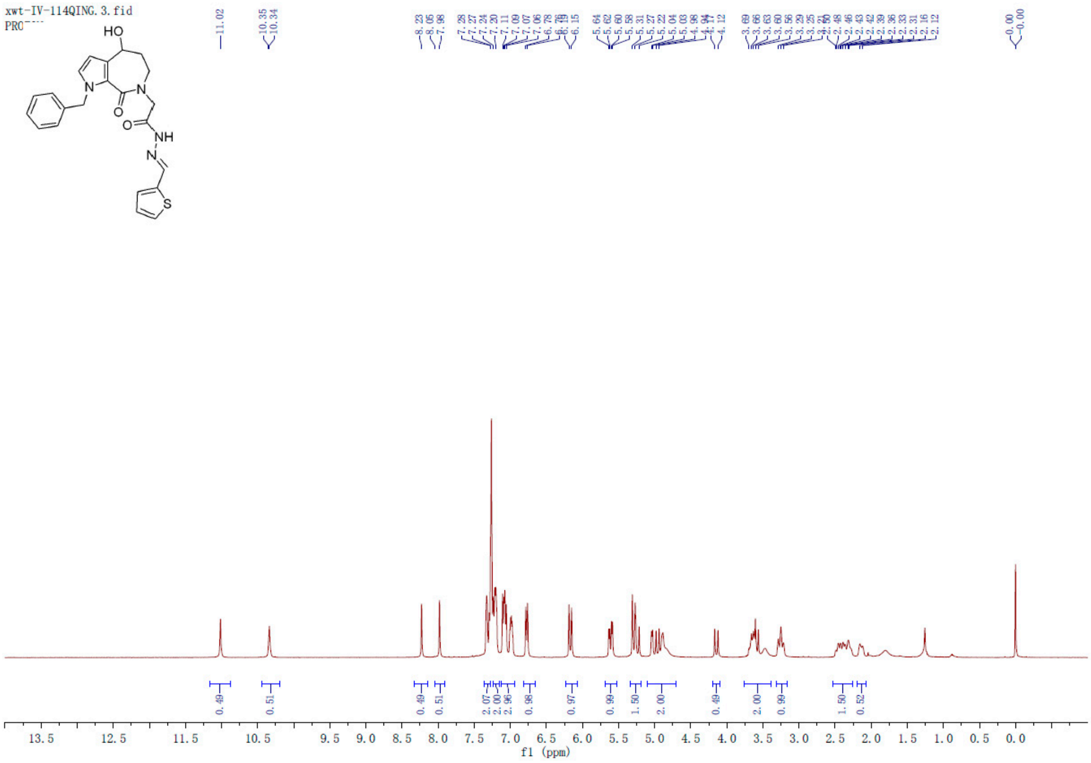

5-13 ^{1}H NMR (400 MHz, $CDCl_3$)

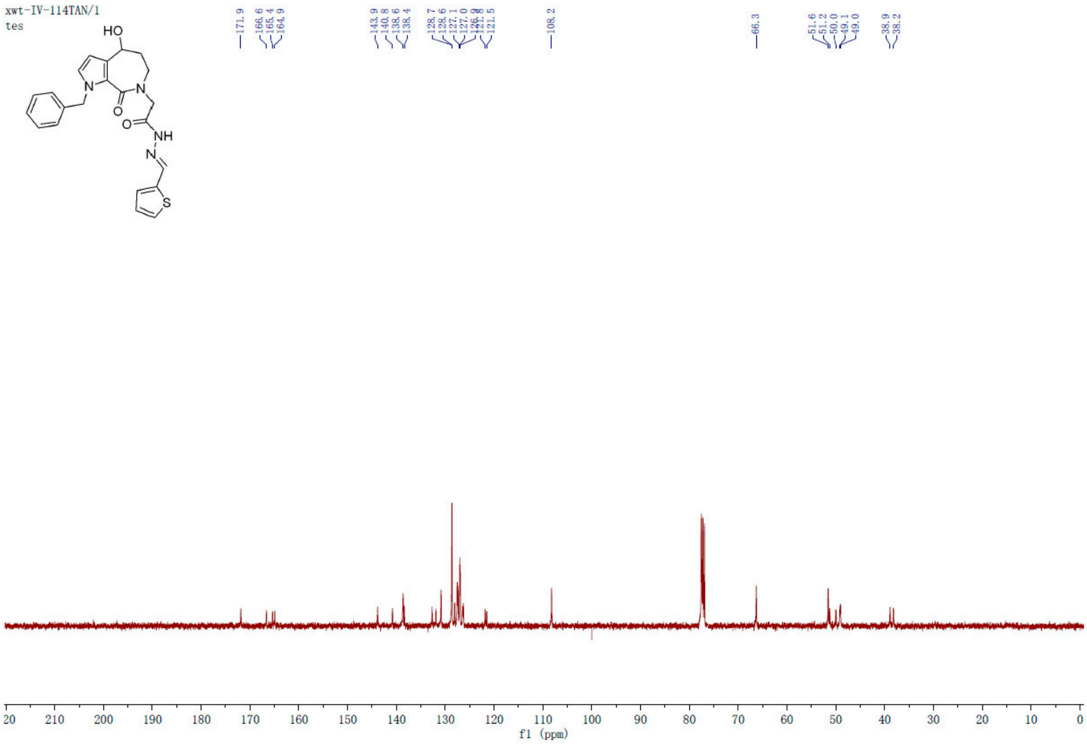

5-13 ^{13}C NMR (100 MHz, $CDCl_3$)

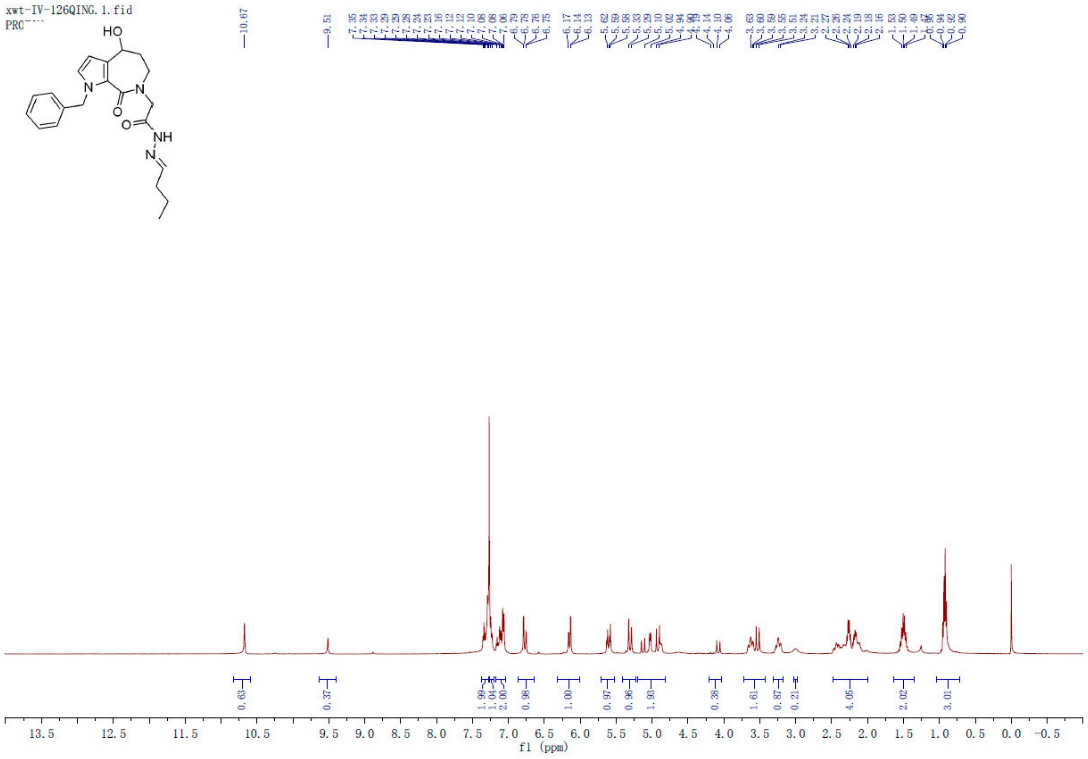

5-14 ^{1}H NMR (400 MHz, $CDCl_3$)

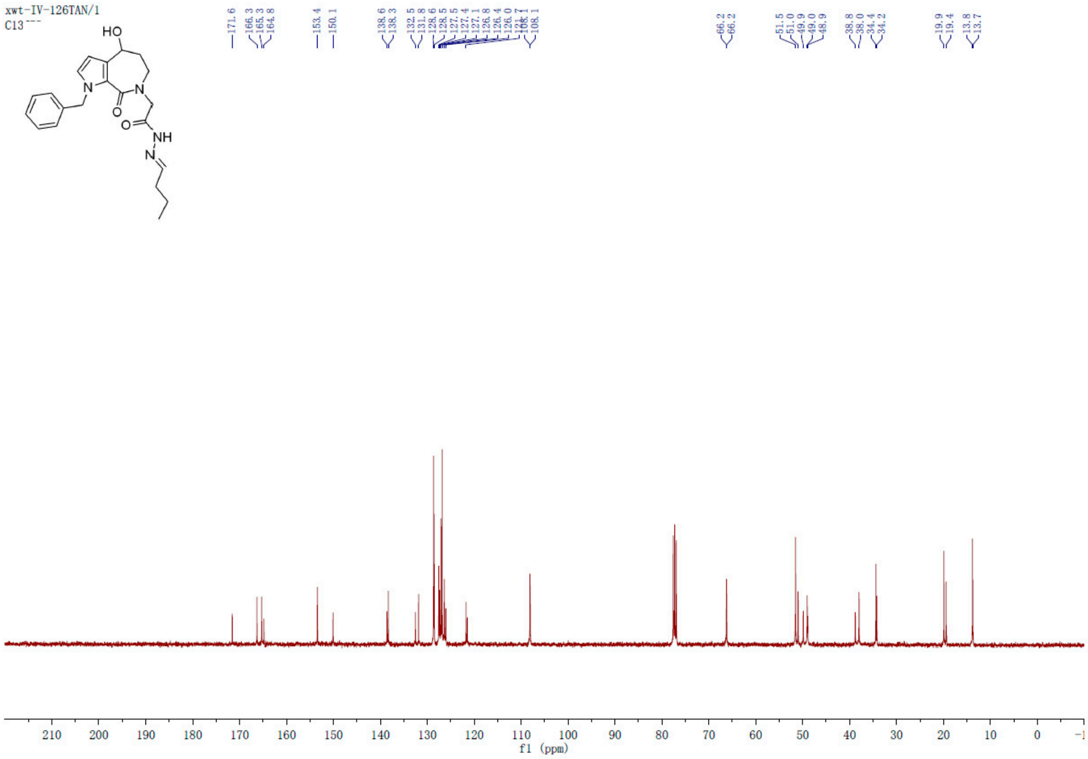

5-14 ^{13}C NMR (100 MHz, $CDCl_3$)

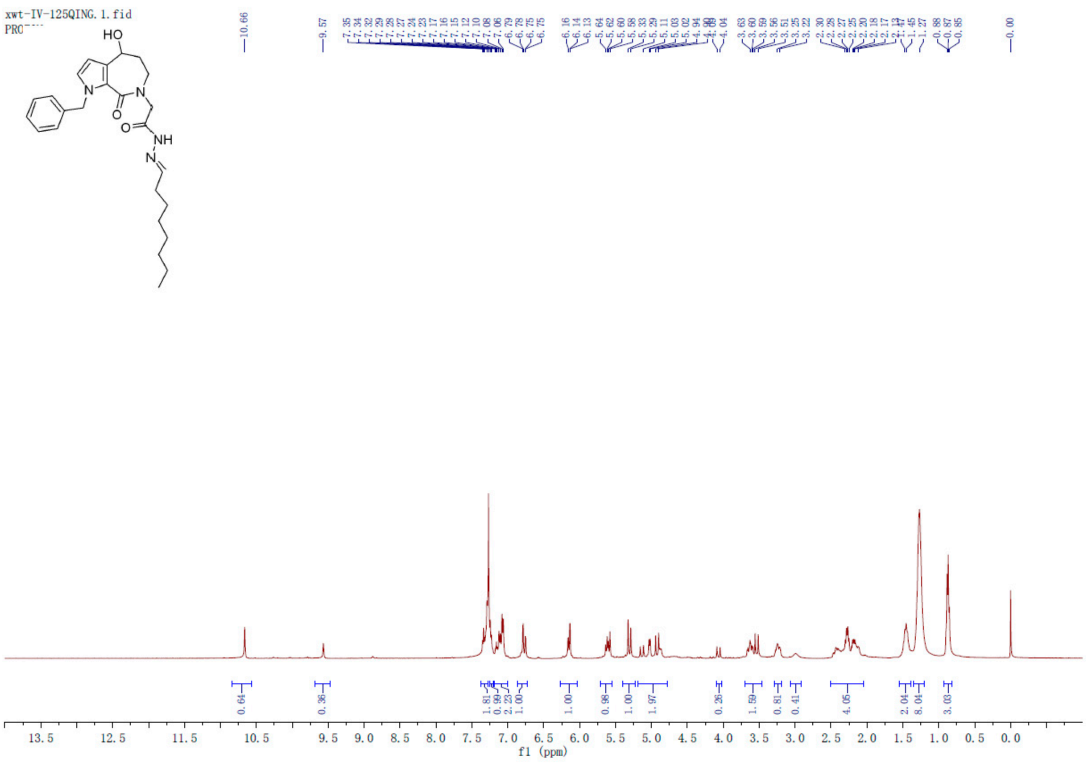

5-15 ^{1}H NMR (400 MHz, $CDCl_3$)

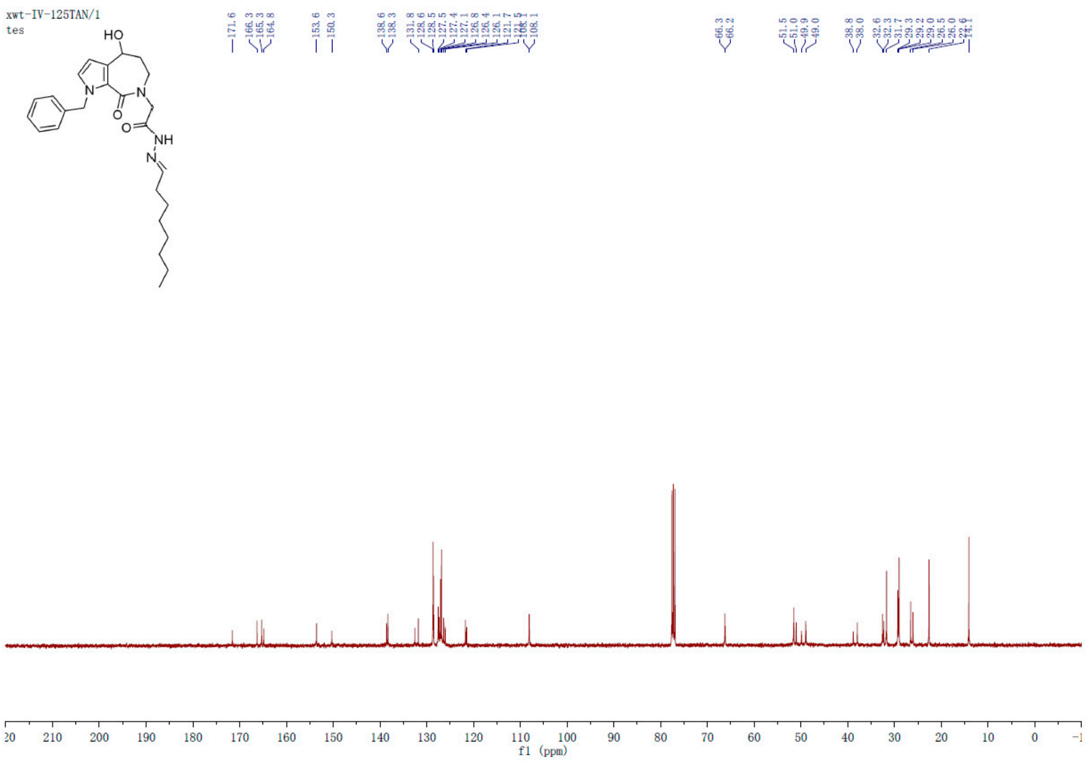

5-15 ^{13}C NMR (100 MHz, $CDCl_3$)

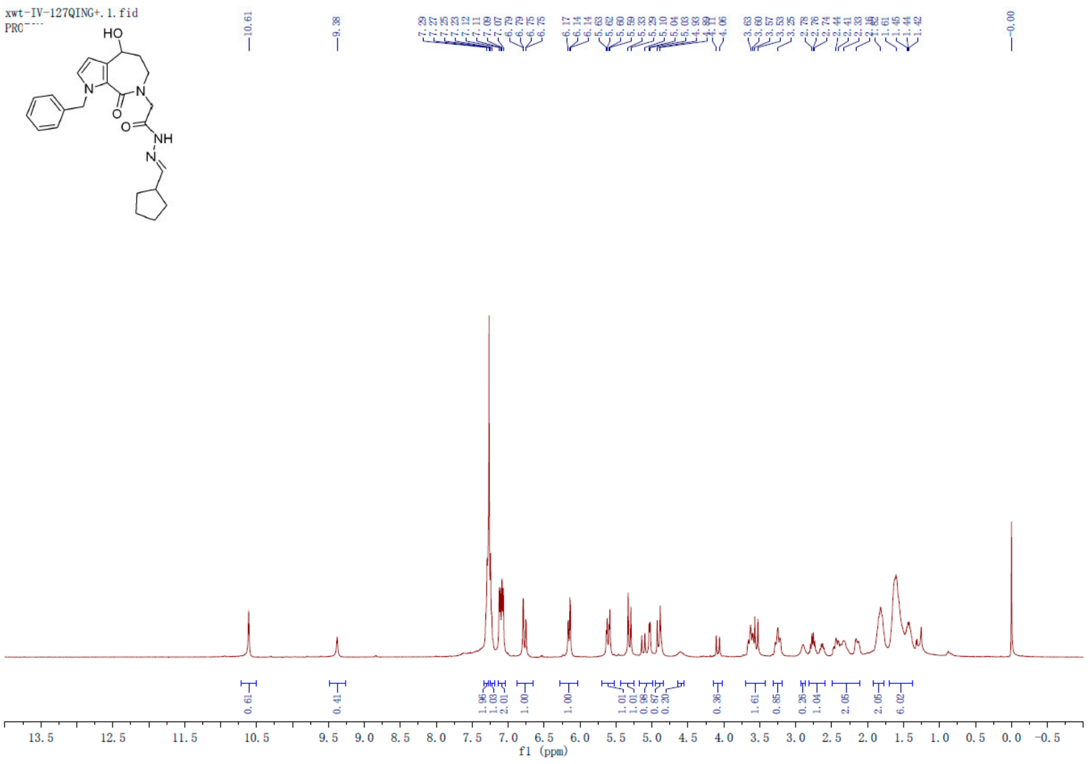

5-16 ^{1}H NMR (400 MHz, $CDCl_3$)

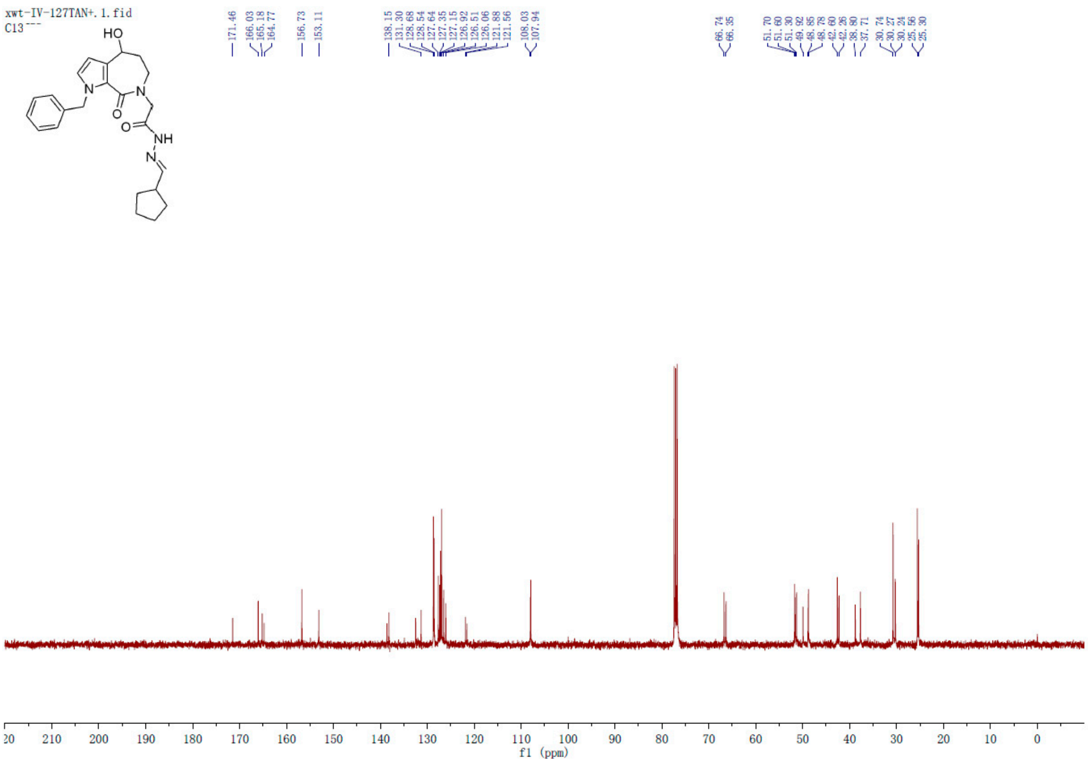

5-16 ^{13}C NMR (100 MHz, $CDCl_3$)

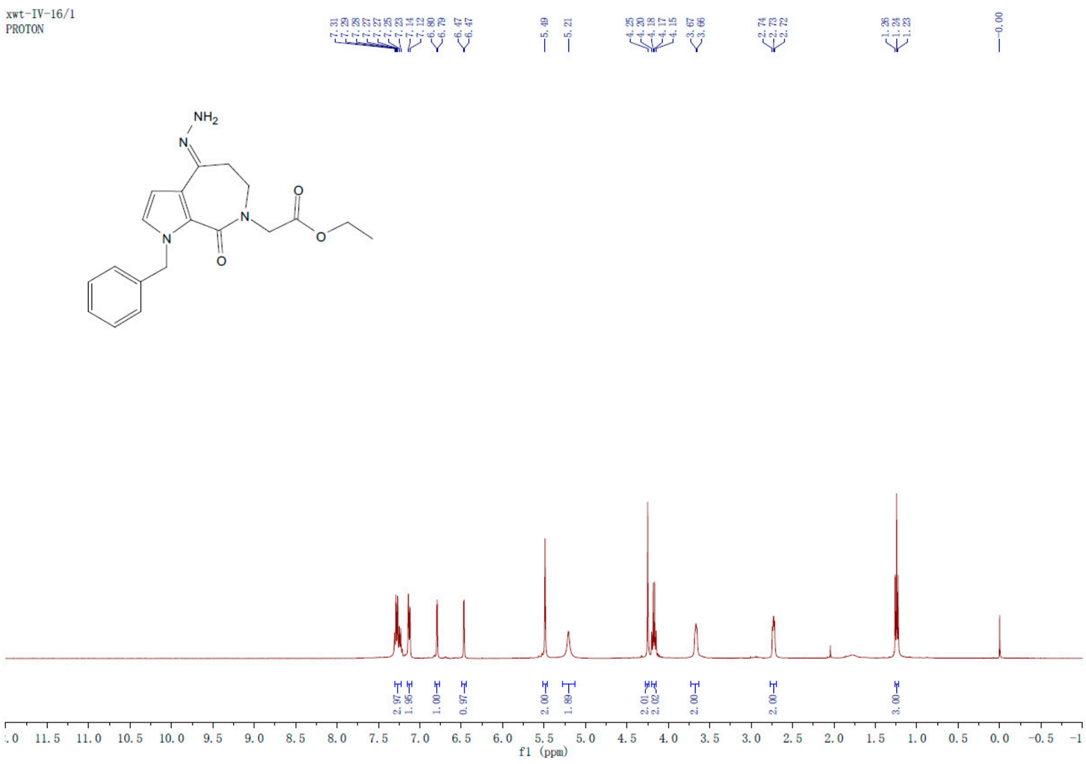

5-17 ^{1}H NMR (400 MHz, $CDCl_3$)

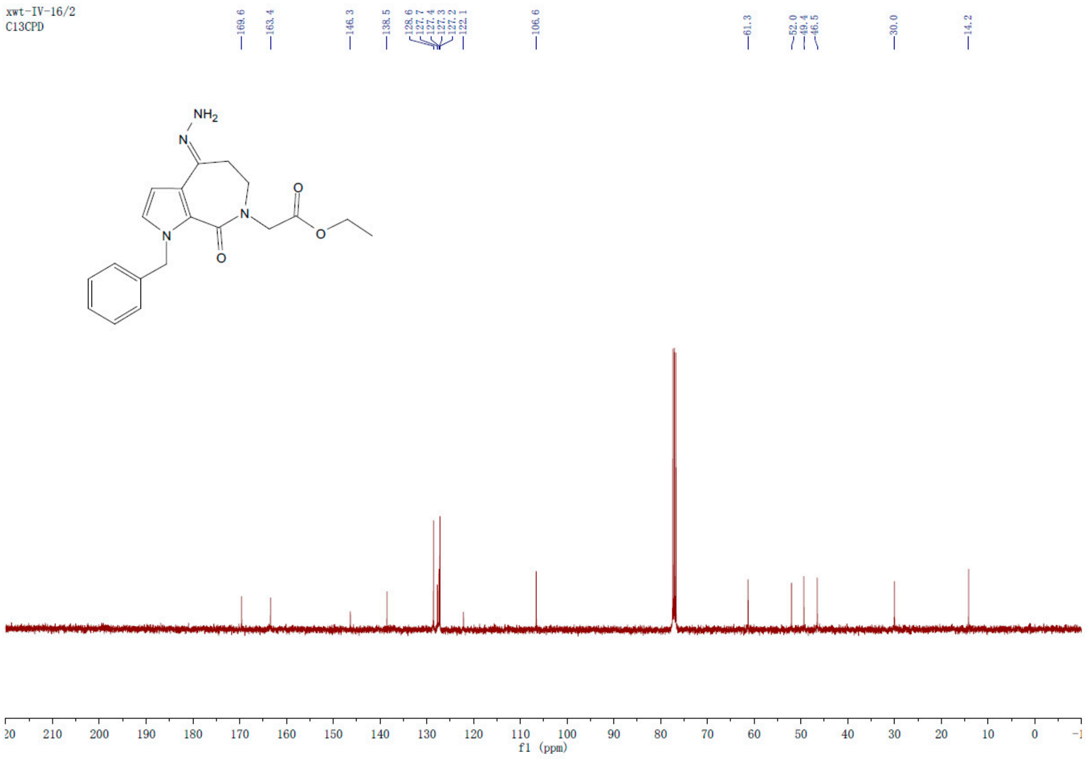

5-17 ^{13}C NMR (100 MHz, $CDCl_3$)

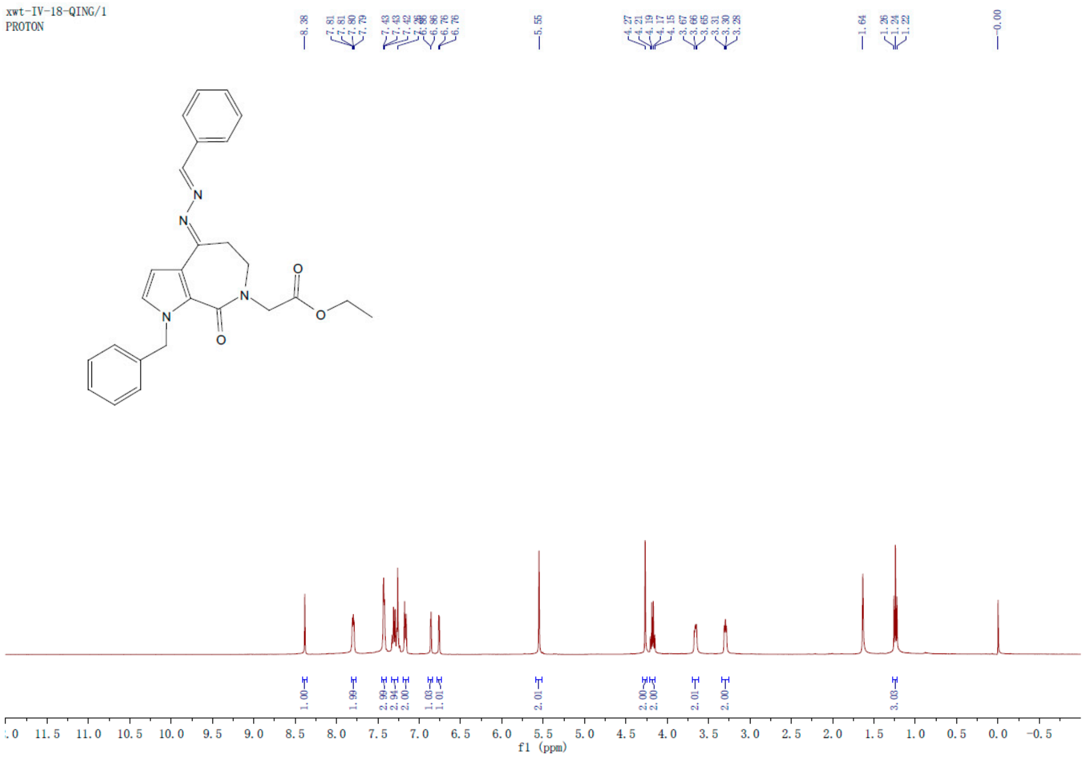

5-18 ^{1}H NMR (400 MHz, $CDCl_3$)

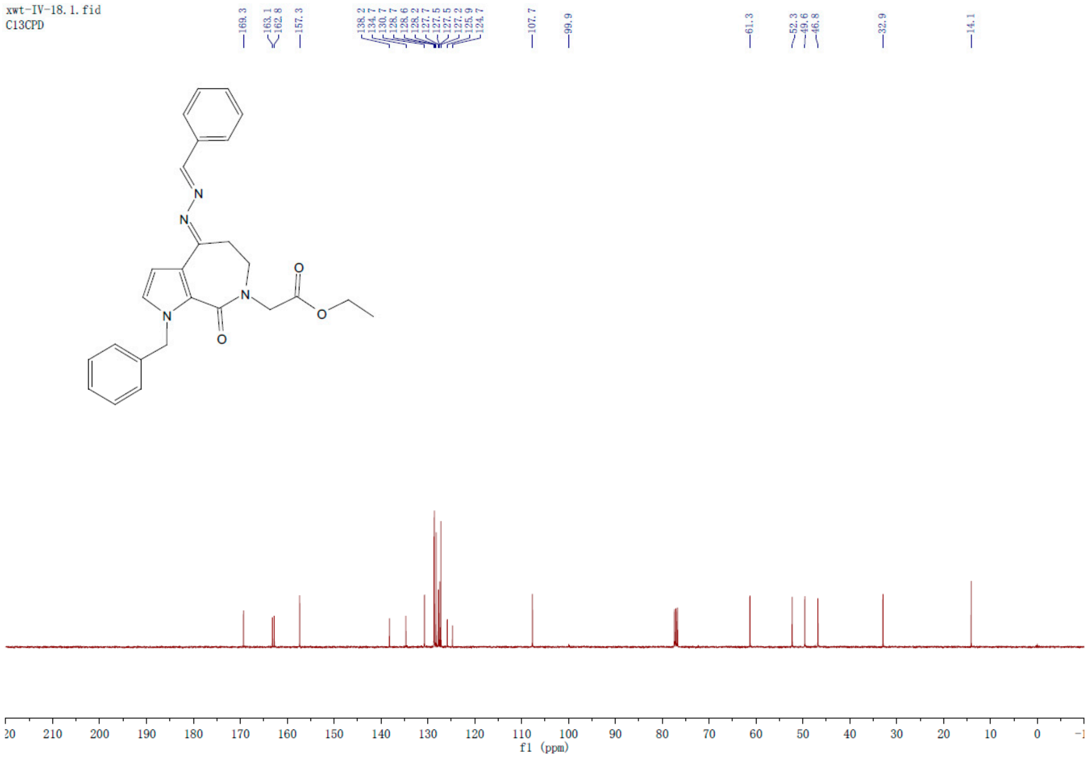

5-18 ^{13}C NMR (100 MHz, $CDCl_3$)

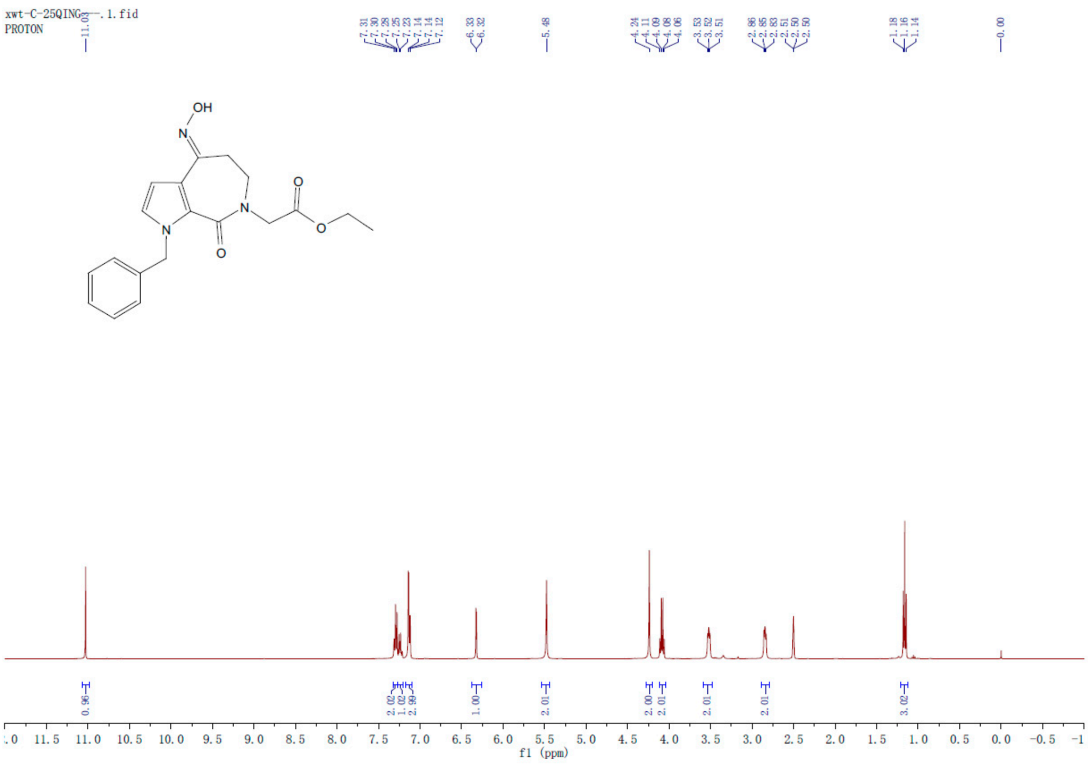

5-19 ^{1}H NMR (400 MHz, DMSO-d_6)

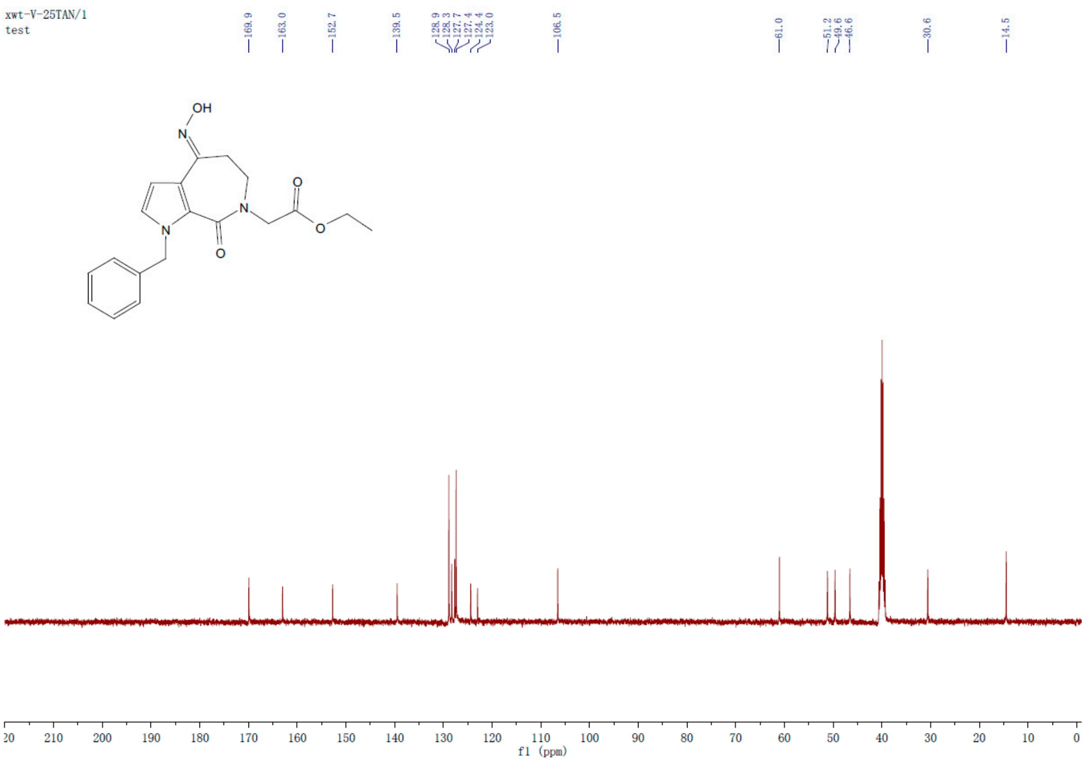

5-19 ^{13}C NMR (100 MHz, DMSO-d_6)

Detailed biological assay methods

Antiviral Biological Assay.

Purification of Tobacco Mosaic Virus.

Using Gooding's method, the upper leaves of Nicotiana tabacum L. inoculated with TMV were selected and ground in phosphate buffer and then filtered through a double-layer pledget. The filtrate was centrifuged at 10000 g, treated with PEG twice, and centrifuged again. The whole experiment was processed at 4 °C. The absorbance value was estimated at 260 nm by an ultraviolet spectrophotometer.

Virus concn = (A_{260}* dilution ratio)/$E_{1\ cm}^{0.1\%,260\ nm}$

Protective Effect of Compounds against TMV in Vivo.

The solution (100 μg/mL or 500 μg/mL) of synthesized compounds or controls was sprayed on growing Nicotiana. tabacum L. leaves (at least 3 leaves) of the same age. In another pot, the leaves were sprayed with the solvent as a control. The leaves were then inoculated with the virus (6×10^{-3} μg/mL) after 12 h by rubbing emery. First, spread a layer of 600 mesh emery on the tested leaves, then brush the leaves 1 to 2 times along the direction of the vein with a brush stained with TMV (6×10^{-3} μg/mL), and then wash the leaves with clear water. The total local lesion numbers appearing on the leaves 3−4 days after inoculation were recorded.5 There are three replicates for each compound.

Inactivation Effect of Compounds against TMV in Vivo.

To test viral inhibition, equal volumes of the virus (6×10^{-3} μg/mL) and the solution (100 μg/mL or 500 μg/mL) of synthesized compounds or controls were mixed together for 30 min. The mixture was then inoculated into the growing N. tabacum L leaves of the same age by rubbing emery, and another pot was inoculated with the

mixture of solvent and the virus (6×10^{-3} μg/mL) by rubbing emery as the control. Then, the leaves were washed with water and dried. The local lesion numbers were recorded 3−4 days after inoculation.6 There are three replicates for each compound.

Curative Effect of Compounds against TMV in Vivo.

TMV (concentration of 6.0 × 10−3 μg/mL) was inoculated on the growing leaves of N. tabacum L. of the same age by rubbing emery. Then, the leaves were washed with water and dried. The solution (100 μg/mL or 500 μg/mL) of synthesized compounds or controls was smeared on the inoculated leaves, while inoculated leaves in another pot were smeared with the solvent as a control. The local lesion numbers were recorded 3−4 days after inoculation.

There are three replicates for each compound. The in vivo inhibition rates of the compound was then calculated according to the following formula ("av" means average, and controls were not treated with the compound).

Inhibition rate (%) = [(av local lesion no. of control − av local lesion no. of drug-treated)/av local lesion no. of control] × 100%

Larvicidal activities against *Mythimna separata* and *Pyrausta nubilalis*.

The stomach toxicities of the title compounds against *Mythimna separata* were tested according to the leaf-dip method using the reported procedure. Leaf disks (about 5 cm) were cut from fresh corn leaves and then dipped into the test solution for 3-5 s. After air drying, the treated leaf disks were placed individually into a glass-surface vessel (7 cm). Each dried treated leaf disk was infested with 10 third-instar *Mythimna separata*. Percentage mortalities were evaluated 4 days after treatment. Leaves treated with acetone were provided as controls. Each treatment was performed three times.

Larvicidal Activities against diamondback moth (*Plutella xylostella*).

Stock solutions of each test compound were prepared in dimethylformamide at a concentration of 600 mg L^{-1} and then diluted to 200 mg $L^{-1}$with water containing TW-20. The leaf-dip method was used. Leaf discs (5 cm × 3 cm) were cut from fresh cabbage leaves (or other leaves) and then dipped into the test solution for 3 s. After air-drying, the treated leaf discs were placed individually into a vertical tube (or Petri dishes) and the discs were infested with second instar larvae. Percentage mortalities were evaluated 3 days after treatment. Evaluations were based on a percentage scale of 0 – 100, where 0 equals no activity and 100 equals total kill. Each treatment was performed three times. The error of the experiments was about 5%.

Detailed bioassay procedures for the fungicidal activities

The fungicidal activities of compounds were evaluated in mycelial growth tests conducted in artificial media against 14 plant pathogens at a rate of 50 μg/mL. Each test compound was dissolved in a suitable amount of acetone and diluted with water containing 0.1% TW-80 to a concentration of 500 μg/mL. To each petri dish was added 1 mL of the test solution and 9 mL of culture medium to make a 50 μg/mL concentration of the test compound, while in another petri dish was added 1 mL distilled water containing 0.1% TW-80 and 9 mL of culture medium as a blank control. A 4 mm diameter of hyphal growth was cut using a hole puncher on a growing fungal culture and the hyphae were moved to the petri dish containing the test compound. Each assay was performed three times. The dishes were stored in controlled environment cabinets (24±1°C) for 4 days, after which the diameter of mycelial growth was measured and the percentage inhibition was calculated using the following equation: Percentage inhibition (%) = (averaged diameter of mycelia in blank controls – averaged diameter of mycelia in medicated tablets) / (averaged diameter of mycelia in blank controls – 4

mm) × 100.

Calculation procedures for molecular docking research

The calculation procedures for molecular docking research consist of four steps.

Receptor Preparation.

The 3D crystal structure of TMV-CP (PDB code:1EI7) was downloaded from the protein data bank (PDB) and this was used as the receptor for molecular docking. Water molecules were removed from the target protein and hydrogen atoms were added using AutoDock Tools prior to molecular docking.

Ligand preparation.

Target compounds are drawn using ChemOffice 2019 as ligands followed by management of its conformer and the minimization process.

Molecular Docking Using AutoDock 4.2.

The input files for AutoDock were prepared using AutoDock Tools. The protein was placed in a grid box (grid parameters: center x = 0.513, center y = -24.547, center z = 0.178, size x = 12, size y = 12, size z= 12), using AutoDock to define the binding site. The docking procedure was performed using the instructed command prompts.

Analyzing and Output Visualisation using PyMOL.

The docking poses were ranked according to their docking scores. The scoring function in Auto Dock was used to predict the binding affinity of one ligand to the receptor molecule. The conformation with the lowest binding affinity was selected for further analysis after the docking process. The docking results included the locations of hydrogen bonds and closely interacting residues performed by PyMOL software.
